# Developmental differences in genome replication program and origin activation

**DOI:** 10.1093/nar/gkaa1124

**Published:** 2020-12-02

**Authors:** Cathia Rausch, Patrick Weber, Paulina Prorok, David Hörl, Andreas Maiser, Anne Lehmkuhl, Vadim O Chagin, Corella S Casas-Delucchi, Heinrich Leonhardt, M Cristina Cardoso

**Affiliations:** Department of Biology, Technical University of Darmstadt, 64287 Darmstadt, Germany; Department of Biology, Technical University of Darmstadt, 64287 Darmstadt, Germany; Department of Biology, Technical University of Darmstadt, 64287 Darmstadt, Germany; Department of Biology II, LMU Munich, 81377 Munich, Germany; Department of Biology II, LMU Munich, 81377 Munich, Germany; Department of Biology, Technical University of Darmstadt, 64287 Darmstadt, Germany; Department of Biology, Technical University of Darmstadt, 64287 Darmstadt, Germany; Institute of Cytology, Russian Academy of Sciences, St. Petersburg, Russia; Department of Biology, Technical University of Darmstadt, 64287 Darmstadt, Germany; Department of Biology II, LMU Munich, 81377 Munich, Germany; Department of Biology, Technical University of Darmstadt, 64287 Darmstadt, Germany

## Abstract

To ensure error-free duplication of all (epi)genetic information once per cell cycle, DNA replication follows a cell type and developmental stage specific spatio-temporal program. Here, we analyze the spatio-temporal DNA replication progression in (un)differentiated mouse embryonic stem (mES) cells. Whereas telomeres replicate throughout S-phase, we observe mid S-phase replication of (peri)centromeric heterochromatin in mES cells, which switches to late S-phase replication upon differentiation. This replication timing reversal correlates with and depends on an increase in condensation and a decrease in acetylation of chromatin. We further find synchronous duplication of the Y chromosome, marking the end of S-phase, irrespectively of the pluripotency state. Using a combination of single-molecule and super-resolution microscopy, we measure molecular properties of the mES cell replicon, the number of replication foci active in parallel and their spatial clustering. We conclude that each replication nanofocus in mES cells corresponds to an individual replicon, with up to one quarter representing unidirectional forks. Furthermore, with molecular combing and genome-wide origin mapping analyses, we find that mES cells activate twice as many origins spaced at half the distance than somatic cells. Altogether, our results highlight fundamental developmental differences on progression of genome replication and origin activation in pluripotent cells.

## INTRODUCTION

DNA replication, together with DNA transcription and repair, is a fundamental nuclear metabolic process. Complete and error-free genome duplication once every cell cycle is essential for genome integrity and maintenance. In eukaryotic cells, DNA replication can be subdivided in two main stages: recognition and subsequent licensing of origins of replication (ORIs) at the transition from mitosis (M-phase) to the gap 1 (G1) phase (1,2), and the activation of only a subset of these origins at the beginning of the synthesis (S) phase. The latter is eventually followed by the duplication of the (epi)genetic information by the multi-protein DNA synthesis complex (replisome) (3,4). After the initial unwinding of the DNA replication bubble at the origin of replication, the replisome ensures the semi-conservative duplication of the underlying DNA template (reviewed in (5)). Many features of DNA replication organization share high similarities between different species, including yeast, fruit flies, mice and humans (6–10), and homologues for the key factors involved have been identified in most of these species (3). Genome duplication follows a spatio-temporal program generally correlating with transcriptional activity, specific epigenetic marks and 3D genome architecture (11). Cytological methods relying on the detection of components of the replisome or nascent DNA via incorporation of modified nucleotides, allow the *in situ* visualization of newly synthesized DNA and sites of ongoing DNA replication (replication foci (RFi)). These replication foci form distinct spatial patterns, characterized by the dynamic intra-nuclear distribution of the replication signals during S-phase progression (5–7,12–14). In metazoan cells, three main patterns are observed, at early (I), mid (II) and late (III) S-phase, corresponding to the replication of euchromatin, facultative and constitutive heterochromatin, respectively (10,15,16). Fluorescence recovery after photobleaching (FRAP) experiments showed the *de novo* assembly of replisomes adjacent to previously activated sites (17), suggesting that, instead of persisting as permanent factories throughout S-phase (18), replisomes are activated in a ‘next in-line’ manner (domino model). Hence, complete DNA replication depends on the initial stochastic activation/spontaneous firing of a few origins with high firing probability within euchromatic regions of each chromosome and the subsequent domino-like activation of adjacent origins with decreasing firing probability during S-phase progression (19–22).

In early autoradiographic fiber studies of single DNA molecules it was observed that replicons resulted from individual initiation events at origins of replication, which are organized and activated in clusters of, on average, 1 Mb in size and consisting of 2–9 smaller replicons of 100–200 kb (6,23,24). DNA halo analysis showed that these replicon sizes are in good agreement with measured sizes of chromatin loops. Hence, loop structures, potentially mediated by cohesins or functionally related proteins (25,26), represent the DNA element that defines replicons as functional unit in the DNA replication context (reviewed in (27)). Labeling cells with modified nucleotides revealed that the replicon clusters observed on DNA fibers become visible as the before-mentioned replication foci in interphase nuclei (15). With higher optical resolution levels, the number of replication foci measured in cells increased and each replication nanofocus in somatic mammalian cells was shown to be equivalent to a replicon unit (28,29). Besides loop structures, chromatin signatures and the associated changes in chromatin structure and accessibility, influence licensing and activation of origins of replication and, thus, replication timing programs in mammalian cells (30). In that regard, major changes in DNA replication timing have been correlated with changes in histone acetylation levels, as histone hyperacetylation was shown to advance origin firing and DNA replication timing (31–34).

DNA replication studies in early developmental stages of *Drosophila* and *Xenopus* embryos revealed very rapid cell divisions with no gap phases and short S-phase duration (35–37). The latter is based on high origin activation levels, short inter-origin distances and concomitant differences in replicon sizes (36–38). While early developing mouse cells do not exhibit such fast cell division rates, they are characterized by short gap phases and the (almost complete) absence of transcription in the first zygotic cleavage stage (3,35–37,39–41). Additionally, specific spatial replication patterns already exist at the one-cell stage in mouse embryos (42) and replication programs of differentiating cells undergo large rearrangements during lineage commitment (43,44).

Here, we analyzed the replication dynamics in pluripotent mouse embryonic stem (mES) cells by characterizing the replication timing program and the replicon organization, and ultimately comparing it to known features of mouse somatic cells. We demonstrate that mES cells exhibit a distinct replication timing in comparison to differentiated and somatic cells, marked by early/mid replication of pericentromeric heterochromatin. We further show that this changes during mES cell differentiation when pericentromeric heterochromatin becomes late replicating. This major change correlates with differences in chromatin compaction and histone acetylation levels and can be emulated by targeting histone deacetylases to pericentromeric heterochromatin. Analysis of the replication timing of (sub)chromosomal elements, revealed in addition a synchronous replication of the Y chromosome that concomitantly marks the end of S-phase in mES cells, as well as in differentiated and somatic cells. Using a combination of molecular and super-resolution imaging techniques, we characterized the mES cell replicon, the essential functional unit of DNA replication. We found by DNA combing and genome-wide origin profiling data analyses that mES cells activate more origins of replication compared to somatic cells resulting in shorter inter-origin distances, which in turn leads to smaller replicon sizes. Furthermore, in contrast to human somatic cells, duplication of the mES cell genome relies on a substantial amount of single (unidirectional) replication forks.

## MATERIALS AND METHODS

### Expression constructs

To generate the expression vector containing a fusion protein of eGFP and human HDAC1 (peGFP-hHDAC1, pc2447), hHDAC1 was amplified from human cDNA with the following primers containing BglII and Eco47III restriction enzyme sites: fw: 5′- AA**AGATCT**AGCAAGATGGCGCAGACGCAG-3′ and rev: 5′-AA**AGCGCT**GGGCCAACTTGACCTCCTCC-3′. PCR amplicon and pCR2.1-TOPO vector (Thermo Fisher Scientific, Walham, MA, USA) were double digested with BglII and Eco47III and ligated. The final peGFP-hHDAC1 was generated by double digesting pCR2.1-TOPO-hHDAC1 with BglII and XhoI and hHDAC1 was inserted into the BglII/SalI digested backbone vector pEGFP-C1 (pc0592, Clontech Laboratories, Mountain View, CA, USA). SalI and XhoI form compatible ends that allow ligation. The final plasmid eGFP-hHDAC1 was verified by restriction enzyme digest, sequencing and enzymatic activity was analyzed via histone acetylation stainings (H4K5ac and H4K8ac, Figure 3B) in transfected cells.

All plasmid characteristics are summarized in Supplementary Table S1.

### Cell culture, differentiation and transfection

All cells used were mycoplasma free. J1 (45) and E14 (46) mouse embryonic stem cell lines were cultured in Dulbecco's modified Eagle's medium (DMEM) high glucose (Cat. No.: D6429, Sigma-Aldrich Chemie GmbH, Steinheim, Germany) supplemented with 15% fetal calf serum (FCS), 1× non-essential amino acids (Cat. No.: M7145, Sigma-Aldrich Chemie GmbH, Steinheim, Germany), 1× penicillin/streptomycin (Pen/Strep) (Cat. No.: P4333, Sigma-Aldrich Chemie GmbH, Steinheim, Germany), 1× l-glutamine (Cat. No.: G7513, Sigma-Aldrich Chemie GmbH, Steinheim, Germany), 0.1 mM beta-mercaptoethanol (Cat. No.: 4227, Carl Roth, Karlsruhe, Germany), 1000 U/ml recombinant mouse LIF (Millipore) and 2i (1 μM PD032591 and 3 μM CHIR99021 (Cat. Nos.: 1408 and 1386 respectively, Axon Medchem, Netherlands)) on gelatin-coated culture dishes (0.2% gelatin; Cat. No.: G2500, Sigma-Aldrich Chemie GmbH, Steinheim, Germany). Culture medium was changed every day and cells were split every 2 days.

Mouse embryonic fibroblasts (MEF W8 (47)) and mouse myoblasts (C2C12 (48)) were cultured in DMEM high glucose supplemented with 1× l-glutamine, 1 μM gentamicin (Cat. No.: G1397, Sigma-Aldrich Chemie GmbH, Steinheim, Germany) and 10% and 20% FCS, respectively.

Mouse primary fibroblasts were isolated from ear tissue of adult mice (C57BL/6) sacrificed according to the animal care and use regulations (Government of Hesse, Germany). The tissue was shaken at 37°C with 2 mg/ml collagenase NB8 (Serva Electrophoresis, Heidelberg, Germany) in DMEM supplemented with 1× Pen/Strep and vortexed repeatedly. Dissociated ear tissue was pipetted through a cell strainer (Cat. No.: 352235, Corning, NY, USA), single cells were plated on a gelatin-coated (0.2%) dish and cultivated in DMEM supplemented with 10% FCS and 1 μM gentamicin at 37°C and 5% CO_2_.

Differentiation of naïve pluripotent stem cells was performed as previously described (49) with the exception that the differentiation medium contained 10 μM retinoic acid (Cat. No.: R2625, Sigma-Aldrich Chemie GmbH, Steinheim, Germany).

For live cell experiments, J1 mES cells were co-transfected with plasmids encoding for mRFP-tagged hPCNA (50) and MaSat-GFP (51) using AMAXA nucleofection (B016, Amaxa Nucleofector II, Lonza Ltd., Basel, Switzerland) and plated on gelatin-coated p35 dishes containing a glass bottom that allowed for optical imaging.

For HDAC1 targeting to chromocenters, J1 mES cells were co-transfected with plasmids encoding for GBP-MaSat (52) and eGFP-hHDAC1/eGFP-C1 using AMAXA as described above. Twenty-four hours after transfection, cells were pulse chased and EdU, PCNA and histone modifications were detected as described below.

All cell line and plasmid characteristics are summarized in Supplementary Tables S1 and S2, respectively.

### DNA replication labeling and (immuno)fluorescent visualization

For immunostaining experiments, cells were grown on glass coverslips coated with 0.2% gelatin.

To visualize and analyze progression of DNA replication, cells were labeled with 10 μM 5-ethynyl-2′-deoxyuridine (EdU) for 12 min, chased for varying times with medium supplemented with 50 μM thymidine, fixed with 3.7% formaldehyde/1× phosphate-buffered saline (PBS) (Cat. No.: F8775, Sigma-Aldrich Chemie GmbH, Steinheim, Germany) for 10 min and permeabilized with 0.5% TritonX100 in 1× PBS for 20 min. All washing steps were performed with PBS-T (1× PBS/0.02% Tween-20). For detection of PCNA, cells were further incubated for 5 min in ice-cold methanol on ice for antigen retrieval. Blocking (1% bovine serum albumin, 0.5% fish skin gelatin and 0.02% Tween-20 in 1× PBS) was performed for 30 min at room temperature. EdU was detected using the Click-IT assay as described by the manufacturer (1:200 3-azido-7-hydroxycoumarin, 1:1000 6-carboxyfluorescin (6-FAM azide) or 1:2000 5/6-Sulforhodamine azide; Cat. No.: 7811, 7806 and 7776 respectively, Carl Roth, Karlsruhe, Germany). Primary mouse anti-PCNA and secondary donkey anti-mouse IgG conjugated with Cy3 were diluted in blocking buffer and incubated for 1 h at room temperature. DNA was counterstained with DAPI (4′,6-diamidino-2-phenylindole, 10 μg/ml, Cat. No.: D27802, Sigma-Aldrich Chemie GmbH, Steinheim, Germany) for 10 min and samples were mounted in Mowiol4-88 (Cat. No.: 81381, Sigma-Aldrich Chemie GmbH, Steinheim, Germany) containing 2.5% DABCO (1,4-diazabicyclo[2.2.2]octane, Cat. No.: D27802, Sigma-Aldrich Chemie GmbH, Steinheim, Germany).

To analyze replication foci in 3D super-resolution microscopy (3D-SIM), samples were prepared as described (28,53). Briefly, cells were seeded on gelatin-coated high precision coverslips (Cat. No.: LH22.1, Carl Roth, Karlsruhe, Germany), grown for 6–8 h to ensure attachment but avoid 3D colony formation, and labeled with 100 μM 5-bromo-2′-deoxyuridine (BrdU) for 25 min followed by a thymidine chase (50 μM) of 0, 30 or 60 min. Fixation and prestaining treatments were performed as described above. BrdU was detected with a rat anti-BrdU antibody diluted in buffer consisting of a 1:1 mixture of blocking and 2× DNase I reaction buffer (60 mM Tris/HCl pH 8.1, 0.66 mM MgCl_2_, 1 mM beta-mercaptoethanol) and 25 U/ml DNase I (Cat. No.: D5025, Sigma-Aldrich Chemie GmbH, Steinheim, Germany). Samples were incubated for 1 h at 37°C and DNase I digestion was stopped by washing with PBS–TE (PBS-T with 1 mM EDTA). PCNA detection was done as described above. After secondary antibody incubation with donkey anti-rat IgG AlexaFluor 488 and donkey anti-mouse IgG AlexaFluor 594, samples were mounted in Vectashield (Invitrogen, Carlsbad, CA, USA).

All nucleotide and antibody characteristics are summarized in Supplementary Tables S3 and S4, respectively.

### Immunofluorescence

For pluripotency marker detection, cells were grown, fixed, permeabilized and blocked as described for DNA replication visualization. Primary mouse anti-Oct3/4, rabbit anti-Sox2 and secondary goat anti-rabbit IgG Alexa647 and goat anti-mouse IgG Alexa647 were diluted in blocking buffer and applied for 1 h at room temperature.

For histone modification analysis, cells were grown, EdU labeled, fixed, permeabilized and blocked as described above. EdU was detected as described by the manufacturer (1:1000 6-FAM azide). The following primary and secondary antibodies were diluted in blocking buffer: mouse anti-H3K9ac, rabbit anti-H3K9m3, rabbit anti-H4K5ac, rabbit anti-H4K8ac, donkey anti-mouse IgG Cy3 and donkey anti-rabbit IgG Cy3. Incubation was done for 1 h at room temperature.

DNA counterstaining and mounting using Mowiol was performed as described above.

All nucleotide and antibody characteristics are summarized in Supplementary Tables S3 and S4, respectively.

### Major satellite (MaSat) polydactyl zinc finger (PZF) fixation

Since transfected MaSat-GFP (pc1803 and (51)) is not fixable with standard formaldehyde or methanol fixation protocols, we made use of a gradient fixation protocol combined with a simultaneous mild permeabilization on ice. Twenty-four hours after transfection with MaSat-GFP, J1 mES cells were put on ice and 3.7% formaldehyde/1× PBS with 0.1% Nonidet™ P 40 Substitute (Cat. No.: 74385, Sigma-Aldrich Chemie GmbH, Steinheim, Germany) was added to the medium to achieve a final concentration of 0.1% formaldehyde. After 10 min, formaldehyde concentration was increased to 0.2% and incubated for 10 min. This procedure was repeated 6 times (0.5%, 1%, 1.5%, 2%, 2.5%, 3% formaldehyde). Increasing formaldehyde concentration to 1.5% was performed with a 0.1% Nonidet P 40 substitute containing formaldehyde stock solution. In a final step, the medium/formaldehyde mixture was replaced by 3.7% formaldehyde (10 min incubation) and exchanged with PBS-T.

### Molecular combing

Molecular combing experiments were performed using the FiberPrep^®^ kit (Cat.No.: EXTR-001, Genomic Vision, Bagneux, France) and as described before (54). Briefly, J1 mES cells were pulse labeled for 15 min with 10 μM 5-chloro-2′-deoxyuridine (CldU), washed twice with pre-warmed PBS, labeled with 100 μM 5-iodo-2′-deoxyuridine (IdU) for 15 min, washed extensively and chased for 1 h with 50 μM thymidine. Cells were subsequently embedded in low-melting point agarose, genomic DNA was isolated by proteinase K (Cat. No.: 7528, Carl Roth, Karlsruhe, Germany) digestion and single high molecular weight DNA molecules were stretched on silanized glass coverslips (Cat. No.: COV-002-RUO, Genomic Vision, Bagneux, France), using the FiberComb^®^-Molecular Combing System (Cat. No.: MCS-001, Genomic Vision, Bagneux, France) as described by the manufacturer. Incorporated nucleotides and single stranded DNA were detected using mouse anti-BrdU/IdU, rat anti-BrdU/CldU and mouse anti-single stranded DNA (IgG2a) primary antibodies and goat anti-mouse IgG Chromeo 546, donkey anti-rat IgG AlexaFluor 488 and goat anti-mouse IgG2a AlexaFluor 647 secondary antibodies.

All nucleotide and antibody characteristics are summarized in Supplementary Tables S3 and S4, respectively.

### Probe generation, metaphase and (Repli-)FISH (fluorescence *in situ* hybridization)

Probes against major satellites, minor satellites and telomeres were generated as described in (55).

The Y chromosome probe was generated via DOP-PCR (degenerated oligonucleotide-primed-PCR). For template stock generation, PCR reactions contained mouse Y chromosome-specific template DNA (kind gift of Prof. Dr. Diane Krause, Yale University School of Medicine), 2 μM 6AI primer (5′-CCGACTCGAGNNNNNNTACACC-3′), 0.25 mM dNTPs and 2.5 U Taq polymerase in 1× PCR buffer (10 mM Tris/HCl pH 8.3, 50 mM KCl and 1.5 mM MgCl_2_) and cycling conditions were set to (45″ at 94°C, 45″ at 15°C, 12′ at 37°C) ×1, (40″ at 94°C, 45″ at 37°C, 4′ at 66°C) ×5 and (40″ at 94°C, 45″ at 54°C, 4′ at 66°C) ×24. Y chromosome template DNA was labeled with biotinylated nucleotides (55) in the following reaction: template stock DNA was mixed with a nucleotide mixture containing unlabeled nucleotides (0.2 mM each dATP, dCTP and dGTP with 0.1 mM dTTP), biotinylated dUTPs (0.1 mM biotin-16-dUTPs), 2 μM 6AI primer, 2.5 U Taq polymerase and 1× PCR buffer. Cycles were set to (5′ at 94°C) ×1, (30″ at 94°C, 30″ at 54°C, 90″ at 72°C) ×35 and (5′ at 72°C) ×1.

Metaphase FISH and co-visualization of DNA replication and DNA probes were performed as previously described (55), using rabbit anti-digoxigenin, anti-rabbit IgG Cy3 antibodies and Streptavidin Alexa488 or Streptavidin Cy5 (1:500, Cat. No.: S11223 and SA1011, Thermo Fisher Scientific, Waltham, MA, USA).

Triple FISH was performed by a combination of biotinylated, Cy3 and Cy5 labeled probes.

To visualize MaSat repeat sequences in MaSat-GFP transfected cells, cells were fixed using a gradient fixation protocol described above and FISH was done as described above. Since heating GFP expressing cells to 80°C dramatically reduced GFP fluorescence, cells were stained with a mouse anti-GFP antibody to re-visualize MaSat-GFP. After probe annealing and washing, cells were first incubated with the anti-GFP primary antibody followed by the secondary donkey anti-mouse Alexa488 antibody and Streptavidin Cy5 incubation for 1 h in blocking buffer (1% bovine serum albumin, 0.5% fish skin gelatin and 0.02% Tween-20 in 1× PBS).

All nucleotide and antibody characteristics are summarized in Supplementary Tables S3 and S4, respectively.

### Karyotype analysis

To prepare J1 mES cell metaphase spreads, cells were arrested in mitosis by adding 0.02 μg/ml colcemid (Cat. No.: 10 295 892 001, Roche Diagnostics GmbH, Basel, Switzerland) for 1.5 h at 37°C. The supernatant and harvested cells were pelleted by centrifugation for 5 min at 300 × g, cells were resuspended in 10 ml pre-warmed hypotonic solution (0.075 M KCl) and incubated for 6 min at 37°C. After centrifugation for 5 min at 300 × g, cells were fixed by dropwise addition of fixative solution (3:1 methanol:acetic acid) and incubation for 45 min on ice. Etched microscope slides were prepared by submerging the slides for 15–20 min in etching solution (0.1 N HCl in 95% ethanol) followed by cleaning steps in 95% EtOH and ddH_2_O (3 times each). Finally, spreads were generated by dropping fixed cells onto etched slides and air-drying. Individual metaphase spreads were imaged by phase-contrast microscopy and analyzed manually.

### Doubling time and S-phase duration

For growth curve analysis, 2 × 10^5^ J1 mES cells were seeded as technical quadruplicates at day 0 and cell numbers were counted with a Neubauer haemocytometer for four consecutive days. Population doubling times were derived with log_2_(*n_x_*/*n*_0_)/*t* (h) (*n_x_*: cell number at day *x*, *n*_0_: cell number at day 0, *t*: hours after seeding). To determine the percentage of cells in every cell cycle and S-phase substage, asynchronously growing J1 cell cultures were pulse labeled with 10 μM EdU for 12 min, fixed and EdU was detected as described. Cells were manually grouped into S-phase substages (stage I to Y), non S-phase or mitosis, and percentages were calculated. S-phase (substage) duration was derived by multiplying the doubling time with the percentage of cells in the respective phase.

### Microscopy

All characteristics of the microscopy systems, including lasers, filters and objectives used, are summarized in Supplementary Table S5.

Molecular combing samples were imaged using a Zeiss Axiovert 200 widefield microscope.

Confocal z-stacks of live cells were acquired using the Ultra-View VoX spinning disk microscopy system. Time-lapse microscopy was carried out in a closed live-cell microscopy chamber at 37°C, with 5% CO_2_ and 60% humidity. mRFP-PCNA and MaSat-GFP double transfected J1 mES cells were imaged every 30 min for 24 h to follow cell cycle progression.

Confocal z-stacks were acquired with a Leica TCS SP5 II confocal laser scanning microscope or the spinning disk microscope.

3D SIM images were acquired with a DeltaVision OMX V3 system (56).

### Image analysis

Image analysis was done using ImageJ (http://rsb.info.nih.gov/ij/, v1.51s and earlier), Volocity 6.3 (Perkin Elmer), ilastik (57) (https://www.ilastik.org, v1.3.3post3) and Python with the scipy-stack and scikit-image (58) (Anaconda distribution 2020.07).

#### Quantitative analysis of replication foci features

EdU labeled RFi in manually cropped confocal image stacks of mES cells were segmented by supervised pixel classification using ilastik (57). A subset of pixels was manually annotated as belonging to the background or to a replication focus (RF) and used to train a random forest classifier on pixel features to propagate the classification to the remaining pixels in all images. Similarly, a 3-class (background/nuclear border/nuclear interior) classifier was created for generation of nuclear marks. To separate touching nuclei, the center of each individual nucleus was manually labelled in the cropped images. Using Python and scipy/scikit-image (58), an instance segmentation was created via marker-controlled watershed using these manual markers and the probability of the ‘border’ class as the ridge image. For each crop, only the nucleus with centroid closest to the image center was used for the subsequent steps. The RFi mask was used to generate features for each RF in the EdU channel. Touching objects were separated by a watershed transform on the Euclidean distance transform (EDT) of the mask. For feature calculation, the stacks were scaled in z to achieve isotropic resolution. For each object overlapping more than 50% with the nuclear mask, a series of features were calculated (Supplementary Figure S1). RF objects smaller than 200 px^3^ (∼1 confocal PSF) were discarded. For each image, a single feature vector consisting of medians and standard deviation of the RFi features (see Supplementary Figure S1 for complete list of analyzed parameters), as well as the number of RFi and the chase duration was created.

Further analysis was performed using scikit-learn. Missing features were filled with the mean value of that feature in all images. The feature vectors were normalized to zero mean and unit variance and visualized in a 2D embedding via *t*-distributed stochastic neighbor embedding (*t*-SNE).

The analysis pipeline is summarized in Supplementary Figure S1 and the Python code for the pipeline is available at https://doi.org/10.25534/tudatalib-220.

#### Replication signal and chromocenter colocalization analysis

To determine the degree of colocalization of replication signals (EdU) and constitutive heterochromatin (chromocenters) in differentiated mES cells, cells were grouped into early, mid and late replicating cells (*S*_e_, *S*_m_ and *S*_l_) according to their EdU pattern. Chromocenters were segmented based on the DAPI signal, EdU signal intensities within the segmented regions were measured and EdU intensities in chromocenters were plotted for *S*_e_, *S*_m_ and *S*_l_ cells.

#### Histone modification analysis

To determine the histone modification accumulation at chromocenters, cells were segmented at single mid planes according to the DAPI channel and DAPI intensities were measured. S-phase cells were identified based on the EdU signal and cells were grouped into G1, S and G2 phases (intensity[DAPI]_G1_ < intensity[DAPI]_S_ < intensity[DAPI]_G2_) (20). For subsequent histone modification analysis, only G1 phase cells were considered. Within the DAPI channel, four circular regions of interest (ROI) were drawn inside DAPI intense chromocenters at mid planes, and four ROIs outside chromocenters. Histone modification levels were measured inside the eight ROIs. The analysis pipeline is summarized in Supplementary Figure S2. Mean intensity averages were calculated for the four regions inside chromocenters (chromocenter) and accordingly for regions outside chromocenters (nucleoplasm). Histone modification accumulation at chromocenters was determined as a ratio of chromocenter/nucleoplasm.

To determine histone modification changes after HDAC1 targeting to chromocenters, histone acetylation levels were measured in mid focal planes of individual transfected cell nuclei. Background in the histone channel was subtracted and fluorescence intensities were measured. Results are plotted as a ratio to GFP control cells.

#### Chromocenter characteristic analysis

Images of DAPI stained nuclei of (un)differentiated J1 mES cells and primary mouse ear fibroblasts were imported into Volocity 6.3 (Perkin Elmer). Pericentromeric heterochromatin (chromocenters) were segmented based on fluorescence intensity and the total number of chromocenters per cell as well as the volume, the degree of compaction and the shape factor of every chromocenter were calculated.

#### Repli-FISH analysis

To determine the replication timing of specific (sub)chromosomal elements, 3D masks of individual cell nuclei were manually generated based on the DAPI counterstaining (single FISH) or the EdU staining (triple FISH). FISH signals were segmented independently after applying a Gaussian filter (sigma = 1) and subtracting background (rolling ball algorithm with radius = 10). Similarly, PCNA images were background corrected and basic PCNA signal from non replicating cells (non S-phase cells) was subtracted. The 3D nucleus mask was subsequently applied to the segmented FISH signals and the generated nuclear FISH ROIs were used to mask the PCNA signals. PCNA intensities within the mask were measured and plotted. The analysis pipeline is summarized in Supplementary Figure S3.

To analyze if the replication timing of telomeres is dependent on their 1D proximity to chromocenters, telomere signals (visualized by Repli-FISH in combination with PCNA) in stage II S-phase cells were segmented and grouped according to their location compared to chromocenters. Telomeres located on the short arm of acrocentric mouse chromosomes are in close 1D proximity of pericentromeric heterochromatin (referred to as telomeres in chromocenters), while telomeres capping the long arm of chromosomes are more distant from major satellite repeats (referred to as telomeres out of chromocenters). PCNA intensities within the segmented telomere signals were measured and plotted.

#### Replication foci analysis

Quantification of replication foci within individual cell nuclei was mainly performed as described in (28,53). In brief, 3D-SIM images were reconstructed, exported from the DeltaVision software (softWoRx 6.0 Beta 19, Applied Precision) and raw 3D-SIM images were converted to 16-bit images using a custom-written FIJI (59) macro. Individual cell nuclei were segmented using maximum intensity projections of the DAPI signal. Replication signals were segmented by auto-thresholding using the Triangle method. The resulting binary images were used to mask the original replication foci signals of interest and to discriminate them from background (set to ‘0’). These images and the corresponding DAPI images were imported to the image analysis software Volocity 6.3 (Perkin Elmer) and replication foci were quantified for individual nuclei. First, nuclear masks were generated based on the DAPI images and defined as regions of interest (ROIs). Next, 3D-SIM replication foci were detected by intensity excluding only black pixels (i.e. background with intensity ‘0’), touching foci were separated (object size guide = 0 μm^3^) and signals smaller than 0.0002 μm^3^ were excluded from the final counting as they represented unspecific background signal. Only foci within the nuclear ROI were counted. A detailed analysis pipeline is summarized in Supplementary Figure S4.

Pseudo wide-field (pseudoWF or pWF) replication signals were generated from the same datasets (C2C12 and J1 mES cells labeled with BrdU). Generation of the pseudo wide-field data was described in (60). For correlation analysis, the pseudoWF images were initially processed in ImageJ to match the image dimension of the 3D-SIM data. To achieve similar voxel sizes (40 × 40 × 125 nm), the images were scaled using a bicubic interpolation, doubling the number of pixels in *x* and *y*. Next, pseudoWF images were corrected for pixel shifts by translating the image stack –2 pixels in *x* and *y*. For segmentation of the pseudoWF replication signals, the histogram was normalized and a background subtraction was performed using a rolling ball algorithm with radius = 10. Segmentation was performed by auto-thresholding using the Otsu algorithm. 3D-SIM replication signals were processed as described above. Finally, segmented and masked pseudoWF and 3D-SIM image stacks were merged and used for foci counting in Volocity. Detection of pWF RFi was based on intensity as for 3D-SIM images, separation of touching objects was based on object size (object size guide = 0.02 μm^3^) and signals smaller than 0.02 μm^3^ were excluded. Overlapping signals used for nanoRFi counting within pWF RFi were filtered by an additional compartmentalization step. The analysis pipeline is summarized in Supplementary Figure S5.

#### DNA fiber analysis

For replication signal analysis from molecular combing experiments, fluorescent DNA fiber tracks were selected according to their pattern. Only lengths of the second pulse (CldU) of progressing forks (CldU track preceded by a clear IdU signal on fibers with a ssDNA signal up- and downstream of the marked tracks) were considered. Replication fork speed (RFS) was calculated as a ratio of the track length (track length × 2000 due to the constant stretching factor resulting in 1 μm ∼ 2000 nts) and the time of nucleotide application. Inter-origin distance (IOD) was calculated in kb as the product of the measured track length and the conversion factor of 2 (1 μm ∼ 2000 nts or 2 kb). Bidirectional fork asymmetry was analyzed as the ratio of the long track and the short track and percentage of unidirectional forks was calculated by dividing the number of unidirectional forks by the total number of analyzed forks (uni- and bidirectional forks). A graphical summary of the selection of the fiber tracks and the corresponding calculations are depicted in Figure 7A and Supplementary Figure S6.

### Genome-wide replication origin profiling

The GEO (Gene Expression Omnibus, https://www.ncbi.nlm.nih.gov/geo/) samples GSM3602315, GSM3602316 and GSM3602317 from the dataset GSE126477 (61) and samples GSM2651111 and GSM2651112 from the dataset GSE99740 (62) were used for genome-wide replication origin profiling in mES cells. The samples GSM2651107 and GSM2651108 from the dataset GSE99740 (62) were used for genome-wide replication origin profiling in MEF cells. The above-mentioned datasets correspond to five replicates of origin mapping in mES cells and two replicates in MEF cells realized by sequencing of isolated small nascent DNA strands (SNS-seq). The analysis was performed using peaks reproducibly found in at least two replicates using the multiIntersectBed command in bedtools (63).

The GEO samples GSM3227970, GSM3227971 and GSM3227972 from the dataset GSE116321 (64) were used for genome-wide replication origin profiling in activated mouse B cells using Okazaki fragment sequencing method (OK-seq). The coordinates of OK-seq replication initiation zones were kindly provided by Andre Nussenzweig and Sridharan Sriram. The mm10 reference genome (http://hgdownload.cse.ucsc.edu/goldenPath/mm10/bigZips/) was used in all data analysis. The operations on genomic intervals were performed using bedtools (63).

The IODs were calculated between the middle point of each replication origin zone identified and the distances between origins flanking chromosomal regions unmapped in sequencing analyses (centromeres, etc.) were omitted from the plots.

All genome-wide origin mapping datasets and samples are summarized in Supplementary Table S6.

### Data visualization and statistical analysis

Data visualization and statistical analysis (independent two-group student's *t*-tests and Mann–Whitney–Wilcoxon tests) were performed with *RStudio* (v1.0.143–v1.1.447, https://rstudio.com/).

Visualization of origin replication profiles was performed with IGV (Integrative Genomics Viewer, version 2.8.6, https:/software.broadinstitute.org/software/igv/).

Statistical values (number (#) of cells (*N*), mean, median, standard deviation (SD), standard error of the mean (SEM), 95% confidence interval (CI) and *P*-values) are indicated in the plots or summarized in Supplementary Tables.

Boxplots and violin plots represent the median (center line) with the box depicting the 25–75 percentiles and the lines the upper and lower whiskers with 1.5 times the IQD (inter-quartile distance) (Supplementary Figure S7). Barplots show averaged values and error bars the respective standard deviation.

All cells analyzed (*N* numbers stated in Supplementary Tables) showed the reported behavior of the representative images shown in the respective figures.

## RESULTS and DISCUSSION

### Characterization of the spatio-temporal DNA replication patterns in mouse embryonic stem cells reveals differences to somatic cells

**Figure 1.**
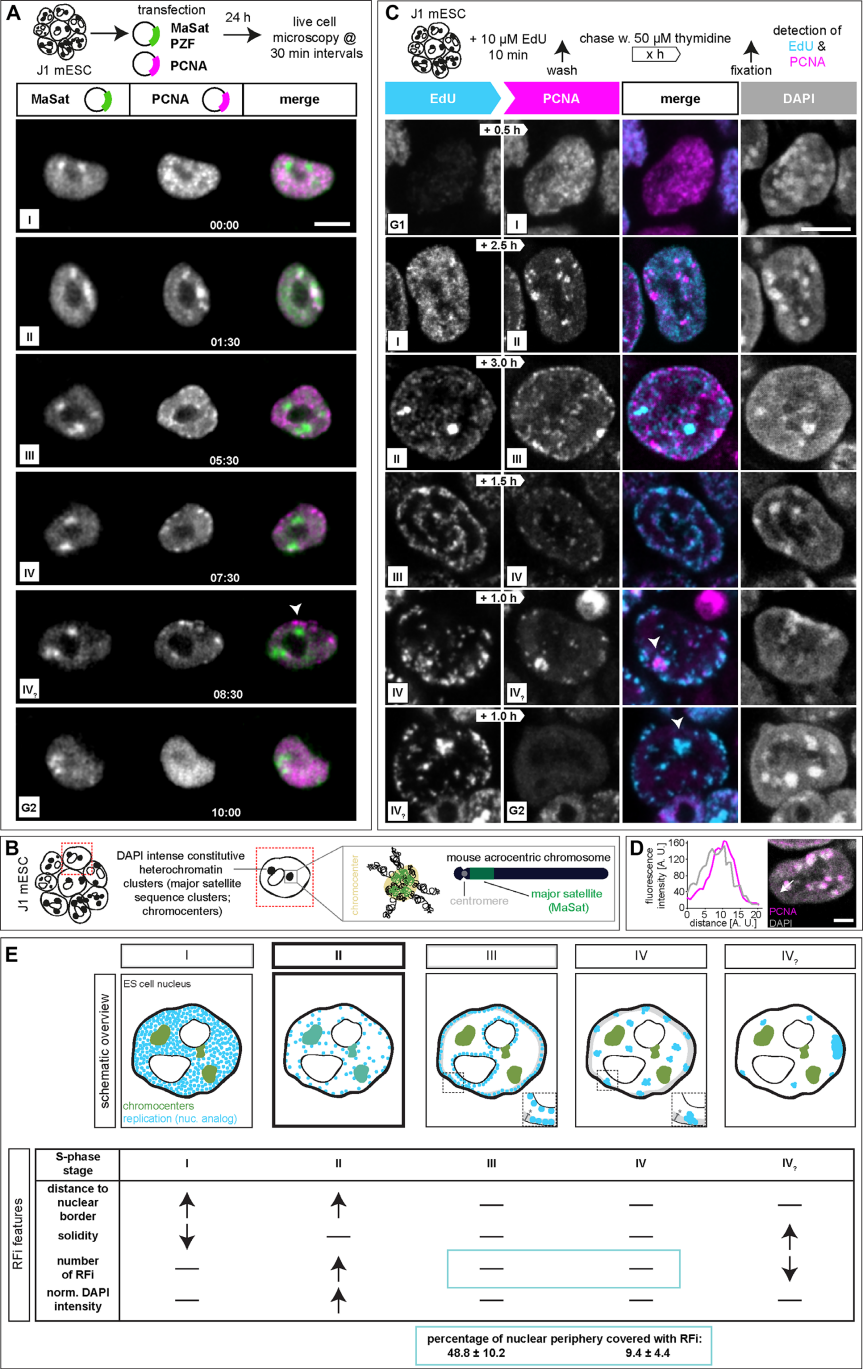
DNA replication dynamics in mouse embryonic stem cells. (**A**) Experimental setup of a live cell experiment to determine the *in vivo* spatio-temporal progression of DNA replication in J1 mES cells. Cells were transfected with plasmids encoding mRFP-PCNA (magenta) and GFP-tagged polydactyl zinc finger protein (PZF) specifically binding to major satellite repeats (MaSat-GFP, green) fusion constructs to mark ongoing DNA replication and pericentromeric heterochromatin (chromocenters), respectively. Imaging was performed for 24 h with 30 min intervals. Representative spinning disk confocal images show the cell cycle progression of a representative mES cell and S-phase was further subdivided into five main replication patterns (I–IV_?_). The arrowhead in the IV_?_ stage marks a prominent accumulation of replication signals observed at the end of S-phase. (**B**) Schematic representation of acrocentric mouse chromosome clustering in mES cell nuclei. At the chromosomal level, constitutive heterochromatin major satellite repeats (green) flank the centromere (grey) and in interphase nuclei, pericentromeric DNA from different chromosomes clusters to chromocenters. (**C**) Experimental setup of a pulse-chase experiment to determine the spatio-temporal progression of DNA replication in mouse J1 ES cells. Asynchronously growing mES cell cultures were pulse labeled with the nucleotide analog EdU, followed by various thymidine chase periods (white arrows) and fixation. EdU, i.e. nascent DNA during the first pulse labeling (cyan), and endogenous PCNA, i.e. ongoing replication at the time point of fixation (magenta), were (immuno)fluorescently detected and allowed the identification and the temporal order classification of five main replication patterns in mouse J1 ES cells. Representative spinning disk confocal images of G1 to S-phase, S-phase substage transitions (I–IV_?_) and S-phase to G2 progression are shown. The arrowheads in the IV_?_ stage mark a prominent accumulation of replication signals observed at the end of S-phase. (**D**) Line profile analysis of PCNA fluorescence intensities within one chromocenter in a stage II cell. (**E**) Schematic summary of the five replication patterns observed in mES cells. Replication signals are shown in cyan, pericentromeric heterochromatin in green and replicating chromocenters (stage II) are marked in dark cyan. Table summarizing the significant RFi features of the five S-phase substages. Grey areas in stage III and IV S-phase schematic cells represent the nuclear periphery segmented to determine the percentage of the latter covered with RFi. The width of this area (*) corresponds to the diameter of stage III peripheral RFi (0.44 ± 0.13 μm). All experiments were done in at least three independent biological replicates. Detailed statistics are summarized in Supplementary Tables S7 and S11. Scale bars = 5 μm. Dotted lines represent cell contours.

Replication patterns are a direct visual representation of the spatial organization and temporal order of DNA replication and, in somatic cells, have been shown to reflect the chromatin organization level (10,15,16). DNA replication timing profiles (RT-profiles) from large cell populations (43,65–68) revealed distinct replication domains (1.5–2.5 Mb), that exhibit sharp boundaries between neighboring domains with different replication timing, alternating along individual chromosomes (43). While RT-profiles can directly be linked to the underlying DNA sequence, they fall short on temporal resolution and do not provide 3D spatial information. Single cell microscopy analysis, however, allows a specific 4D analysis of DNA replication. Importantly, it allows to map replication timing of DNA repeat elements, which are largely not mappable by sequencing-based approaches and constitute a very large portion of mammalian genomes (69). To investigate and compare the spatio-temporal organization of DNA replication (5) of pluripotent cells, we first analyzed S-phase progression in mouse mES cells by live cell imaging experiments. Therefore, we transfected J1 mES cells with plasmids encoding for mRFP-PCNA and a GFP tagged polydactyl zinc finger protein specifically binding to major satellite sequences (MaSat PZF) and imaged the cells every 30 min for 24 h (Figure 1A). To exclude artifacts introduced by transfection and overexpression of fluorescently tagged PCNA, we validated mRFP-PCNA localization during S-phase by immunostaining with a PCNA specific antibody on transfected mES cells (Supplementary Figure S8A). Additionally, we validated binding of the MaSat PZF to major satellite repeats. Due to its high mobility and fast binding kinetics, MaSat PZF is not fixable with standard formaldehyde or methanol fixation protocols. We, therefore, established a gradient formaldehyde fixation protocol with simultaneous permeabilization, and performed fluorescence *in situ* hybridization (FISH) with a probe specifically binding to MaSat repeats. MaSat-GFP colocalized with the major satellite probe signal, as well as with DAPI intense nuclear regions (Supplementary Figure S8B). The live cell microscopy approach provided a detailed spatio-temporal analysis of S-phase progression *in vivo* and revealed visually distinguishable spatial replication patterns in mouse J1 mES cells (Figure 1A, Supplementary Figure S8C, Movies 1 and 2). At the beginning of S-phase, replication foci distributed homogeneously in the nuclear interior (stage I), possibly reflecting duplication of the euchromatic portion of the genome, as seen in somatic cells. Next, and differing from somatic cells, MaSat PZF-labeled and condensed clusters of pericentromeric heterochromatin (chromocenters, Figure 1B) were replicated (stage II). Although this observation might be unexpected in view of the late replication timing of heterochromatin in somatic cells, it has been previously shown that in *Drosophila*, satellite sequences became increasingly heterochromatic and late replicating only with successive differentiation at later developmental cycles (70). After pericentromeric heterochromatin replication, duplication of chromatin located at the nuclear and nucleolar borders was observed in mES cell (stage III), which, in somatic cells, reflects duplication of facultative heterochromatin. Next, the cells displayed a pattern with a decreased number of foci which however increased in size, suggesting clustering of the underlying chromatin fiber. Replication signals were mostly, but not exclusively, located at the nuclear periphery (stage IV). The end of S-phase was marked by a strong accumulation of replication signals within one particular region of the mES cell nucleus (stage IV_?_). Additionally, we measured the duration of the individual S-phase substages from the live cell microscopy. While early and mid S-phase (stage I and II) lasted over 3.5 and 4.5 h, respectively, the subsequent phases were relatively short, with average durations of ∼1 h each (stage III, IV and IV?, Supplementary Figure S8D and Supplementary Table S7).

Next, we validated the observed S-phase progression in mES cells by pulse-chase experiments. Asynchronously growing mouse J1 mES cells were pulse labeled for 12 min with the nucleotide analog 5-ethynyl-2′-deoxyuridine (EdU), washed to remove nucleotide excess, chased with thymidine for different periods of time and fixed. Subsequently, replication foci marked by the incorporated EdU and the replisome component PCNA were (immuno)fluorescently detected (Figure 1C and Supplementary Table S11). EdU marked DNA replicated during the nucleotide pulse, while the PCNA pattern corresponded to the active replication sites at the time of fixation of the cells. The different chase times resulted in different degrees of replication foci separation and, with increasing chase times, eventually also to transition from one S-phase pattern to the next (Supplementary Figure 9A). To achieve visualization of S-phase substage progression and, concomitantly, separation of replication patterns of the different stages (Supplementary Figure S9B), chase durations were changed according to S-phase substage duration, as obtained from the time lapse movies (Supplementary Figure S8D and Supplementary Table S7). Live cell microscopy revealed substantially longer durations of stage I and II compared to stage III and IV (Supplementary Figures S8C, S8D and Supplementary Table S7). Hence, to reflect stage to stage transitions, chase times had to be adapted accordingly. To visualize stage I to stage II transitions in a significant number of S-phase cells, chase times had to be longer than to visualize transitions from stage III to stage IV (Supplementary Figure S9B). This approach allowed us to get a spatio-temporal resolution of DNA replication in fixed cells, underlining the domino-like DNA replication model (Supplementary Figure 9C). As in the live-cell data, replication of the large heterochromatin clusters (stage II), colocalizing with DAPI intense nuclear regions (Figure 1D), took place after the early S-phase stage I pattern and was followed by the nucle(ol)ar periphery stage III replication pattern. All other features described in the live cell experiments were also reproduced with the pulse-chase approach in fixed cells.

Since mouse ES cells grow in very specific microenvironments in cell culture, which include the presence of leukemia inhibitory factor (LIF) and two inhibitors (2i, PD032591 and CHIR99021) to preserve pluripotency and self-renewal capacities, as well as form 3D colonies, we tested the influence of this stem cell specific microenvironment on the temporal organization of DNA replication. S-phase progression from stage I to stage II was observed for single mES cells (Supplementary Figure S10A) and in cells grown in the absence of the 2i (Supplementary Figure S10B), similar to cells grown in 3D colonies in the presence of 2i and LIF. Additionally, we analyzed the replication foci pattern distribution over time via the above mentioned pulse-chase experiments in mouse E14 mES cells, and found similar spatio-temporal progression as observed for J1 cells (Supplementary Figure S11) indicating a conservation of S-phase characteristics for mouse pluripotent cells.

In summary, the general characterization of the spatial distribution of replication signals during S-phase progression in mouse embryonic stem cells by pulse-chase and live cell experiments led to the identification and temporal classification of a sequence of replication patterns, which differ from somatic cells (Figure 1E).

### Quantitative features of mouse embryonic stem cell replication foci patterns

To characterize the different replication patterns observed in live cell and pulse chase experiments (Figure 1E), we determined quantitative features of the underlying replication foci (RFi) within the different S-phase substages (I–IV_?_). EdU labeled cells were classified according to their S-phase pattern, replication signals were segmented, separated using a watershed algorithm and location, shape and intensity features were determined (Figure 1E and Supplementary Figure S12). In a 2D embedding via *t*-distributed stochastic neighbor embedding (*t*-SNE) the RFi features did not form clearly separated clusters, but temporally adjacent stages lied next to each other in the embedding, hinting at a continuum in feature space from the beginning to the end of replication (Supplementary Figure S13). Stage I of mES cell S-phase was characterized by the combination of a significant increase in the distance of the RFi from the nuclear border (Figure 1E and Supplementary Figure S12) and a decrease in RFi solidity (‘rougher’). In contrast, stage II was marked by an increase in RFi numbers with a concomitant increase in DAPI intensity. This is in agreement with our findings that DAPI intense nuclear regions, i.e. chromocenters, are replicated during this stage (Figure 1A–D). Moreover, we determined specific characteristic features for stage IV_?_. At the end of S-phase, the amount of RFi decreased dramatically, whereas solidity, i.e. smoothness, increased. As RFi volumes as well as other features in stages III and IV showed no significant differences, we additionally calculated the percentage of the nuclear periphery covered by replication foci in these two stages. The size of the mask used to segment the nuclear periphery was set to the diameter of the RFi located at the nuclear border in stage III (Figure 1E). While almost half of the nuclear border exhibited RFi in stage III cells, only 10% of the periphery contained RFi in stage IV cells. Since these observations suggest a difference in the clustering of the underlying chromatin, we retained the S-phase substage division into five substages. Taken together, we identified several distinct location, shape and intensity features of replication foci that characterize and distinguish mES cell S-phase substages.

### Constitutive heterochromatin shifts its replication timing during loss of pluripotency

**Figure 2.**
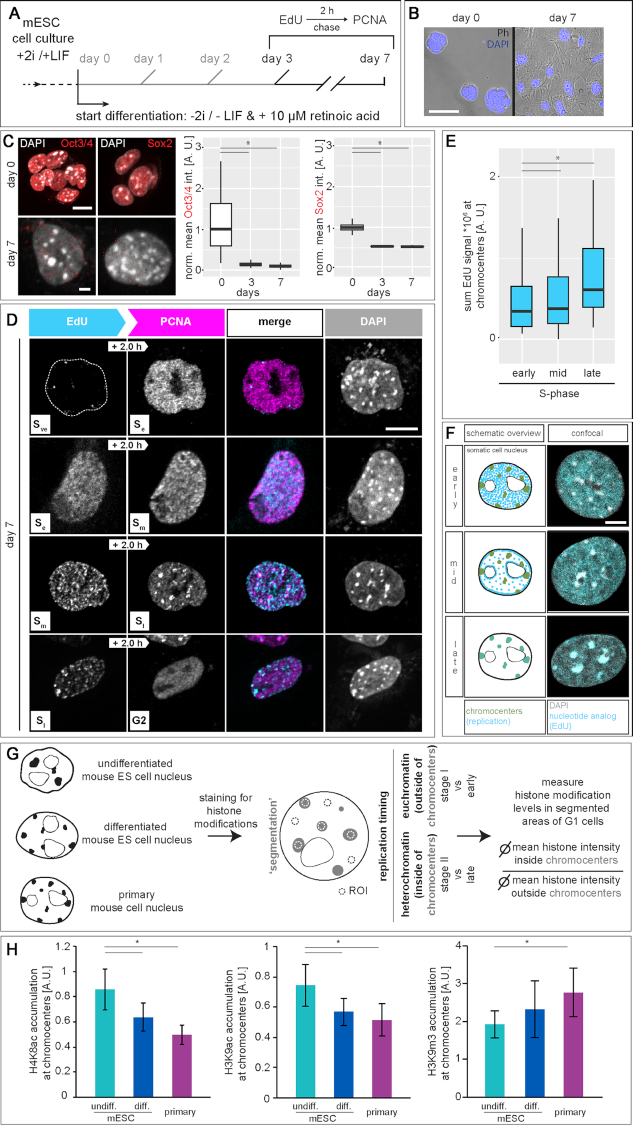
DNA replication dynamics in differentiated mouse embryonic stem cells. (**A**) Experimental setup of pulse-chase experiments in differentiated mouse J1 ES cells. Naïve pluripotent mES cells were cultured in 2i (two inhibitors (PD032591 and CHIR99021)) and LIF (leukemia inhibitory factor) containing medium. On day 0 of the differentiation, cells were seeded in 2i and LIF deficient medium containing retinoic acid. Cells were pulse labeled with EdU, chased with thymidine for 2 h and fixed at day 0 and from day 3 to day 7 of the differentiation. (**B**) Overlay of phase contrast (Ph) and DAPI channels, showing changes in cellular morphology during mES cell differentiation. (**C**) Immunofluorescent detection of the pluripotency markers Oct3/4 and Sox2 in (un)differentiated mES cells. Representative spinning disk confocal images of *in situ* stainings were imaged and the mean value of the fluorescence signal was plotted as a ratio to the undifferentiated (day 0) cells. (**D**) Representative spinning disk confocal images of pulse chased mES cells revealed replication timing switch to late replicating chromocenters at day 7 of differentiation. Very early (*S*_ve_) to early S-phase (*S*_e_), early to mid (*S*_m_), mid to late (*S*_l_) and late S-phase to G2 transitions are shown. (**E**) To analyze the replication timing switch of chromocenters in differentiated mES cells, the sum value of EdU fluorescence signal within chromocenters (masked according to DAPI channel) were measured in spinning disk confocal images of cells (from D) within early, mid and late S-phase for the EdU pulse. An increase in signal overlap of chromocenters and replication signal is observed during late S-phase in differentiated mES cells. (**F**) Schematic summary and corresponding confocal images of the three main replication patterns observed in differentiated mES cells. Replication signals are shown in cyan, pericentromeric heterochromatin in green (scheme) and replicating chromocenters (late) are marked in dark cyan. (**G–H**) Experimental setup for the analysis of histone modification accumulation at pericentromeric heterochromatin. (Un)differentiated mES cells and primary mouse ear fibroblasts were pulsed with EdU to identify S-phase cells and histone modifications were immunofluorescently detected. Regions of interest (ROI) were manually drawn in G1 phase cells and histone modification levels were measured. Shown are the accumulations of H4K8ac, H3K9ac and H3K9m3 at chromocenters (ratio of mean histone modification values at chromocenters and mean histone modification values in the nucleoplasm ± StDev). Scale bars = 5 μm. *P < 0.05. All experiments were done in at least two independent biological replicates. All boxes and whiskers represent 25–75 percentiles and 1.5 times the IQD (inter-quartile distance), respectively and the center line depicts the median (Supplementary Figure S7). Detailed statistics are summarized in Supplementary Table S8. Dotted lines represent cell contours.

In somatic cells, S-phase progression follows chromatin compaction and is commonly subdivided into early (*S*_e_), mid (*S*_m_) and late (*S*_l_) when euchromatin, facultative and constitutive heterochromatin, respectively, are replicated (1,7,14). Since we observed replication of mES cell chromocenters within the first half of S-phase, we aimed to clarify if and when during mES cell differentiation, replication timing of chromocenters switches to late replication. Therefore, we differentiated mES cells in the absence of 2i and LIF and in the presence of retinoic acid (RA) and performed pulse-chase labeling from day 3 to day 7 of differentiation (Figure 2A). During differentiation, mES cell morphology changed from round and compact 3D colonies (day 0) to more flat and spread out cells growing in a monolayer (day 7, Figure 2B). Concomitantly, levels of the pluripotency markers Oct4 and Sox2 decreased dramatically already after 3 days of differentiation and were almost undetectable after day 7 of differentiation (Figure 2C and Supplementary Table S8). All together, these results indicated loss of the stem cell phenotype and pluripotency markers that are associated with exit from pluripotency and cellular differentiation.

At day 7 of differentiation, the beginning of S-phase was marked by very few replication foci (very early S-phase, *S*_ve_), followed by a homogeneous distribution of replication foci throughout the nucleus (*S*_e_, Figure 2D). Interestingly, and in contrast to undifferentiated mES cells (Figure 1), replication signals were next observed at the nucle(ol)ar periphery (*S*_m_). Importantly, replication of chromocenters took place at the end of S-phase (*S*_l_) and was followed by transition to G2 phase. Colocalization analyses between DAPI intense nuclear regions and EdU signals showed increased overlaps of the two signals in cells showing a late S-phase pattern, further confirming the switch to late replicating chromocenters in differentiated mES cells (Figure 2E and Supplementary Table S8). In summary, the observed spatio-temporal order of replication pattern in differentiated mES cells reflects the subdivision into *S*_e_, *S*_m_ and *S*_l_ known from somatic cells (Figure 2F).

### Replication timing of constitutive heterochromatin depends on histone acetylation levels

Chromocenters are marked by the trimethylation of histone H3 at lysine 9 (H3K9m3, (47)), by histone hypoacetylation (71), exhibit increased levels of DNA methylation (72) and are bound by specific heterochromatin proteins (73). Previous studies showed that cell types of a different origin or developmental status have a different organization of pericentromeric heterochromatin (74–76). In view of this and since a more open chromatin state was proposed to facilitate early replication onset, we characterized pericentromeric heterochromatin clusters in DAPI stained mES cells and compared them to chromocenters of differentiated mES cells and primary mouse fibroblasts (Supplementary Figure S14A and Supplementary Table S9). Average numbers of chromocenters doubled over cell differentiation (Supplementary Figure S14B and Supplementary Table S9), reflecting differences in clustering of the pericentromeric regions from multiple chromosomes. Cooperatively with the increase in chromocenter numbers, we observed a decrease in the volume of individual chromocenters in differentiated cells (Supplementary Figure S14C and Supplementary Table S9). In line with this, analysis of the compaction state of the chromocenter clusters in pluripotent cells showed a more decompacted chromatin (Supplementary Figure S14D and Supplementary Table S9) and a more irregular shape (Supplementary Figure S14E and Supplementary Table S9). Accordingly, super-resolved chromatin mobility assays demonstrated a more dynamic chromatin and less defined domain structures in mES cells (77). In addition, chromatin associated proteins were found to be more mobile in pluripotent cells (78,79). It is considered that such an open conformation represents a necessary prerequisite of pluripotent cells to remain responsive to the changes that occur during differentiation (80). Additionally, the differences in clustering of chromocenters reflect the chromatin reorganization that occurs during differentiation and development (73,81). The observed differences in heterochromatin morphology, volume, clustering and compaction may provide a mechanistic basis for the observed changes in the DNA replication program of mES cells. However, a general decompaction at the scale measured via DAPI staining of DNA may not be sufficient to promote early replication of pericentromeric heterochromatin. Additionally, maintenance of late replication timing of pericentromeric heterochromatin in somatic cells was shown to depend on histone hypoacetylation (32). We, therefore, compared histone acetylation levels of chromocenters in (un)differentiated and primary mouse fibroblasts (Figure 2G) and found higher H3K9ac and H4K8ac accumulation at chromocenters in undifferentiated mES cells (Figure 2H and Supplementary Table S8). Heterochromatin acetylation levels decreased significantly during differentiation, concomitantly with the switch of replication timing of pericentromeric heterochromatin from relatively early (stage II) to late replication (S_l_). Since H3K9ac and H3K9m3, a marker for constitutive heterochromatin, are found in a mutually exclusive way in cells, we also analyzed accumulation of the latter at chromocenters. In line with the increased acetylation levels, we found less H3K9m3 accumulation in pluripotent stem cells and an increase in H3 lysine 9 trimethylation in differentiated cells (Figure 2H and Supplementary Table S8). This result is in line with genome wide studies as well as single cell microscopy analysis showing that histone acetylation is an important regulator of the replication timing of DNA. While genomic loci with increased histone acetylation levels and high accessibility tend to replicate early during S-phase, loss of histone acetylation leads to replication timing switch to late S-phase (32–34,82–89). Taken together, mid S-phase replication of pericentromeric heterochromatin in pluripotent stem cells is changed to late replication in differentiated mES cells and this switch in replication timing is likely dependent on histone hypoacetylation and chromatin compaction occurring during cell differentiation.

**Figure 3.**
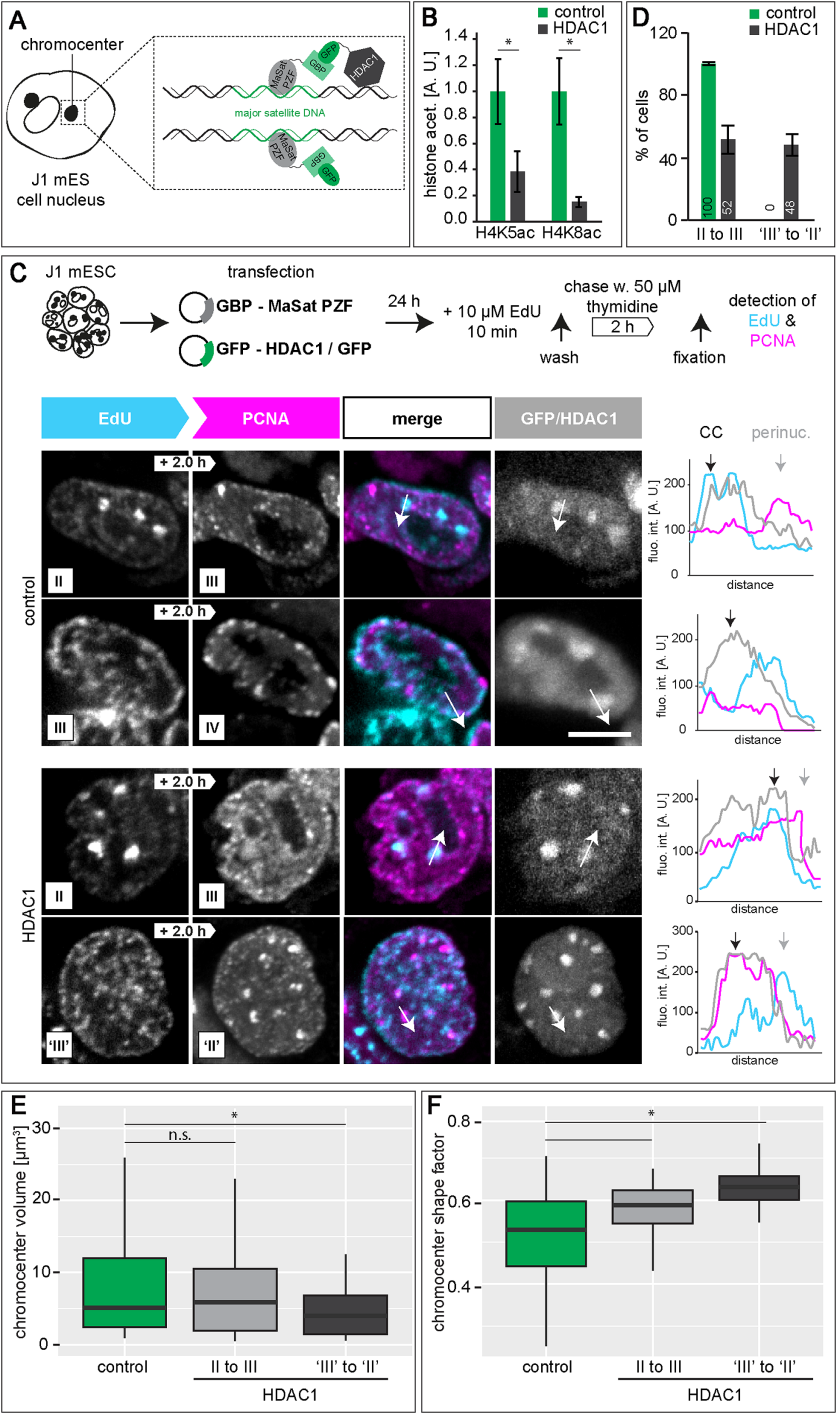
HDAC1 deacetylase targeting to chromocenters in mouse embryonic stem cells. (**A**) Schematic representation of the targeting strategy to recruit HDAC1 to chromocenters. In a targeted state, the fusion protein composed of the major satellite binding MaSat PZF (MaSat) and GFP binding protein (GBP) recruits the GFP-HDAC1 fusion protein to chromocenters via binding of the GBP to GFP. In the control experiments, only GFP was recruited to chromocenters. (**B**) Histone acetylation (H4K5ac and H4K8ac) levels in (HDAC1) targeted and GFP control cells (mean ± StDev). (**C**) 24 h after double transfection of J1 mES cells, cells were pulse chased as described in Figure 1. Representative spinning disk confocal images from GFP control cells showing S-phase substage transitions from stage II to III and from stage III to IV and from HDAC1 targeted cells showing transitions form stage II to III and from stage ‘III’ to ‘II’. Line profiles represent fluorescence intensities along the arrow marked in the images. CC = chromocenter, perinuc. = perinucle(ol)ar. **(D)** Percentages of cells representing stage II to III and stage ‘III’ to ‘II’ transitions in control and targeted cells (mean ± StDev). (**E-F**) Chromocenter volume (**E**) and chromocenter shape factor (**F**) in control cells and cells showing stage II to III and stage ‘III’ to ‘II’ transitions in HDAC1 targeted cells. *P < 0.05 and n.s. = non-significant. Detailed statistics are summarized in Supplementary Table S8. Scale bar = 5 μm.

To validate the causality between the observed histone hyperacetylation and early/mid replication of pericentromeric heterochromatin in mES cells, we targeted a GFP tagged histone deacetylase to chromocenters in mES cells (90). Specific chromocentric targeting was achieved via co-transfection of GFP tagged HDAC1 and a GFP binding protein (GBP) tagged MaSat polydactyl zinc finger (PZF) protein (GBP-MaSat). MaSat PZF specifically binds to major satellite repeat DNA (chromocenters) and its GBP domain interacts with GFP-HDAC1, thereby recruiting the deacetylase to chromocenters (Figure 3A). As a control, we targeted GFP to chromocenters. With this setup, we first analyzed histone acetylation levels in transfected cells, and found decreased H4K5ac and H4K8ac levels in HDAC1 targeted cells compared to control GFP cells (Figure 3B and Supplementary Table S8). To analyze spatio-temporal S-phase progression in transfected cells with altered chromocentric histone acetylation levels, we performed pulse chase experiments. Therefore, 24 h after double transfection, cells were labeled with EdU, chased for 2 h and fixed. EdU and PCNA were (immuno)fluorescently detected and DNA replication progression was investigated by pattern order analysis as before. In GFP control targeted cells, we observed spatio-temporal DNA replication progression as previously described. Pericentromeric heterochromatin was replicated before perinucle(ol)ar chromatin and finally replication signals were observed as bigger replication foci throughout the nucleus (stage II–stage III–stage IV). In HDAC1 targeted cells, however, we observed two populations of cells. First, we found cells following the classical mES cell replication progression where S-phase moves from chromocenters to the perinucle(ol)ar border. Second, cells showed replication of the perinucle(ol)ar chromatin followed by replication signals in normally earlier replicating chromocenters (stage ‘III’ to stage ‘II’). This indicates an at least partial delayed chromocenter replication upon histone acetylation level decrease (Figure 3C-D and Supplementary Table S8). Quantitative analysis revealed almost equal distributions between the two substage orders in HDAC1 targeted cells, while control cells only showed stage II to stage III transitions (Figure 3D and Supplementary Table S8). Interestingly, after HDAC1 targeting we observed massive rearrangements of pericentromeric heterochromatin. GFP control cells exhibited few but large chromocenters, while 24 h of histone deacetylase targeting resulted in an increased number of constitutive heterochromatin clusters with decreased size. Analyzing chromocenter characteristics, we measured similar volumes and shape factors for control cells than in untransfected mES cells (Figure 3D-E, Supplementary Figure S14C, S14E and Supplementary Tables S8 and S9). HDAC1 targeted cells were subdivided according to their S-phase progression pattern (II to III or ‘III’ to ‘II’). We detected significant changes in chromocenter volumes in stage ‘III’ to ‘II’ cells compared to control and stage II to III cells. Additionally, shape factors were also significantly different in targeted cells showing a replication pattern switch (Figure 3E-F and Supplementary Table S8). In conclusion, we show that a targeted histone deacetylation of mES chromocenters leads to a switch in replication timing of pericentromeric heterochromatin to later in S-phase, mimicking our findings in differentiated mES cells (Figure 2D).

### Replication timing of (sub-)chromosomal elements in mouse embryonic stem cells

**Figure 4.**
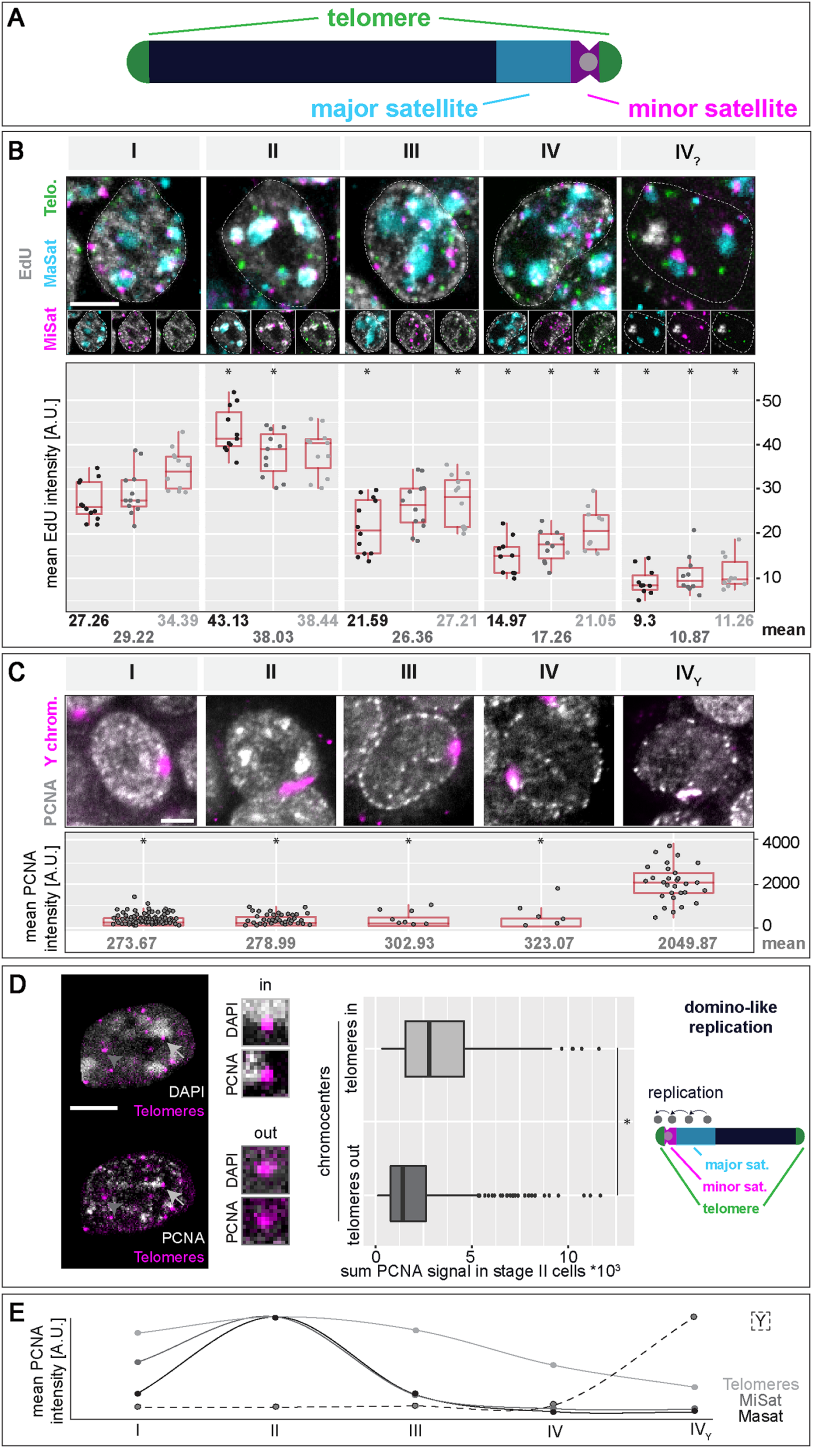
Replication timing of (sub)-chromosomal structures in mouse embryonic stem cells. (**A**) Schematic representation of a mouse acrocentric chromosome. Centromeric satellite regions (MiSat) and flanking pericentromeric DNA (MaSat) are depicted in magenta and cyan, respectively and telomeres are shown in green. (**B**) Tandem repeat elements were co-visualized with EdU (labeling of nascent DNA, grey) in mES cell interphase nuclei by triple FISH hybridization. Cells were classified into S-phase stages I to IV_?_ according to their EdU pattern, mean EdU intensities within the marked elements were measured as described in Supplementary Figure S3 and plotted. Mean values are indicated below each plot. (**C**) Analysis of Y chromosome FISH in combination with PCNA staining was performed as in (B). (**D**) Analysis of telomere replication timing in J1 mES cells co-stained for PCNA and telomeres. Chromocenters of S-phase stage II cells were segmented according to the DAPI staining, and sum values of PCNA fluorescent intensity were measured within segmented telomeres located in close proximity to chromocenters (light grey arrows, ‘telomeres in chromocenters’) and within telomeres located on the long arm of the chromosome (not in proximity of chromocenters, dark grey arrow heads, ‘telomeres out of chromocenters’). Replication of chromocenter near telomeres within S-phase stage II hints towards a domino-like replication model with a sequential order of replication of adjacent chromosomal regions (MaSat/MiSat to telomeres on the short chromosome arm). (**E**) Summary of the replication timing of tandem repeat elements and the Y chromosome in mES cells. (Peri)centromeric DNA regions (MaSat and MiSat) are mainly replicated within the first half of S-phase (stage II), telomeres are replicated throughout S-phase and the Y chromosome marks the end of S-phase (stage Y). Dotted lines represent cell contours. Boxplots are as in Figure 2 and Supplementary Figure S7. Scale bar = 5 μm. Detailed statistics are summarized in Supplementary Table S10. *P < 0.05 (calculated among each elements against the respective stage I value (B) or against stage Y in C).

In human and mouse genomes, only minor parts (1.2–1.4%, respectively (91,92)) are protein-coding sequences, while the major portion is composed of non-coding DNA, including interspersed and tandem repeat sequences. There is growing evidence that the latter is more than ‘junk’ DNA, since it is thought to be involved in the establishment of distinct eu- and heterochromatin compartments (93–95). Additionally, genome function might not only be influenced by epigenetic factors, but also by the spatial organization of the genome within the cell nucleus (96). Hence, we analyzed the nuclear distribution and the replication timing of several sub-chromosomal tandem repeat elements in mouse ES cells. Due to their repetitive nature, repeat elements are normally under-represented in genome-wide sequencing studies. We, therefore, opted for a single cell microscopic approach, where we combined DNA replication visualization and marking of three major chromosomal tandem repeats (Figure 4A and Supplementary Figure S15A) via FISH (Repli-FISH, (55)).

To analyze the replication timing of the repeat elements, we quantified the overlap of the replisome factor PCNA or nascent DNA labeled by EdU with individually segmented repeat sequence specific FISH signals within 3D mES cell interphase nuclei. The specificity of the FISH probes for (peri)centromeric DNA (major and minor satellites, respectively) and telomeres was validated by their location on mitotic chromosomes (Supplementary Figure S15B and (55)). Major satellite pericentromeric repeats were visualized as large clusters of DNA, co-localizing with chromocenters, marked by bright DAPI counterstaining, and mainly associated with the nuclear periphery and nucleoli in the 3D nucleus (Supplementary Figures S16A, S17A and Supplementary Table S10). Strongest overlap with PCNA or EdU was observed in S-phase stage II (Figure 4B, Supplementary Figures S16A, S15A, S18A–B and Supplementary Table S10) in line with our results above from *in vivo* and pulse-chase experiments (Figure 1). Minor satellite centromeric repeat signals were seen as small focal structures located in close proximity to chromocenters (Figure 4B, Supplementary Figures S16B, S17B, S18B–C and Supplementary Table S10). Akin to major satellite repeats, centromeric DNA of mES cells was found to also replicate preferentially in early/mid S-phase (stage II). Centromeres of *Drosophila* as well as yeast, were found to replicate only early in S-phase suggesting that early replication timing is a conserved feature of centromeres (97,98). In mouse fibroblasts, however, centromere replication was also reported to occur throughout S-phase (99,100). Given their physical location along chromosomes and their 3D organization in interphase nuclei (Supplementary Figure S15A and (72)), it is likely that centromeric regions are also less compacted in mES cells so that partial overlap of the replication timing profiles of these two structures can be expected. Furthermore, and as shown in somatic cells (99), centromeric repeats likely replicate just before or after the directly adjacent pericentromeric chromosomal domains. In the case of telomeres, the FISH signals distributed as smaller individual foci throughout the cell nucleus (Figure 4B, Supplementary Figures S16C, S17C, S18A, S18C and Supplementary Table S10). Overlap of replication and telomere signals was observed at all S-phase stages, with an increase during stage II.

Although our previous observations (Figure 1A and C and Supplementary Figure S11) suggest a difference in chromatin clustering in S-phase stage III and IV, we could not identify any specific tandem repeat sequence underlying these stages. Since none of the RFi features quantitatively analyzed (Supplementary Figure S12) showed significant differences between these two stages, we consider this part of S-phase as one substage and will refer to it, hereafter, as stage III.

Since the J1 mES cells were derived from the inner cell mass of a male blastocyst (45), we additionally analyzed the replication timing of the Y chromosome (Supplementary Figure S15C). The strong accumulation of replication signals observed in stage IV_?_ of S-phase showed a significant overlap with FISH signals specific for the Y chromosome (Figure 4C, Supplementary Figures S16D, S17D, S18A–C and Supplementary Table S10). We, therefore, consider S-phase stage IV_?_ as the male specific S-phase stage where the Y chromosome is replicated and will refer to it, hereafter, as stage Y.

Since we measured an increase in PCNA-telomere signal overlap in S-phase stage II cells, we analyzed if telomere replication timing was dependent on their 1D proximity to pericentromeric heterochromatin, which is replicated during this stage of S-phase. We, therefore, segmented telomeres in stage II S-phase cells, grouped them according to their location with regards to chromocenters and measured PCNA signal intensities within the segmented regions. Telomeres on the short arm of acrocentric chromosomes (telomeres in chromocenters) showed higher PCNA signals in stage II cells than telomeres capping the long chromosomal arms (telomeres out of chromocenters, Figure 4D and Supplementary Table S10). This observation underlines a potential domino-like replication where the activation of origins of replication takes place in a next in-line manner, thereby spreading from the (peri-)centromeric repeats towards the telomeres located on the short arm of the chromosome (99). This is in line with earlier studies relating the replication timing of telomeres to nuclear position, with telomeres positioned towards the nuclear interior replicating earlier than the ones associated with the nuclear periphery (101).

In summary, we characterized the replication timing of the three main classes of tandem repeat sequences and of the Y chromosome via a single cell microscopic approach. (Peri)centromeric DNA was mainly replicated during stage II of S-phase, while telomeres were replicated over the complete duration of S-phase. Replication of the Y chromosome marked the end of S-phase and corresponded to the strong accumulation of replication signals in stage Y (Figure 4E).

### Synchronous replication of the Y chromosome marks the end of S-phase in pluripotent and differentiated cells

The Repli-FISH method allowed us to determine that the prominent structure that is replicated at the end of S-phase in stage Y in male J1 mES cells is the Y chromosome (Figure 4C). In female cells, the inactive X chromosome (Xi) replicates in a highly synchronous manner and, in contrast to the active homologue, within a short time interval during early-mid S-phase (31). Similarly, we did not detect any major replication signal at the Y chromosome in any of the other four S-phase stages and, therefore, conclude that the Y chromosome is synchronously replicated within this short period of S-phase stage Y (Supplementary Figure S8D). To further determine if this *en bloc* replication of the Y chromosome at the end of S-phase is a characteristic of pluripotent mES cells or a general feature of male cells, we analyzed the PCNA and Y chromosome hybridization signal overlap in replicating male mouse embryonic fibroblasts (MEF W8). In addition to the three well characterized S-phase patterns known from somatic cells (*S*_e_, *S*_m_ and *S*_l_), MEF cells showed a fourth pattern with a clear accumulation of replication sites at the Y chromosome (Supplementary Figure S19A and Supplementary Table S10). Pulse chase experiments in differentiated mES cells and mouse embryonic fibroblasts showed a similar pattern after chromocenter replication during late S-phase (S_l_) and before transition to G2 phase (Supplementary Figure S19B). The Y chromosome of male cells, one of the smallest chromosomes in mice (∼92 Mb), is mostly studied in the context of evolution, clinics and forensics (102) and mainly believed to consist of non-functional DNA (103). At the genomic level, the Y chromosome is marked by a very low gene density (1.7 genes/Mb for mice, (74,104,105)) of which only a fraction appears to be potentially protein coding in humans and that are mostly required for testis development and sex determination, or have a X encoded paralog (105,106). Additionally, the Y chromosome is composed of large heterochromatic DNA blocks (107,108). Therefore, gene expression and transcriptional activity on the Y chromosome are low and a connection between low gene density and transcriptional activity could explain the late replication timing of the male sex chromosome. Accordingly, a correlation between gene expression and replication timing was demonstrated in *Drosophila* (109) and, similarly, between replication timing, GC content, gene density and transcriptional activity in human cells (68,110–112). Besides this, the Y chromosome is frequently lost in most male cell lines during prolonged cell culture and, thus, neglected in most studies. Our microscopic data provide evidence for a synchronous replication of the Y chromosome marking the end of S-phase independently of the pluripotency state as a general characteristic of mouse male cells. Interestingly, mid S-phase replication of the silenced copy of the X chromosome in somatic female cells, also occurs in a synchronous manner (31), highlighting a common replication mode for transcriptionally inactive chromosomes. This replication mechanism bears resemblance to observations in early *Drosophila* and *Xenopus* embryos, where genome duplication is performed in extraordinarily short time frames and in the complete absence of transcription (35,113–116).

### Cell cycle and S-phase stage kinetics in mouse embryonic stem cells

**Figure 5.**
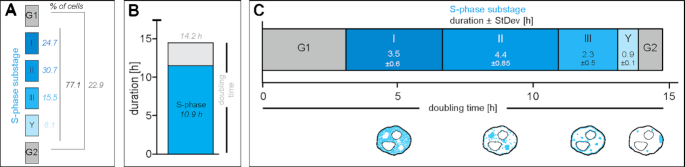
Cell cycle characteristics of mouse embryonic stem cells. (**A**) The cell cycle distribution within an asynchronous mES cell population was analyzed by labeling the cells with EdU and counting the number of S-phase cells (EdU positive). Around 77% of the cells are in S-phase and additionally, the percentage of cells within S-phase substages (I–Y) is detailed. (**B**) Growth curve analysis over 5 days revealed a population doubling time of around 14 h for mouse J1 ES cells. Together with the percentage of replicating cells from (A), an approximate S-phase duration for mES cells of about 10.9 h was calculated. (**C**) From the fraction of cells within every S-phase substage (I–Y, A) and the total S-phase duration (B), approximate durations of the individual substages were calculated (mean ± StDev). All experiments were done in at least three independent biological replicates. Detailed statistics are summarized in Supplementary Table S11.

Embryonic stem cells have the unique characteristics to replicate indefinitely in cell culture while maintaining their self-renewal capacity and to differentiate into cells of all three germ layers. Their cell cycle also differs significantly from somatic cell types (117) and upon differentiation, cell cycle dynamics undergo massive reorganization (118–120). To study DNA replication kinetics in mouse J1 ES cells, we analyzed total S-phase and S-phase substage lengths, as well as the population doubling time in asynchronously growing J1 mES cells. Cell cycle distribution was analyzed by counting S-phase and non S-phase cells (G1, G2 or mitotic cells) in EdU pulsed cell populations. On average, around 77% of J1 mES cells were in S-phase at any given time, while 23% were not actively replicating DNA (Figure 5A and Supplementary Table S11). Furthermore, around 31% of S-phase cells were in stage II, replicating pericentromeric heterochromatin, while 25% showed the stage I pattern. The other stages were represented by 20% of the cells. Cell proliferation rates were calculated over five consecutive days and J1 mES cells exhibited a doubling time of around 14 h (Figure 5B), which is in agreement with doubling times measured for other mouse ES cell lines (121). Together with the percentage of cells in S-phase (Figure 5A and Supplementary Table S11), an average S-phase duration of almost 11 h was calculated (Figure 5B). Similarly, S-phase substage durations were derived (Figure 5C). During almost two-thirds of S-phase length, mES cells were present in stage I or II, replicating euchromatic and constitutive heterochromatin. Stage III and Y accounted for only 1/3 of S-phase length. These results are comparable to the S-phase substage durations we measured via live cell microscopy (Supplementary Figure S8D). All in all, J1 mES cells exhibit a similar S-phase duration to somatic cells, however, their doubling time is significantly shorter (28,120). This observation is in line with previous reports that stem cells have shorter gap phases and are devoid of a G1-S transition regulation (118). Strikingly, although mES cells exhibit a different temporal organization of DNA replication, around 72% of S-phase are necessary to replicate active euchromatic chromatin and silenced constitutive heterochromatin (stage I and II), which is similar to the time mouse myoblasts and embryonic fibroblasts spend in *S*_e_ and *S*_l_ (20)

### Superresolved replication nanofoci in mouse embryonic stem cells are activated in spatial clusters similar to somatic cells in number but larger in volume

**Figure 6.**
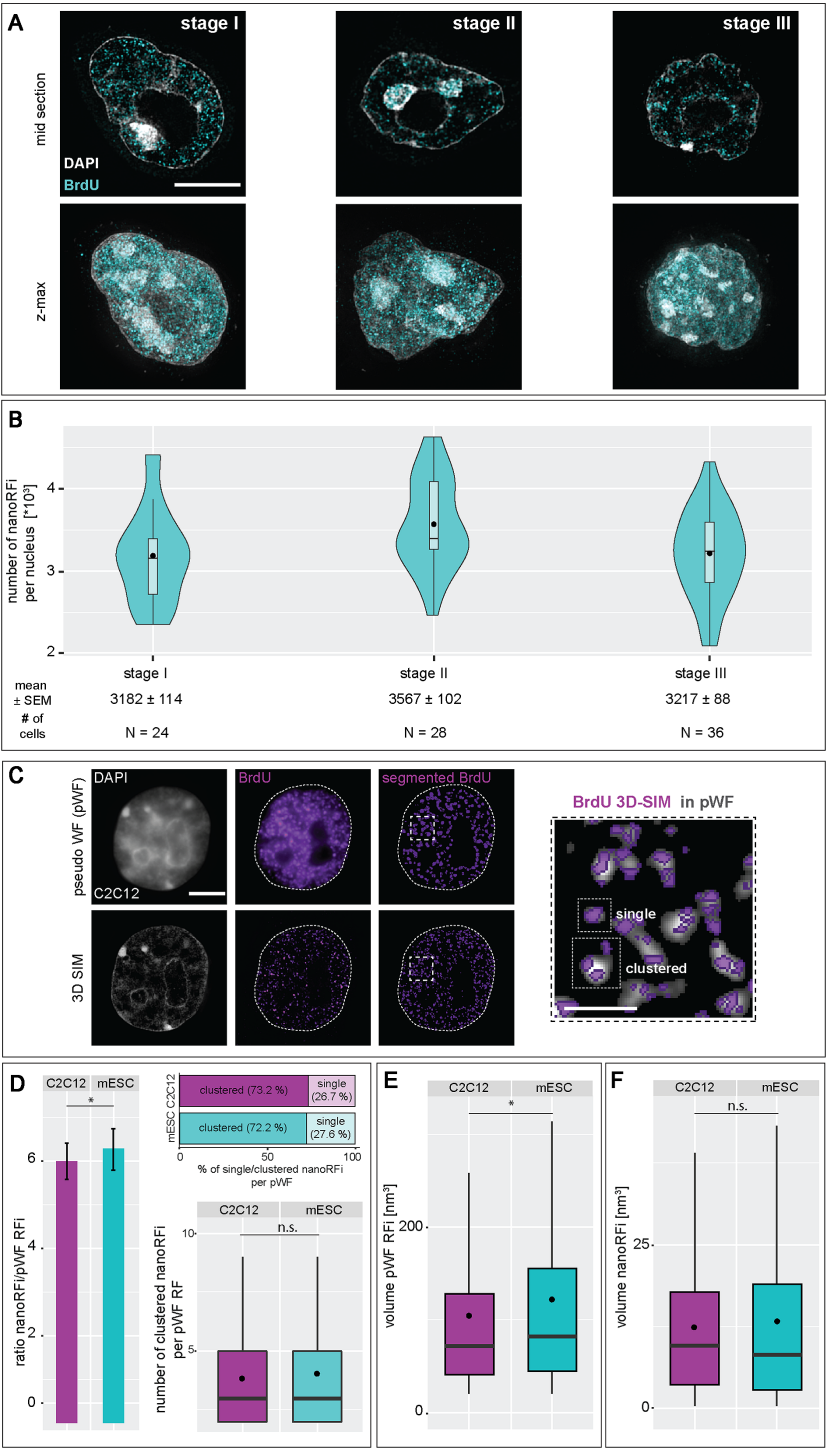
3D quantification and analysis of replication foci throughout S-phase in mouse embryonic stem cells. (**A**) Mid sections and maximum intensity *z*-projections (*z*-max) of 3D structured illumination microscopy (3D-SIM) images of mouse ES cells representative of the first three S-phase patterns (I–III) are shown. **(B)** Numbers (mean ± SEM) of nano replication foci (nanoRFi) quantified as described in Supplementary Figure S4 are plotted separately for each of the three S-phase patterns. N indicates the number of cells analyzed. (**C**) At lower optical resolution, replication signals appear as larger foci (pseudo wide-field (pWF) foci). These can be resolved to a number of smaller foci when imaged by super-resolution microscopy. Shown are representative pWF (upper row) and the respective 3D SIM images (lower row) of the cell nucleus of a mouse myoblast, the unsegmented (middle column) and segmented (right column) BrdU replication signals. The pWF replication foci were segmented as described in Supplementary Figure S5 and used to demarcate a distinct volume of DNA in which the number of nano replication foci (nanoRFi) was quantified (magnified inset). nanoRFi are considered as ‘clustered’ if one pWF focus contains more than one nanoRFi. (**D**) Results of cluster analysis of nanoRFi within the distinct volume of a pWF replication focus are shown. RFi ratios from super-resolution versus pseudo-widefield microscopy (barplot ± Stdev) and analysis of the number of clustered nanoRFi in individually segmented pWF foci (boxplot) in early S-phase mouse ES and myoblast (C2C12) cells are shown. Percentages of single and clustered nanoRFi are depicted. (**E-F**) Volumes of the segmented pWF (**E**) and 3D-SIM (**F**) nano replication foci (nanoRFi) are shown. Detailed statistics are summarized in Supplementary Table S12. Boxplots are as in Figure 2. * *P* < 0.05 and n.s. = non-significant. Black dots within violin/box plots represent mean values. Scale bar = 5 and 2.5 μm for main graphs and magnified regions, respectively. Brightness and contrast of 3D-SIM images were adjusted for every image depicted. Dotted lines represent cell contours.

Advanced optical microscopy techniques allow imaging beyond the resolution limit inherent to light microscopy. By using multicolor 3D structured illumination microscopy (3D-SIM), it was recently shown that individual replication foci are resolved down to single replicons and, to some extent, even to individual replication forks (28). Chromatin organization and chromosomal interactions are known to change during embryonic stem cell differentiation (122). Moreover, differences in the organization of the underlying chromatin fiber, e.g. a more open chromatin conformation, may have an impact on the organization and activation of replication origins, and, in turn, on the regulation of replication timing of different chromatin classes. We therefore analyzed replication foci at the different S-phase substages in mouse ES and mouse myoblast (C2C12) cells imaged with 3D-SIM (nano replication foci or nanoRFi). To quantify the number of super-resolved nanoRFi, mES cells were labeled with 5-bromo-2′-deoxyuridine (BrdU), the nucleotide analog was immunofluorescently detected and S-phase cells were imaged. Replication foci were thresholded, masked and counted as described (53). Comparable numbers of nano replication foci for cells from the first three S-phase substages were obtained, with on average 3320 ± 60 (mean ± SEM) nanoRFi at any given time during S-phase (Figure 6A, B and Supplementary Table S12). Since the number of nano replication foci dropped dramatically at the end of S-phase (stage Y: 1881 ± 214), we excluded this stage from all subsequent calculations. A similar trend was found in the number of RFi measured from confocal microscopy images (Supplementary Figure S12). NanoRFi measurements in somatic cells revealed a decreased number of super-resolved foci in late S-phase cells, when highly compacted constitutive heterochromatin is replicated (28). Interestingly, we detected comparable numbers of nanoRFi in mES stage II S-phase cells and all other S-phase stages. This discrepancy could be explained by the more decompacted heterochromatin in mES cells (Supplementary Figure S14), facilitating individual foci segmentation. Additionally, this result supports the idea that differences in chromatin organization influence DNA replication (78,79,123).

Since origins of replication are proposed to be located at loop anchors (26) and based on the hypothesis that a different chromatin compaction and (loop) organization in mES cells could result in a spatially different organization of nano replication foci and replicons, we compared the numbers of nanoRFi in a given volume within the cell nucleus of mouse myoblast and embryonic stem cells. The 3D-SIM system simultaneously allows the generation of reconstructed super-resolved 3D image sets and the corresponding (pseudo)wide-field (pWF) images (Figure 6C). This allowed us a direct comparison of the total number of RFi per cell from different imaging resolution conditions from the same cells. We, therefore, segmented 3D-SIM nanoRFi and pWF RFi from early and stage I C2C12 and mES cells, respectively, and calculated the population ratio of RFi. The two cell types showed a similar ratio indicating that every RFi imaged at conventional light microscopy resolution corresponds on average to 5.9 and 6.5 super-resolved nanoRFi for C2C12 and mES cells, respectively (Figure 6D and Supplementary Table S12). In addition to the ratiometric approach, we correlatively analyzed the connection of pWF and nanoRFi by determining the number of super-resolved RFi per underlying pWF focus in both cell types. Hence, we first aimed to determine the amount of clustered (> 1 nanoRFi per pWF) versus single (exactly 1 nanoRFi per pWF) nanoRFi within the given volume of the pseudo-widefield focus. Interestingly, in both cell lines, around 72–73% of the nanoRFi formed clusters, while 26–27% of the pWF contained only one nanoRFi. While the above mentioned ratiometric approach includes all detected and segmented pWF, independent of the number of underlying nanoRFi, the correlative analysis takes into consideration only pWF foci containing more than one nanoRFi (clustered nanoRFi). These pWF replication foci comprised clusters of on average 3.82 and 4.02 nanoRFi for C2C12 and mES cells, respectively (Figure 6D and Supplementary Table S12). Notably, stem cell pWF RFi are significantly larger than myoblast pWF foci while no difference in size of nanoRFi could be detected, suggesting that they correspond to conserved (elementary) structural units of the mammalian genome (28) (Figure 6E and F and Supplementary Table S12). In summary, the observed difference of mES cell replicons from those of mouse myoblast cells, suggests that the underlying chromatin and loop organization is different in mES cells (122,124) and that this difference is reflected in the organization and dynamics of DNA replication (78,79,123). Chromatin loop structures are considered distinct units in the complex hierarchy of genome organization within the cell nucleus. At this level, individual loops may harbor the DNA elements that act as templates or regulators in different molecular processes (reviewed in (27)). The most prominent examples are enhancer elements and promoters of genes that coordinate and regulate transcription via long ranging cis-interactions (94,125,126). Notably, in somatic cells, measured sizes for chromatin loops, replication forks and also nano-repair foci are highly consistent and rely on the same structural DNA unit of about 90 kb (28,60,127). Coherently, a replicon consisting of bidirectional replication forks, duplicates a DNA segment the size of a pair of loops, i.e. around 180 kb (28). Thus, differences in loop conformation and (local) chromatin density are potent features that can modulate interactions between genetic elements that are required for DNA-dependent metabolic processes such as DNA replication. In this regard, it is appealing to propose that if replication origins are defined by loop anchoring sites, then organization of replicons will be directly linked to loop structures.

### Molecular characteristics of the replicon in mouse embryonic stem cells reveal increased numbers of activated origin and unidirectional forks

**Figure 7.**
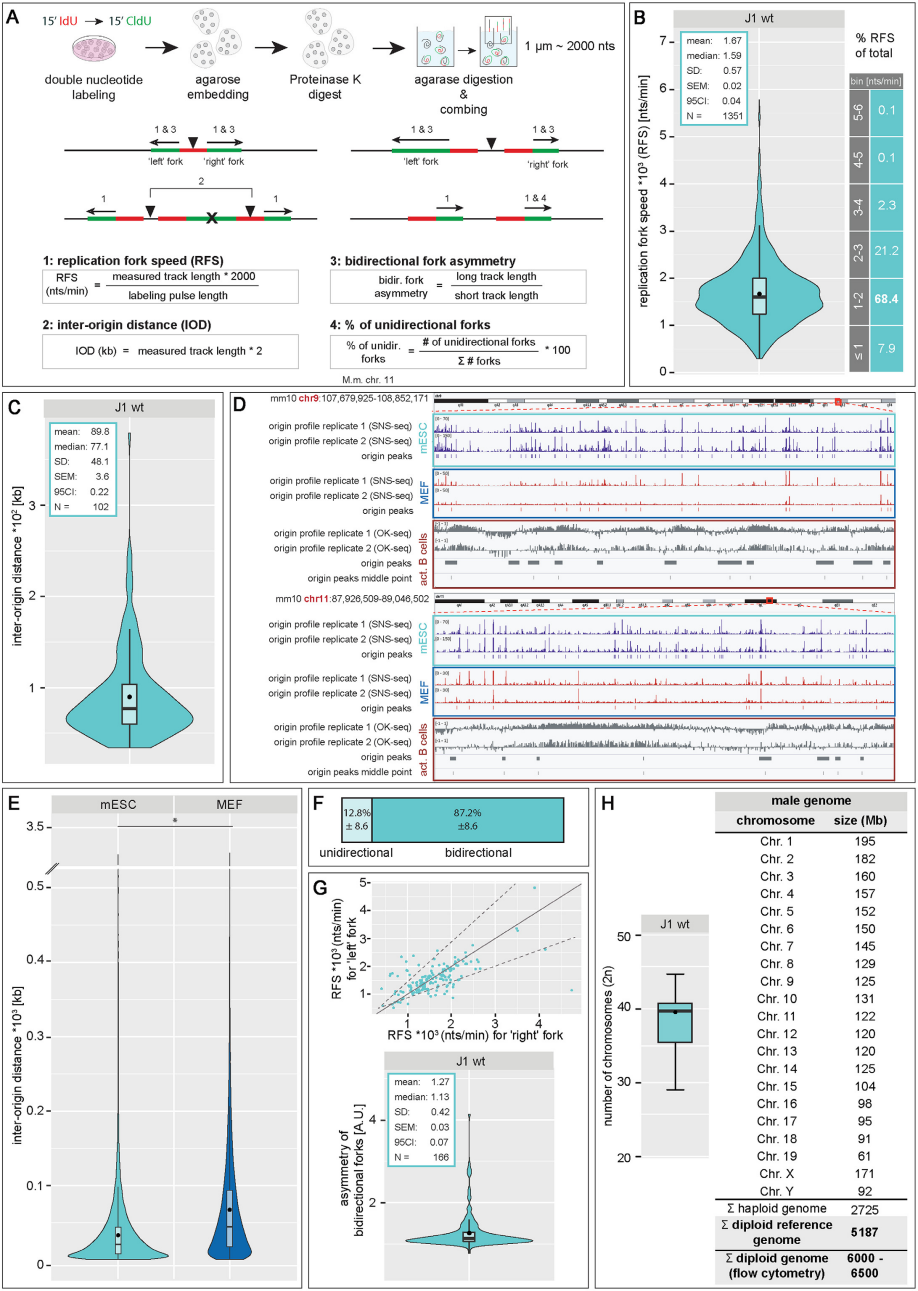
DNA replication fiber and genome size analysis in mouse embryonic stem cells. (**A**) Schematic outline of the experimental setup for DNA fiber analysis. mES cells were sequentially labeled with IdU and CldU for 15 min, harvested and embedded in agarose. After a proteinase K digestion step, agarose was digested and high molecular weight naked DNA was stretched on silanized glass coverslips. Nucleotide analogs and single stranded DNA (ssDNA) were immunofluorescently detected. (**B–E**) The length of fluorescent tracks of the second pulse (CldU) were measured (1 μm ∼ 2000 nucleotides, Supplementary Figure S6) and the mean replication fork speed (RFS, (**B**)), inter-origin distance (IOD, (**C**)), percentage of unidirectional forks (**F**) and asymmetry of bidirectional forks (**G**) were calculated as indicated in (A). Additionally, the percentages of forks within a given range of RFS are indicated in (B). For comparison of the RFS of the ‘left’ and ‘right’ fork of a bidirectional fork, RFS values were plotted in a scatterplot. The solid grey line represents the linear relation *x* = *y* and dotted lines represent thresholds allowing for a 35% (± StDev calculated for the asymmetry factor) difference between lengths of the two forks. (**D**) Visual representation of origin mapping in two arbitrarily selected regions (mouse (Mus musculus, mm10) chromosomes 9 and 11). SNS-seq origin profiles and identified origins in mES and MEF cells are shown. OK-seq origin profiles in activated (act.) B cells, called peaks along with the middle point of each peak are represented. Replication profile scale is indicated in the upper left corner. The comparison between identified and clustered origin peaks is shown in Supplementary Figure S20. (**E**) IOD distributions based on the genome-wide origin maps in mES cells and mouse embryonic fibroblast (MEF) are shown (SNS-seq). The sequencing datasets used for the analysis include two independent replicates of origin mapping for each condition. (**H**) Ploidy of J1 mES cells was determined via karyotype analysis of metaphase spreads. For genome size calculation, the sizes of individual mouse chromosomes (19 autosomes + X and Y chromosomes) were retrieved from the Genome Reference Consortium database. Additionally, genome sizes measured by flow cytometry are indicated. Boxplots/violinplots are as in Figure 2 and Supplementary Figure S7. Statistical details are depicted in the plots or summarized in Supplementary Table S13. All experiments were done in at least two independent biological replicates. Black dots within box/violin plots represent mean values. * *P* < 0.05. Scale bar = 5 μm.

Complete genome duplication once per cell cycle and in the restricted time frame of S-phase depends on two determinants: (i) the number and distribution of initiation sites (i.e. replication origins) along the genome (inter-origin distance) and (ii) the processivity rate (nucleotides/time) of replication forks (replication fork speed) emanating from these sites. With regard to the differences in the spatio-temporal replication dynamics of mouse ES cells described above and in view of the highly dynamic spacing of adjacent origins within replicon clusters in *Xenopus* and *Drosophila* development (36,37,113), we aimed to clarify if mES cells exhibit further replication related differences/adaptations compared to somatic cells. To analyze the molecular characteristics of replicons in mES cells, i.e. the replication fork (elongation) speed (RFS) and the inter-origin distance (IOD), we consecutively labeled asynchronous J1 mES cell populations with two halogenated thymidine analogs, IdU and CldU and performed molecular combing assays with isolated high molecular weight DNA that led to uniformly stretched DNA fibres (1 μm ∼ 2 kb, Figure 7A). The replication fork speed obtained in mES cells of 1.67 ± 0.02 kb/min (mean ± SEM, Figure 7B) is within the range of fork rates measured by the same single molecule DNA fiber analysis for other cell lines (28,128). This suggests that replication fork speeds are similar between pluripotent and somatic cells. On the other hand, the average inter origin distance of ∼90 kb (Figure 7C) is smaller in mES cells compared to mouse myoblasts and human cells (28). This result indicates that the organization of replicons or replicon clusters is different between mouse pluripotent and somatic cells. Although the observed differences in IOD lengths between mES and differentiated somatic cells are not as dramatic as those occurring during development of *Xenopus* embryos, they suggest that the modulation of inter-origin distances, and concomitantly the resulting replicon sizes, represent a mechanism that is similar between the two species and highlights further developmental differences of the replication timing program of murine ES cells. Of note, while the first cell divisions in the *Xenopus* zygote occur in the absence of DNA transcription, transcription initiation is observed in the pronuclei of the zygote and at the two-cell stage during mouse development (35–37,39,41,129).

To get a deeper insight in the distribution of genome-wide replication initiation events, we compared the results of our microscopic analysis with the available datasets of sequencing-based genome-wide origin mapping in mES cells (61,62), mouse embryonic fibroblast (MEF) cells (62) and activated mouse B cells (64). This reflects the origin distribution in three different stages of differentiation, from stem cells through differentiated but still reprogrammable MEF cells, to a terminally differentiated B cell line. The origin mapping in mES and MEF cells was realized using short-nascent-strand (SNS-seq) isolation and sequencing (130) and origins in B cells were mapped by Okazaki fragment sequencing (OK-seq) (131). The genome-wide origin mapping was realized using asynchronous cell populations that permit cumulative mapping of all replication origins independently of their timing of firing. A total number of 78 238 and 71 257 DNA replication origins were identified in two independent datasets using mES cells (61,62). In MEF, 34 196 DNA replication origins were identified, i.e., two times less origins than in mES cells. The two times lower number of origins led to a two times larger inter-origin distance in MEF cells in comparison to the mES cells (Figure 7D and Supplementary Figure S20). We determined average IODs of 33.6 and 69.93 kb for mES and MEF cells, respectively (Figure 7E and Supplementary Table S13). Interestingly, the IOD calculated for the somatic chromosomes was two times smaller than for the X chromosome (62 in mES and 82 kb in MEF) (Supplementary Figure S21A-C). This suggests a different spatial origin activation on the two parental homologue chromosomes leading to an apparently smaller IOD calculation than the IOD measured for a single stretched DNA molecule in the combing experiments. The decrease in origin number and increase in the IOD became even more prominent in the terminally differentiated B cell line activating around 9000 origins. The SNS-seq and OK-seq differ in the origin-mapping precision, the SNS-seq method offers a very good mapping resolution and identifies all possible initiation sites (ISs). With an average peak size of 0.5 kb in the datasets analyzed (Supplementary Figure S22A and Supplementary Table S13), most of the ISs are situated in close proximity and represent alternative origin firing patterns within a cell population. The OK-seq identifies large initiation zones (IZs) with an average resolution of 22 kb (Supplementary Figure S22A) without distinguishing single initiation sites. To equalize the resolution of the two methods for comparison purposes, all SNS-seq initiation sites were clustered in the distance of the average resolution of OK-seq origin mapping (22 kb). As a result of this operation, all origins found in mES and MEF cells were clustered in 33 765 and 23 862 border IZs, situated at an average distance of 70.8 and 100.2 kb, respectively. In contrast the 9000 IZs identified in the activated B cells were spaced at the distance of 288 kb (Supplementary Figures S20 and S22B). This result suggests a gradual decrease in origin firing and increase in origin spacing during differentiation and supports our finding of smaller inter-origin spacing in mES cells compared to somatic cells obtained from single molecule DNA combing data.

Next, we analyzed the portion of single/unidirectional forks (replication forks without counterpart/opposite direction fork) per total forks present in mES cells and observed that around 13% of all forks migrate away from the origin of replication in only one direction (Figure 7F and Supplementary Figure S6). The presence of unidirectional forks has also been found in sequencing-based genome-wide origin mapping techniques in human cells. In the latter, genome duplication relies on 4.1% to 7.3% of unidirectional forks (131). We obtained similar numbers upon extracting the amount of unidirectional forks from the DNA combing analysis datasets of Chagin *et al.* (28), where we measured 5.5 ± 1.3% of forks travelling in only one direction from the origin of replication. In addition, we also analyzed the (a)symmetry of bidirectional forks and found that the two forks proceeding in opposite directions from the same origin mostly travel at similar rates (Figure 7G). This suggests that the replication forks of mES cells do not experience a high level of stalling events due to exogenous or endogenous factors, albeit mES cells are transcriptionally hyperactive (132).

In view of the above mentioned essential parameters for genome duplication once every cell cycle, the karyotype and the resulting genome size of a cell line is important for the determination of replicon characteristics. Most established cell lines used for *in vivo* studies have initially been transformed in order to immortalize them and to revive their replicative potential. Such procedures, however, often introduce undesired genetic aberrations which are known to drastically influence the ploidy of the transformed cell line. Embryonic stem cells, on the other hand, are derived from the inner cell mass (ICM) of the blastocyst and are, by nature, capable of sustaining their proliferative state in culture. Moreover, they have been described to maintain a stable diploid karyotype (133). Nonetheless, we performed karyotype analysis of J1 embryonic stem cells from metaphase chromosome preparations and calculated the approximate genome size taking advantage of published genome data available from the Genome Reference Consortium (92) for haploid mouse genomes. Manual counting of >100 metaphase spreads confirmed a diploid karyotype of the mouse J1 cell line, consisting of 40 acrocentric chromosomes (Figure 7H and Supplementary Table S13). To derive the total genome size, we used the mouse genome assembly GRCm38.p6 mm10 that provides sequencing derived sizes for each chromosome of a haploid mouse genome. Based on this, we calculated a male diploid genome size for mES cells of 5.182 Gb (Figure 7H). Since genome-wide sequencing approaches are affected by unmappable repetitive sequences, this likely is an underestimate of the actual genome size. Indeed, using data from flow cytometry based approaches we obtained diploid mouse genome sizes of 6.03–6.5 Gb (134,135).

### Model for genome replication in embryonic stem versus somatic cells

Using the above mentioned characteristics of mES cell DNA replication and molecular parameters of the associated replicons (i.e. genome size, S-phase duration, number of nanoRFi, IOD and RFS, see Table 1), we analyzed the relationship between replicons and replication foci in mES. The total number of replicons, reflecting the number of origins activated during S-phase, is given by the genome size divided by the average inter origin distance obtained from DNA fiber experiments. This calculation resulted in a total of 57 700–72 300 origins activated during the S-phase of mES cells, depending on how the genome size was estimated. These values are comparable to the number of replication origins obtained by genome-wide origin mapping techniques (see above and (61,62)). Since IOD measurements may be more affected by the sample quality and the length of DNA fibers obtained during the DNA isolation and combing procedure (128), we additionally focused on RFS to determine the number of active replication foci at any given time and to compare it with the actual numbers of foci active in parallel obtained from our 3D-SIM data. The genome size divided by the average speed of a replication fork represents the time required to synthesize the entire genome if only one fork would be active for the entire length of S-phase. Dividing this time by the actual measured S-phase duration, thus effectively represents the number of all active replication forks required at any given time during S-phase. These calculations showed that ∼4750–5950 forks or half as many bidirectional replicons (∼2380–2980) are required to act in parallel in mES cells (Table 1). Comparing the nanoRFi active in parallel counted in 3D-SIM images to the theoretically ‘needed’ RFi active at any given time during S-phase, resulted in a ratio of 0.72–0.9 (2376/3320–2976/3320 RFi), indicating that mES cells activate more replicons than ‘needed’ for genome duplication within the timeframe of S-phase.

**Table 1.** mES cell replicon characteristics

**Experimental data**	**Mean ± SEM**
RFS, 1000 nts per min	1.67 ± 0.02
IOD, kb	90 ± 3.6
Genome size (GS), 1000 Mb	5.19*–6.5**
Active RFi at any given point during S-phase	3 320 ± 20
Total S-phase duration, minutes	654 ± 21
Single forks, %	12.81 ± 8.6
	
**Calculations**	**Mean**
Time to replicate the genome with one fork, (GS/RFS), hours	51 796–64 870
Replication forks active in parallel, (GS/RFS/S-phase duration)	4 752–5 951
Replicons active in parallel, (active forks/2)	2 376–2 976
Replicons per RF, (calculated replicons active in parallel/counted RFi)	0.72–0.9
Calculated % of single forks	10–28

RFS, replication fork speed; nts, nucleotides; IOD, inter-origin distance; GS, genome size (based on published genome data); RF, replication focus; RFi, replication foci; SEM, standard error of the mean; * GS according to mouse reference genome (GRCm38); ** GS according to flow cytometry measurements.

The difference in predicted and measured active replication foci can be explained by a higher frequency of nanoRFi that contain single replication forks and could be resolved by 3D-SIM. The latter would involve the presence of 10–28% of unidirectional forks in mES cells to make up for the difference in calculated versus counted RFi. Indeed, we measured a substantial number of unidirectional replication forks in mES cells using DNA combing amounting to 13% of the total forks detected (Figure 7F). On the other hand, the combination of a less compacted chromatin in mES cells (Supplementary Figure S14) and the resolving power of the 3D-SIM system allows to visualize more individual replication forks in mouse ES cells. Another possibility that could affect the spatial positioning of replication forks or replicons is a different chromatin loop organization in mES cells at sites of ongoing DNA replication, e.g. by subdivision into multiple smaller loops. Hence, we may also detect more individual forks, increasing the number of observed nano replication foci. The differences in heterochromatin compaction in embryonic stem cells (Supplementary Figure S14) are representative of a chromatin organization that is generally more open than that of differentiated cells (78,98). This, in turn, could affect the organization of the DNA fiber, i.e., into chromatin loops. In support of this, it was reported using chromosome conformation capture carbon copy (5C) that the organization of TADs (topologically associated domains) into multiple sub-megabase sized domains (i.e. sub-TAD domains located within TADs) is found in several developmentally regulated genetic loci in mouse ES and neuronal progenitor (NP) cells. Interestingly, in mES cells, a series of on average 100 kb sized loops connects the Sox2 gene with a presumed downstream enhancer element marked by specific histone modifications. Akin to the loss of these epigenetic marks upon differentiation to NP cells, looping interactions were no longer detected in the latter, suggesting a mES cell specific loop organization (136) with loop sizes reflecting replicon sizes that we determined in this study (∼90 kb).

**Figure 8.**
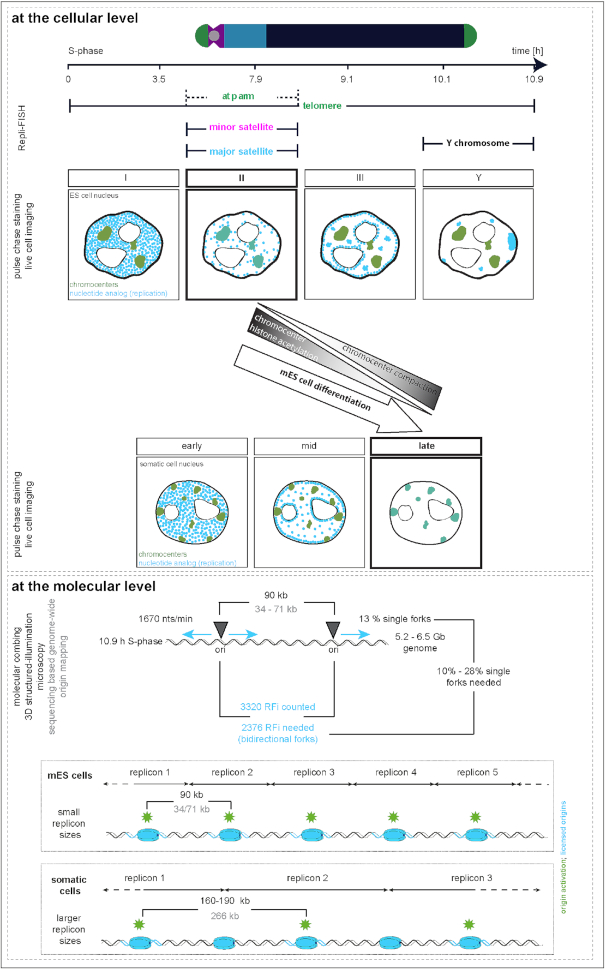
Graphical summary of the cellular and molecular DNA replication characteristics in mouse embryonic stem cells. At the cellular level, DNA replication is visible as distinct replication foci with a dynamic spatio-temporal organization. In mES cells, three different replication patterns are observed during the ∼11 h of S-phase (time progression arrow not scaled). (Sub)chromosomal elements were found to replicate at specific time points during genome duplication. While telomeres located at the long arm of the chromosome replicate throughout S-phase, the ones capping the q arms show significant increase in replication during S-phase stage II. In contrast to somatic mouse cells, (peri)centromeric DNA (marked in green) replicates during mid S-phase (stage II). The Y chromosome is replicated synchronously at the end of S-phase (stage Y) in pluripotent as well as in differentiated cells. Replication timing of pericentromeric DNA switches from early/mid to late S-phase upon mES cell differentiation, which correlates with chromocenter compaction and decreased histone acetylation. At the molecular level, mES cell replicons are characterized by short inter-origin distances of about 90 kb. Replication forks progress at 1.7 nucleotides per minute to replicate the 5.2 Gb mouse genome in about 11 h. Replication is initiated from around 3320 replication foci (RFi), although theoretically ∼2380 bidirectional mES cells are sufficient for genome duplication within the timeframe of S-phase. Hence, from the 3320 sites observed, 28% correspond to single forks and the remaining to bidirectional forks. This is within the range of (13%) single forks observed in DNA fiber analysis. A characteristic arising from the molecular parameters of DNA replication in mouse pluripotent cells are smaller replicon sizes in mES cells relative to somatic cells. Along a given segment of chromosomal DNA carrying multiple licensed origins of replication, mES cells initiate DNA replication from double the number of origins compared to somatic cells. This results in more but smaller replicons and concomitantly smaller inter-origin distances (∼90 kb in mES cell and 160–190 kb in mouse/human somatic cells).

In summary, we analyzed the spatio-temporal dynamics of DNA replication progression in (un)differentiated mouse embryonic stem (mES) cells and compared it with somatic cells. We find a developmental switch in the replication order of main chromosomal domains that is dependent on histone acetylation level (Figure 8). Furthermore, we measured and compared the molecular properties of the mES cell replicon, including the number of replication foci active in parallel and their spatial clustering in mES cells versus somatic cells. We conclude that each replication nanofocus in mES cells corresponds to an individual replicon, with approximately one tenth to one quarter representing unidirectional forks. Furthermore, we find that mES cells activate twice as many origins spaced at half the distance than somatic cells. Altogether, our results highlight fundamental developmental differences on progression of genome replication and origin activation in pluripotent cells.

## DATA AVAILABILITY

All data/code are available at https://doi.org/10.25534/tudatalib-220.

## Supplementary Material

gkaa1124_Supplemental_Files

## Notes

Present address: Corella S. Casas-Delucchi, The Institute of Cancer Research | Chester Beatty Laboratories, London SW3 6JB, UK.

## References

[1] Dimitrova D.S., Gilbert D.M. The spatial position and replication timing of chromosomal domains are both established in early G1 phase. Mol. Cell. 1999; 4:983–993. 10635323 10.1016/s1097-2765(00)80227-0

[2] Hyrien O., Marheineke K., Goldar A. Paradoxes of eukaryotic DNA replication: MCM proteins and the random completion problem. BioEssays. 2003; 25:116–125. 12539237 10.1002/bies.10208

[3] Fragkos M., Ganier O., Coulombe P., Mechali M. DNA replication origin activation in space and time. Nat. Rev. Mol. Cell Biol. 2015; 16:360–374. 25999062 10.1038/nrm4002

[4] Parker M.W., Botchan M.R., Berger J.M. Mechanisms and regulation of DNA replication initiation in eukaryotes. Crit. Rev. Biochem. Mol. Biol. 2017; 52:107–144. 28094588 10.1080/10409238.2016.1274717 PMC5545932

[5] Chagin V.O., Stear J.H., Cardoso M.C. Organization of DNA replication. Cold Spring Harb. Perspect. Biol. 2010; 2:a000737. 20452942 10.1101/cshperspect.a000737 PMC2845211

[6] Jackson D.A., Pombo A. Replicon clusters are stable units of chromosome structure: evidence that nuclear organization contributes to the efficient activation and propagation of S phase in human cells. J. Cell Biol. 1998; 140:1285–1295. 9508763 10.1083/jcb.140.6.1285 PMC2132671

[7] Leonhardt H., Rahn H.P., Weinzierl P., Sporbert A., Cremer T., Zink D., Cardoso M.C. Dynamics of DNA replication factories in living cells. J. Cell Biol. 2000; 149:271–280. 10769021 10.1083/jcb.149.2.271 PMC2175147

[8] Ma H., Samarabandu J., Devdhar R.S., Acharya R., Cheng P.C., Meng C., Berezney R. Spatial and temporal dynamics of DNA replication sites in mammalian cells. J. Cell Biol. 1998; 143:1415–1425. 9852140 10.1083/jcb.143.6.1415 PMC2132991

[9] Manders E.M., Stap J., Brakenhoff G.J., van Driel R., Aten J.A. Dynamics of three-dimensional replication patterns during the S-phase, analysed by double labelling of DNA and confocal microscopy. J. Cell Sci. 1992; 103 (Pt 3):857–862. 1478975 10.1242/jcs.103.3.857

[10] O’Keefe R.T., Henderson S.C., Spector D.L. Dynamic organization of DNA replication in mammalian cell nuclei: spatially and temporally defined replication of chromosome-specific alpha-satellite DNA sequences. J. Cell Biol. 1992; 116:1095–1110. 1740468 10.1083/jcb.116.5.1095 PMC2289349

[11] Marchal C., Sima J., Gilbert D.M. Control of DNA replication timing in the 3D genome. Nat. Rev. Mol. Cell Biol. 2019; 20:721–737. 31477886 10.1038/s41580-019-0162-y PMC11567694

[12] Flockinger R.A., Freedman M.L., Stambrook P.J. Generation times and DNA replication patterns of cells of developing frog embryos. Dev. Biol. 1967; 16:457–473. 6053288 10.1016/0012-1606(67)90059-0

[13] Manders E.M., Stap J., Strackee J., van Driel R., Aten J.A. Dynamic behavior of DNA replication domains. Exp. Cell Res. 1996; 226:328–335. 8806436 10.1006/excr.1996.0233

[14] van Dierendonck J.H., Keyzer R., van de Velde C.J., Cornelisse C.J. Subdivision of S-phase by analysis of nuclear 5-bromodeoxyuridine staining patterns. Cytometry. 1989; 10:143–150. 2469556 10.1002/cyto.990100205

[15] Nakamura H., Morita T., Sato C. Structural organizations of replicon domains during DNA synthetic phase in the mammalian nucleus. Exp. Cell Res. 1986; 165:291–297. 3720850 10.1016/0014-4827(86)90583-5

[16] Nakayasu H., Berezney R. Mapping replicational sites in the eucaryotic cell nucleus. J. Cell Biol. 1989; 108:1–11. 2910875 10.1083/jcb.108.1.1 PMC2115357

[17] Sporbert A., Gahl A., Ankerhold R., Leonhardt H., Cardoso M.C. DNA polymerase clamp shows little turnover at established replication sites but sequential de novo assembly at adjacent origin clusters. Mol. Cell. 2002; 10:1355–1365. 12504011 10.1016/s1097-2765(02)00729-3

[18] Hozak P., Jackson D.A., Cook P.R. Replication factories and nuclear bodies: the ultrastructural characterization of replication sites during the cell cycle. J. Cell Sci. 1994; 107:2191–2202. 7983177 10.1242/jcs.107.8.2191

[19] Friedman K.L., Brewer B.J., Fangman W.L. Replication profile of Saccharomyces cerevisiae chromosome VI. Genes Cells. 1997; 2:667–678. 9491801 10.1046/j.1365-2443.1997.1520350.x

[20] Heinz K.S., Casas-Delucchi C.S., Torok T., Cmarko D., Rapp A., Raska I., Cardoso M.C. Peripheral re-localization of constitutive heterochromatin advances its replication timing and impairs maintenance of silencing marks. Nucleic Acids Res. 2018; 46:6112–6128. 29750270 10.1093/nar/gky368 PMC6158597

[21] Lob D., Lengert N., Chagin V.O., Reinhart M., Casas-Delucchi C.S., Cardoso M.C., Drossel B. 3D replicon distributions arise from stochastic initiation and domino-like DNA replication progression. Nat. Commun. 2016; 7:11207. 27052359 10.1038/ncomms11207 PMC4829661

[22] Yamashita M., Hori Y., Shinomiya T., Obuse C., Tsurimoto T., Yoshikawa H., Shirahige K. The efficiency and timing of initiation of replication of multiple replicons of Saccharomyces cerevisiae chromosome VI. Genes Cells. 1997; 2:655–665. 9491800 10.1046/j.1365-2443.1997.1530351.x

[23] Berezney R., Dubey D.D., Huberman J.A. Heterogeneity of eukaryotic replicons, replicon clusters, and replication foci. Chromosoma. 2000; 108:471–484. 10794569 10.1007/s004120050399

[24] Huberman J.A., Riggs A.D. On the mechanism of DNA replication in mammalian chromosomes. J. Mol. Biol. 1968; 32:327–341. 5689363 10.1016/0022-2836(68)90013-2

[25] Courbet S., Gay S., Arnoult N., Wronka G., Anglana M., Brison O., Debatisse M. Replication fork movement sets chromatin loop size and origin choice in mammalian cells. Nature. 2008; 455:557–560. 18716622 10.1038/nature07233

[26] Guillou E., Ibarra A., Coulon V., Casado-Vela J., Rico D., Casal I., Schwob E., Losada A., Mendez J. Cohesin organizes chromatin loops at DNA replication factories. Genes Dev. 2010; 24:2812–2822. 21159821 10.1101/gad.608210 PMC3003199

[27] Mamberti S., Cardoso M.C. Are the processes of DNA replication and DNA repair reading a common structural chromatin unit. Nucleus. 2020; 11:66–82. 32275847 10.1080/19491034.2020.1744415 PMC7289585

[28] Chagin V.O., Casas-Delucchi C.S., Reinhart M., Schermelleh L., Markaki Y., Maiser A., Bolius J.J., Bensimon A., Fillies M., Domaing P. et al. 4D Visualization of replication foci in mammalian cells corresponding to individual replicons. Nat. Commun. 2016; 7:11231. 27052570 10.1038/ncomms11231 PMC4829660

[29] Reinhart M., Cardoso M.C. A journey through the microscopic ages of DNA replication. Protoplasma. 2017; 254:1151–1162. 27943022 10.1007/s00709-016-1058-8 PMC5376393

[30] Aladjem M.I. Replication in context: dynamic regulation of DNA replication patterns in metazoans. Nat. Rev. Genet. 2007; 8:588–600. 17621316 10.1038/nrg2143

[31] Casas-Delucchi C.S., Brero A., Rahn H.P., Solovei I., Wutz A., Cremer T., Leonhardt H., Cardoso M.C. Histone acetylation controls the inactive X chromosome replication dynamics. Nat. Commun. 2011; 2:222. 21364561 10.1038/ncomms1218 PMC3072080

[32] Casas-Delucchi C.S., van Bemmel J.G., Haase S., Herce H.D., Nowak D., Meilinger D., Stear J.H., Leonhardt H., Cardoso M.C. Histone hypoacetylation is required to maintain late replication timing of constitutive heterochromatin. Nucleic Acids Res. 2012; 40:159–169. 21908399 10.1093/nar/gkr723 PMC3245938

[33] Jorgensen H.F., Azuara V., Amoils S., Spivakov M., Terry A., Nesterova T., Cobb B.S., Ramsahoye B., Merkenschlager M., Fisher A.G. The impact of chromatin modifiers on the timing of locus replication in mouse embryonic stem cells. Genome Biol. 2007; 8:R169. 17705870 10.1186/gb-2007-8-8-r169 PMC2374999

[34] Vogelauer M., Rubbi L., Lucas I., Brewer B.J., Grunstein M. Histone acetylation regulates the time of replication origin firing. Mol. Cell. 2002; 10:1223–1233. 12453428 10.1016/s1097-2765(02)00702-5

[35] Blumenthal A.B., Kriegstein H.J., Hogness D.S. The units of DNA replication in Drosophila melanogaster chromosomes. Cold Spring Harb. Symp. Quant. Biol. 1974; 38:205–223. 4208784 10.1101/sqb.1974.038.01.024

[36] Hyrien O., Maric C., Mechali M. Transition in specification of embryonic metazoan DNA replication origins. Science. 1995; 270:994–997. 7481806 10.1126/science.270.5238.994

[37] Hyrien O., Mechali M. Chromosomal replication initiates and terminates at random sequences but at regular intervals in the ribosomal DNA of Xenopus early embryos. EMBO J. 1993; 12:4511–4520. 8223461 10.1002/j.1460-2075.1993.tb06140.x PMC413880

[38] Walter J., Newport J.W. Regulation of replicon size in Xenopus egg extracts. Science. 1997; 275:993–995. 9020085 10.1126/science.275.5302.993

[39] Hamatani T., Carter M.G., Sharov A.A., Ko M.S. Dynamics of global gene expression changes during mouse preimplantation development. Dev. Cell. 2004; 6:117–131. 14723852 10.1016/s1534-5807(03)00373-3

[40] Sansam C.G., Goins D., Siefert J.C., Clowdus E.A., Sansam C.L. Cyclin-dependent kinase regulates the length of S phase through TICRR/TRESLIN phosphorylation. Genes Dev. 2015; 29:555–566. 25737283 10.1101/gad.246827.114 PMC4358407

[41] Torres-Padilla M.E., Zernicka-Goetz M. Role of TIF1alpha as a modulator of embryonic transcription in the mouse zygote. J. Cell Biol. 2006; 174:329–338. 16880268 10.1083/jcb.200603146 PMC2064229

[42] Ferreira J., Carmo-Fonseca M. Genome replication in early mouse embryos follows a defined temporal and spatial order. J. Cell Sci. 1997; 110:889–897. 9133676 10.1242/jcs.110.7.889

[43] Hiratani I., Ryba T., Itoh M., Yokochi T., Schwaiger M., Chang C.W., Lyou Y., Townes T.M., Schubeler D., Gilbert D.M. Global reorganization of replication domains during embryonic stem cell differentiation. PLoS Biol. 2008; 6:e245. 18842067 10.1371/journal.pbio.0060245 PMC2561079

[44] Rivera-Mulia J.C., Buckley Q., Sasaki T., Zimmerman J., Didier R.A., Nazor K., Loring J.F., Lian Z., Weissman S., Robins A.J. et al. Dynamic changes in replication timing and gene expression during lineage specification of human pluripotent stem cells. Genome Res. 2015; 25:1091–1103. 26055160 10.1101/gr.187989.114 PMC4509994

[45] Li E., Bestor T.H., Jaenisch R. Targeted mutation of the DNA methyltransferase gene results in embryonic lethality. Cell. 1992; 69:915–926. 1606615 10.1016/0092-8674(92)90611-f

[46] Doetschman T., Gregg R.G., Maeda N., Hooper M.L., Melton D.W., Thompson S., Smithies O. Targetted correction of a mutant HPRT gene in mouse embryonic stem cells. Nature. 1987; 330:576–578. 3683574 10.1038/330576a0

[47] Peters A.H., O’Carroll D., Scherthan H., Mechtler K., Sauer S., Schofer C., Weipoltshammer K., Pagani M., Lachner M., Kohlmaier A. et al. Loss of the Suv39h histone methyltransferases impairs mammalian heterochromatin and genome stability. Cell. 2001; 107:323–337. 11701123 10.1016/s0092-8674(01)00542-6

[48] Yaffe D., Saxel O. Serial passaging and differentiation of myogenic cells isolated from dystrophic mouse muscle. Nature. 1977; 270:725–727. 563524 10.1038/270725a0

[49] Bertulat B., De Bonis M.L., Della Ragione F., Lehmkuhl A., Milden M., Storm C., Jost K.L., Scala S., Hendrich B., D’Esposito M. et al. MeCP2 dependent heterochromatin reorganization during neural differentiation of a novel Mecp2-deficient embryonic stem cell reporter line. PLoS One. 2012; 7:e47848. 23112857 10.1371/journal.pone.0047848 PMC3480415

[50] Sporbert A., Domaing P., Leonhardt H., Cardoso M.C. PCNA acts as a stationary loading platform for transiently interacting Okazaki fragment maturation proteins. Nucleic Acids Res. 2005; 33:3521–3528. 15972794 10.1093/nar/gki665 PMC1156965

[51] Lindhout B.I., Fransz P., Tessadori F., Meckel T., Hooykaas P.J., van der Zaal B.J. Live cell imaging of repetitive DNA sequences via GFP-tagged polydactyl zinc finger proteins. Nucleic Acids Res. 2007; 35:e107. 17704126 10.1093/nar/gkm618 PMC2018617

[52] Casas-Delucchi C.S., Becker A., Bolius J.J., Cardoso M.C. Targeted manipulation of heterochromatin rescues MeCP2 Rett mutants and re-establishes higher order chromatin organization. Nucleic Acids Res. 2012; 40:e176. 22923521 10.1093/nar/gks784 PMC3526307

[53] Chagin V.O., Reinhart M., Cardoso M.C. High-resolution analysis of mammalian DNA replication units. Methods Mol. Biol. 2015; 1300:43–65. 25916704 10.1007/978-1-4939-2596-4_3

[54] Bialic M., Coulon V., Drac M., Gostan T., Schwob E. Analyzing the dynamics of DNA replication in Mammalian cells using DNA combing. Methods Mol. Biol. 2015; 1300:67–78. 25916705 10.1007/978-1-4939-2596-4_4

[55] Weber P., Rausch C., Scholl A., Cardoso M.C. Repli-FISH (fluorescence in situ hybridization): application of 3D-(immuno)-FISH for the study of DNA replication timing of genetic repeat elements. OBM Genet. 2019; 3:e1325.

[56] Gustafsson M.G., Shao L., Carlton P.M., Wang C.J., Golubovskaya I.N., Cande W.Z., Agard D.A., Sedat J.W. Three-dimensional resolution doubling in wide-field fluorescence microscopy by structured illumination. Biophys. J. 2008; 94:4957–4970. 18326650 10.1529/biophysj.107.120345 PMC2397368

[57] Berg S., Kutra D., Kroeger T., Straehle C.N., Kausler B.X., Haubold C., Schiegg M., Ales J., Beier T., Rudy M. et al. ilastik: interactive machine learning for (bio)image analysis. Nat. Methods. 2019; 16:1226–1232. 31570887 10.1038/s41592-019-0582-9

[58] van der Walt S., Schönberger J.L., Nunez-Iglesias J., Boulogne F., Warner J.D., Yager N., Gouillart E., Yu T. contributors, t.s.-i. scikit-image: image processing in Python. PeerJ. 2014; 2:e453. 25024921 10.7717/peerj.453 PMC4081273

[59] Schindelin J., Arganda-Carreras I., Frise E., Kaynig V., Longair M., Pietzsch T., Preibisch S., Rueden C., Saalfeld S., Schmid B. et al. Fiji: an open-source platform for biological-image analysis. Nat. Methods. 2012; 9:676–682. 22743772 10.1038/nmeth.2019 PMC3855844

[60] Natale F., Rapp A., Yu W., Maiser A., Harz H., Scholl A., Grulich S., Anton T., Horl D., Chen W. et al. Identification of the elementary structural units of the DNA damage response. Nat. Commun. 2017; 8:15760. 28604675 10.1038/ncomms15760 PMC5472794

[61] Prorok P., Artufel M., Aze A., Coulombe P., Peiffer I., Lacroix L., Guedin A., Mergny J.L., Damaschke J., Schepers A. et al. Involvement of G-quadruplex regions in mammalian replication origin activity. Nat. Commun. 2019; 10:3274. 31332171 10.1038/s41467-019-11104-0 PMC6646384

[62] Almeida R., Fernandez-Justel J.M., Santa-Maria C., Cadoret J.C., Cano-Aroca L., Lombrana R., Herranz G., Agresti A., Gomez M. Chromatin conformation regulates the coordination between DNA replication and transcription. Nat. Commun. 2018; 9:1590. 29686321 10.1038/s41467-018-03539-8 PMC5913246

[63] Quinlan A.R., Hall I.M. BEDTools: a flexible suite of utilities for comparing genomic features. Bioinformatics. 2010; 26:841–842. 20110278 10.1093/bioinformatics/btq033 PMC2832824

[64] Tubbs A., Sridharan S., van Wietmarschen N., Maman Y., Callen E., Stanlie A., Wu W., Wu X., Day A., Wong N. et al. Dual roles of Poly(dA:dT) tracts in replication initiation and fork collapse. Cell. 2018; 174:1127–1142. 30078706 10.1016/j.cell.2018.07.011 PMC6591735

[65] Hansen R.S., Thomas S., Sandstrom R., Canfield T.K., Thurman R.E., Weaver M., Dorschner M.O., Gartler S.M., Stamatoyannopoulos J.A. Sequencing newly replicated DNA reveals widespread plasticity in human replication timing. PNAS. 2010; 107:139–144. 19966280 10.1073/pnas.0912402107 PMC2806781

[66] Pope B.D., Hiratani I., Gilbert D.M. Domain-wide regulation of DNA replication timing during mammalian development. Chromosome Res. 2010; 18:127–136. 20013151 10.1007/s10577-009-9100-8 PMC2827620

[67] Woodfine K., Beare D.M., Ichimura K., Debernardi S., Mungall A.J., Fiegler H., Collins V.P., Carter N.P., Dunham I. Replication timing of human chromosome 6. Cell Cycle. 2005; 4:172–176. 15611667 10.4161/cc.4.1.1350

[68] Woodfine K., Fiegler H., Beare D.M., Collins J.E., McCann O.T., Young B.D., Debernardi S., Mott R., Dunham I., Carter N.P. Replication timing of the human genome. Hum. Mol. Genet. 2004; 13:191–202. 14645202 10.1093/hmg/ddh016

[69] Natale F., Scholl A., Rapp A., Yu W., Rausch C., Cardoso M.C. DNA replication and repair kinetics of Alu, LINE-1 and satellite III genomic repetitive elements. Epigenet. Chromatin. 2018; 11:61. 10.1186/s13072-018-0226-9 PMC6198450 30352618

[70] Shermoen A.W., McCleland M.L., O’Farrell P.H. Developmental control of late replication and S phase length. Curr. Biol. 2010; 20:2067–2077. 21074439 10.1016/j.cub.2010.10.021 PMC3108027

[71] Jeppesen P., Mitchell A., Turner B., Perry P. Antibodies to defined histone epitopes reveal variations in chromatin conformation and underacetylation of centric heterochromatin in human metaphase chromosomes. Chromosoma. 1992; 101:322–332. 1374304 10.1007/BF00346011

[72] Guenatri M., Bailly D., Maison C., Almouzni G. Mouse centric and pericentric satellite repeats form distinct functional heterochromatin. J. Cell Biol. 2004; 166:493–505. 15302854 10.1083/jcb.200403109 PMC2172221

[73] Brero A., Easwaran H.P., Nowak D., Grunewald I., Cremer T., Leonhardt H., Cardoso M.C. Methyl CpG-binding proteins induce large-scale chromatin reorganization during terminal differentiation. J. Cell Biol. 2005; 169:733–743. 15939760 10.1083/jcb.200502062 PMC2171616

[74] Mayer R., Brero A., von Hase J., Schroeder T., Cremer T., Dietzel S. Common themes and cell type specific variations of higher order chromatin arrangements in the mouse. BMC Cell Biol. 2005; 6:44. 16336643 10.1186/1471-2121-6-44 PMC1325247

[75] Solovei I., Joffe B. Inverted nuclear architecture and its development during differentiation of mouse rod photoreceptor cells: a new model to study nuclear architecture. Genetika. 2010; 46:1159–1163. 21058510

[76] Solovei I., Kreysing M., Lanctot C., Kosem S., Peichl L., Cremer T., Guck J., Joffe B. Nuclear architecture of rod photoreceptor cells adapts to vision in mammalian evolution. Cell. 2009; 137:356–368. 19379699 10.1016/j.cell.2009.01.052

[77] Nozaki T., Imai R., Tanbo M., Nagashima R., Tamura S., Tani T., Joti Y., Tomita M., Hibino K., Kanemaki M.T. et al. Dynamic organization of chromatin domains revealed by super-resolution live-cell imaging. Mol. Cell. 2017; 67:282–293. 28712725 10.1016/j.molcel.2017.06.018

[78] Lopes Novo C., Rugg-Gunn P.J. Chromatin organization in pluripotent cells: emerging approaches to study and disrupt function. Brief. Funct. Genomics. 2016; 15:305–314. 26206085 10.1093/bfgp/elv029 PMC4958138

[79] Meshorer E., Misteli T. Chromatin in pluripotent embryonic stem cells and differentiation. Nat. Rev. Mol. Cell Biol. 2006; 7:540–546. 16723974 10.1038/nrm1938

[80] Gaspar-Maia A., Alajem A., Polesso F., Sridharan R., Mason M.J., Heidersbach A., Ramalho-Santos J., McManus M.T., Plath K., Meshorer E. et al. Chd1 regulates open chromatin and pluripotency of embryonic stem cells. Nature. 2009; 460:863–868. 19587682 10.1038/nature08212 PMC3891576

[81] Kobayakawa S., Miike K., Nakao M., Abe K. Dynamic changes in the epigenomic state and nuclear organization of differentiating mouse embryonic stem cells. Genes Cells. 2007; 12:447–460. 17397393 10.1111/j.1365-2443.2007.01063.x

[82] Aparicio J.G., Viggiani C.J., Gibson D.G., Aparicio O.M. The Rpd3-Sin3 histone deacetylase regulates replication timing and enables intra-S origin control in Saccharomyces cerevisiae. Mol. Cell. Biol. 2004; 24:4769–4780. 15143171 10.1128/MCB.24.11.4769-4780.2004 PMC416400

[83] Bickmore W.A., Carothers A.D. Factors affecting the timing and imprinting of replication on a mammalian chromosome. J. Cell Sci. 1995; 108 (Pt 8):2801–2809. 7593321 10.1242/jcs.108.8.2801

[84] Goren A., Tabib A., Hecht M., Cedar H. DNA replication timing of the human beta-globin domain is controlled by histone modification at the origin. Genes Dev. 2008; 22:1319–1324. 18443145 10.1101/gad.468308 PMC2377185

[85] Kemp M.G., Ghosh M., Liu G., Leffak M. The histone deacetylase inhibitor trichostatin A alters the pattern of DNA replication origin activity in human cells. Nucleic Acids Res. 2005; 33:325–336. 15653633 10.1093/nar/gki177 PMC546162

[86] Keohane A.M., O’Neill L.P., Belyaev N.D., Lavender J.S., Turner B.M. X-Inactivation and histone H4 acetylation in embryonic stem cells. Dev. Biol. 1996; 180:618–630. 8954732 10.1006/dbio.1996.0333

[87] Lin C.M., Fu H., Martinovsky M., Bouhassira E., Aladjem M.I. Dynamic alterations of replication timing in mammalian cells. Curr. Biol. 2003; 13:1019–1028. 12814547 10.1016/s0960-9822(03)00382-8

[88] Perry P., Sauer S., Billon N., Richardson W.D., Spivakov M., Warnes G., Livesey F.J., Merkenschlager M., Fisher A.G., Azuara V. A dynamic switch in the replication timing of key regulator genes in embryonic stem cells upon neural induction. Cell Cycle. 2004; 3:1645–1650. 15611653

[89] Schubeler D., Francastel C., Cimbora D.M., Reik A., Martin D.I., Groudine M. Nuclear localization and histone acetylation: a pathway for chromatin opening and transcriptional activation of the human beta-globin locus. Genes Dev. 2000; 14:940–950. 10783166 PMC316536

[90] Heinz K.S., Cardoso M.C. Targeted manipulation/repositioning of subcellular structures and molecules. Methods Mol. Biol. 2019; 2038:199–208. 31407286 10.1007/978-1-4939-9674-2_13

[91] Lander E.S., Linton L.M., Birren B., Nusbaum C., Zody M.C., Baldwin J., Devon K., Dewar K., Doyle M., FitzHugh W. et al. Initial sequencing and analysis of the human genome. Nature. 2001; 409:860–921. 11237011 10.1038/35057062

[92] Mouse Genome Sequencing, C. Waterston R.H., Lindblad-Toh K., Birney E., Rogers J., Abril J.F., Agarwal P., Agarwala R., Ainscough R., Alexandersson M. et al. Initial sequencing and comparative analysis of the mouse genome. Nature. 2002; 420:520–562. 12466850 10.1038/nature01262

[93] Bouwman B.A., de Laat W. Getting the genome in shape: the formation of loops, domains and compartments. Genome Biol. 2015; 16:154. 26257189 10.1186/s13059-015-0730-1 PMC4536798

[94] Krijger P.H., de Laat W. Identical cells with different 3D genomes; cause and consequences. Curr. Opin. Genet. Dev. 2013; 23:191–196. 23415810 10.1016/j.gde.2012.12.010

[95] Politz J.C., Scalzo D., Groudine M. Something silent this way forms: the functional organization of the repressive nuclear compartment. Annu. Rev. Cell Dev. Biol. 2013; 29:241–270. 23834025 10.1146/annurev-cellbio-101512-122317 PMC3999972

[96] Solovei I., Thanisch K., Feodorova Y. How to rule the nucleus: divide et impera. Curr. Opin. Cell Biol. 2016; 40:47–59. 26938331 10.1016/j.ceb.2016.02.014

[97] Ahmad K., Henikoff S. Centromeres are specialized replication domains in heterochromatin. J. Cell Biol. 2001; 153:101–110. 11285277 10.1083/jcb.153.1.101 PMC2185517

[98] McCarroll R.M., Fangman W.L. Time of replication of yeast centromeres and telomeres. Cell. 1988; 54:505–513. 3042152 10.1016/0092-8674(88)90072-4

[99] Chagin V.O., Reinhart B., Becker A., Mortusewicz O., Jost K.L., Rapp A., Leonhardt H., Cardoso M.C. Processive DNA synthesis is associated with localized decompaction of constitutive heterochromatin at the sites of DNA replication and repair. Nucleus. 2019; 10:231–253. 31744372 10.1080/19491034.2019.1688932 PMC6949026

[100] Weidtkamp-Peters S., Rahn H.P., Cardoso M.C., Hemmerich P. Replication of centromeric heterochromatin in mouse fibroblasts takes place in early, middle, and late S phase. Histochem. Cell Biol. 2006; 125:91–102. 16231189 10.1007/s00418-005-0063-3

[101] Arnoult N., Schluth-Bolard C., Letessier A., Drascovic I., Bouarich-Bourimi R., Campisi J., Kim S.H., Boussouar A., Ottaviani A., Magdinier F. et al. Replication timing of human telomeres is chromosome arm-specific, influenced by subtelomeric structures and connected to nuclear localization. PLoS Genet. 2010; 6:e1000920. 20421929 10.1371/journal.pgen.1000920 PMC2858680

[102] Jobling M.A., Tyler-Smith C. The human Y chromosome: an evolutionary marker comes of age. Nat. Rev. Genet. 2003; 4:598–612. 12897772 10.1038/nrg1124

[103] Singh N.P., Madabhushi S.R., Srivastava S., Senthilkumar R., Neeraja C., Khosla S., Mishra R.K. Epigenetic profile of the euchromatic region of human Y chromosome. Nucleic Acids Res. 2011; 39:3594–3606. 21252296 10.1093/nar/gkq1342 PMC3089472

[104] Aken B.L., Ayling S., Barrell D., Clarke L., Curwen V., Fairley S., Fernandez Banet J., Billis K., Garcia Giron C., Hourlier T. et al. The Ensembl gene annotation system. Database. 2016; 2016:baw093. 27337980 10.1093/database/baw093 PMC4919035

[105] Skaletsky H., Kuroda-Kawaguchi T., Minx P.J., Cordum H.S., Hillier L., Brown L.G., Repping S., Pyntikova T., Ali J., Bieri T. et al. The male-specific region of the human Y chromosome is a mosaic of discrete sequence classes. Nature. 2003; 423:825–837. 12815422 10.1038/nature01722

[106] Maan A.A., Eales J., Akbarov A., Rowland J., Xu X., Jobling M.A., Charchar F.J., Tomaszewski M. The Y chromosome: a blueprint for men's health. Eur. J. Hum. Genet. 2017; 25:1181–1188. 28853720 10.1038/ejhg.2017.128 PMC5643963

[107] Charlesworth B. The organization and evolution of the human Y chromosome. Genome Biol. 2003; 4:226. 12952526 10.1186/gb-2003-4-9-226 PMC193647

[108] Quintana-Murci L., Fellous M. The human Y chromosome: the biological role of a “functional wasteland”. J. Biomed. Biotechnol. 2001; 1:18–24. 12488622 10.1155/S1110724301000080 PMC79676

[109] Schubeler D., Scalzo D., Kooperberg C., van Steensel B., Delrow J., Groudine M. Genome-wide DNA replication profile for Drosophila melanogaster: a link between transcription and replication timing. Nat. Genet. 2002; 32:438–442. 12355067 10.1038/ng1005

[110] Cadoret J.C., Meisch F., Hassan-Zadeh V., Luyten I., Guillet C., Duret L., Quesneville H., Prioleau M.N. Genome-wide studies highlight indirect links between human replication origins and gene regulation. PNAS. 2008; 105:15837–15842. 18838675 10.1073/pnas.0805208105 PMC2572913

[111] Costantini M., Bernardi G. Replication timing, chromosomal bands, and isochores. PNAS. 2008; 105:3433–3437. 18305168 10.1073/pnas.0710587105 PMC2265141

[112] Picard F., Cadoret J.C., Audit B., Arneodo A., Alberti A., Battail C., Duret L., Prioleau M.N. The spatiotemporal program of DNA replication is associated with specific combinations of chromatin marks in human cells. PLoS Genet. 2014; 10:e1004282. 24785686 10.1371/journal.pgen.1004282 PMC4006723

[113] Blow J.J., Gillespie P.J., Francis D., Jackson D.A. Replication origins in Xenopus egg extract Are 5–15 kilobases apart and are activated in clusters that fire at different times. J. Cell Biol. 2001; 152:15–25. 11149917 10.1083/jcb.152.1.15 PMC2193667

[114] Mills A.D., Blow J.J., White J.G., Amos W.B., Wilcock D., Laskey R.A. Replication occurs at discrete foci spaced throughout nuclei replicating in vitro. J. Cell Sci. 1989; 94:471–477. 2632579 10.1242/jcs.94.3.471

[115] Newport J., Kirschner M. A major developmental transition in early Xenopus embryos: II. Control of the onset of transcription. Cell. 1982; 30:687–696. 7139712 10.1016/0092-8674(82)90273-2

[116] Newport J., Kirschner M. A major developmental transition in early Xenopus embryos: I. Characterization and timing of cellular changes at the midblastula stage. Cell. 1982; 30:675–686. 6183003 10.1016/0092-8674(82)90272-0

[117] Li V.C., Ballabeni A., Kirschner M.W. Gap 1 phase length and mouse embryonic stem cell self-renewal. PNAS. 2012; 109:12550–12555. 22802651 10.1073/pnas.1206740109 PMC3412034

[118] Burdon T., Smith A., Savatier P. Signalling, cell cycle and pluripotency in embryonic stem cells. Trends Cell Biol. 2002; 12:432–438. 12220864 10.1016/s0962-8924(02)02352-8

[119] Soufi A., Dalton S. Cycling through developmental decisions: how cell cycle dynamics control pluripotency, differentiation and reprogramming. Development. 2016; 143:4301–4311. 27899507 10.1242/dev.142075 PMC5201050

[120] White J., Dalton S. Cell cycle control of embryonic stem cells. Stem Cell Rev. 2005; 1:131–138. 17142847 10.1385/SCR:1:2:131

[121] Tamm C., Pijuan Galito S., Anneren C. A comparative study of protocols for mouse embryonic stem cell culturing. PLoS One. 2013; 8:e81156. 24339907 10.1371/journal.pone.0081156 PMC3858223

[122] Dixon J.R., Jung I., Selvaraj S., Shen Y., Antosiewicz-Bourget J.E., Lee A.Y., Ye Z., Kim A., Rajagopal N., Xie W. et al. Chromatin architecture reorganization during stem cell differentiation. Nature. 2015; 518:331–336. 25693564 10.1038/nature14222 PMC4515363

[123] Meshorer E., Yellajoshula D., George E., Scambler P.J., Brown D.T., Misteli T. Hyperdynamic plasticity of chromatin proteins in pluripotent embryonic stem cells. Dev. Cell. 2006; 10:105–116. 16399082 10.1016/j.devcel.2005.10.017 PMC1868458

[124] Pekowska A., Klaus B., Xiang W., Severino J., Daigle N., Klein F.A., Oles M., Casellas R., Ellenberg J., Steinmetz L.M. et al. Gain of CTCF-Anchored chromatin loops marks the exit from naive pluripotency. Cell Syst. 2018; 7:482–495. 30414923 10.1016/j.cels.2018.09.003 PMC6327227

[125] Fraser P. Transcriptional control thrown for a loop. Curr. Opin. Genet. Dev. 2006; 16:490–495. 16904310 10.1016/j.gde.2006.08.002

[126] Tolhuis B., Palstra R.J., Splinter E., Grosveld F., de Laat W. Looping and interaction between hypersensitive sites in the active beta-globin locus. Mol. Cell. 2002; 10:1453–1465. 12504019 10.1016/s1097-2765(02)00781-5

[127] Buongiorno-Nardelli M., Micheli G., Carri M.T., Marilley M. A relationship between replicon size and supercoiled loop domains in the eukaryotic genome. Nature. 1982; 298:100–102. 7088157 10.1038/298100a0

[128] Techer H., Koundrioukoff S., Azar D., Wilhelm T., Carignon S., Brison O., Debatisse M., Le Tallec B. Replication dynamics: biases and robustness of DNA fiber analysis. J. Mol. Biol. 2013; 425:4845–4855. 23557832 10.1016/j.jmb.2013.03.040

[129] Cardoso M.C., Leonhardt H. DNA methyltransferase is actively retained in the cytoplasm during early development. J. Cell Biol. 1999; 147:25–32. 10508852 10.1083/jcb.147.1.25 PMC2164986

[130] Cayrou C., Gregoire D., Coulombe P., Danis E., Mechali M. Genome-scale identification of active DNA replication origins. Methods. 2012; 57:158–164. 22796403 10.1016/j.ymeth.2012.06.015

[131] Petryk N., Kahli M., d’Aubenton-Carafa Y., Jaszczyszyn Y., Shen Y., Silvain M., Thermes C., Chen C.L., Hyrien O. Replication landscape of the human genome. Nat. Commun. 2016; 7:10208. 26751768 10.1038/ncomms10208 PMC4729899

[132] Efroni S., Duttagupta R., Cheng J., Dehghani H., Hoeppner D.J., Dash C., Bazett-Jones D.P., Le Grice S., McKay R.D., Buetow K.H. et al. Global transcription in pluripotent embryonic stem cells. Cell Stem Cell. 2008; 2:437–447. 18462694 10.1016/j.stem.2008.03.021 PMC2435228

[133] Martin G.R. Isolation of a pluripotent cell line from early mouse embryos cultured in medium conditioned by teratocarcinoma stem cells. PNAS. 1981; 78:7634–7638. 6950406 10.1073/pnas.78.12.7634 PMC349323

[134] Capparelli R., Cottone C., D’Apice L., Viscardi M., Colantonio L., Lucretti S., Iannelli D. DNA content differences in laboratory mouse strains determined by flow cytometry. Cytometry. 1997; 29:261–266. 9389443 10.1002/(sici)1097-0320(19971101)29:3<261::aid-cyto9>3.0.co;2-e

[135] Vinogradov A.E. Genome size and GC-percent in vertebrates as determined by flow cytometry: the triangular relationship. Cytometry. 1998; 31:100–109. 9482279 10.1002/(sici)1097-0320(19980201)31:2<100::aid-cyto5>3.0.co;2-q

[136] Phillips-Cremins J.E., Sauria M.E., Sanyal A., Gerasimova T.I., Lajoie B.R., Bell J.S., Ong C.T., Hookway T.A., Guo C., Sun Y. et al. Architectural protein subclasses shape 3D organization of genomes during lineage commitment. Cell. 2013; 153:1281–1295. 23706625 10.1016/j.cell.2013.04.053 PMC3712340

