# Supplementary material for: Developmental differences in genome replication program and origin activation
Source: Nucleic Acids Res. 2020 Dec 2;48(22):12751–77. doi: 10.1093/nar/gkaa1124 (PMC7736824; doi:10.1093/nar/gkaa1124)
Supplement: gkaa1124_Supplemental_Files [file gkaa1124_supplemental_files.zip › NAR-02027-V-2020_Supplementary.pdf]

SUPPLEMENTARY TABLES, FIGURES AND MOVIES

Developmental differences in genome replication program and origin activation

Cathia Rausch[1,†], Patrick Weber[1,†], Paulina Prorok[1], David Hörl[2], Andreas Maiser[2], Anne Lehmkuhl[1], Vadim O. Chagin[1,3], Corella S. Casas-Delucchi[1], Heinrich Leonhardt[2] and M. Cristina Cardoso[1,*]

1 Department of Biology, Technical University of Darmstadt, 64287 Darmstadt, Germany
2 Department of Biology II, LMU Munich, 81377 Munich, Germany
3 Institute of Cytology, Russian Academy of Sciences, St. Petersburg, Russia
† These authors contributed equally to this work.
* To whom correspondence should be addressed. Tel: +49 6151 16 21882; Fax: +49 6151 16 21880; Email: cardoso@bio.tu-darmstadt.de.
Present address: Corella S. Casas-Delucchi, The Institute of Cancer Research | Chester Beatty Laboratories, London, SW3 6JB, United Kingdom.

SUPPLEMENTARY TABLES

Supplementary Table 1: Cell line characteristics.

Name	Species	Type	Genotype	Reference
J1 wt	Mus musculus	embryonic stem cell	wildtype	(1)
E14 wt	Mus musculus	embryonic stem cell	wildtype	(2)
C2C12	Mus musculus	myoblast	wildtype	(3)
MEF W8	Mus musculus	embryonic fibroblast	wildtype	(4)
Primary fibroblast	Mus musculus	primary ear fibroblast*	wildtype	-

* from adult C57BL/6 mice.

Supplementary Table 2: Plasmid characteristics.

Name	pc number*	Fluorophore	Gene species	Promoter	Reference
pmRFP-PCNA	1054	mRFP	Homo sapiens	CMV	(5)
pMaSat-GFP	1803	GFP	-	CMV	(6)
pGBP-MaSat	2469	-	-	CMV	(7)
peGFP-HDAC1	2447	eGFP	Homo sapiens	CMV	This study
peGFP-C1	592	eGFP	Aequorea victoria	CMV	Clontech Laboratories, Mountain View, CA, USA

* pc: plasmid collection.

Supplementary Table 3: Nucleotide characteristics.

Name	Application	Detection	Cat #	Company
Biotin-16-dUTP	Labeling of FISH* probes	Streptavidin	-	Self made (8)
Dig-11-dUTP	Labeling of FISH probes	Antibody detection	11573152910	Sigma-Aldrich, St Louis, MO, USA
Cy3-dUTP	Labeling of FISH probes	-	PA53022	GE Healthcare, Chicago, IL, USA
Cy5-dUTP	Labeling of FISH probes	-	PA55022	GE Healthcare, Chicago, IL, USA
dATP, dTTP, dCTP & dGTP	Generation of FISH probes	-	10297018	Thermo Fisher Scientific, Waltham, MA, USA
5-ethynyl-2'-deoxyuridine (EdU)	Labeling of nascent DNA in pulse (chase) experiments	ClickIT chemistry	E10415	Thermo Fisher Scientific, Waltham, MA, USA
5-bromo-2'-deoxyuridine (BrdU)	Labeling of nascent DNA in pulse (chase) experiments	Antibody detection	B5002	Sigma-Aldrich, St Louis, MO, USA
Thymidine	Labeling of nascent DNA in pulse (chase) experiments**	-	T9250	Sigma-Aldrich, St Louis, MO, USA
5-iodo-2'-deoxyuridine (IdU)	Labeling of nascent DNA in molecular DNA combing experiments	Antibody detection	I7125	Sigma-Aldrich, St Louis, MO, USA
5-chloro-2'-deoxyuridine (CldU)	Labeling of nascent DNA in molecular DNA combing experiments	Antibody detection	C6891	Sigma-Aldrich, St Louis, MO, USA

* fluorescence *in situ* hybridization, ** added only during chase period in pulse chase experiments.

Supplementary Table 4: Primary and secondary antibody characteristics.

Reactivity	Host	Dilution	Application	Cat #	Company
anti-PCNA (PC-10)	Mouse	1:100	IF*	M0879	Dako, Hamburg, Germany
anti-Oct3/4 (40/Oct-3)	Mouse	1:1000	IF	611203	BD Bioscience, San Jose, CA, USA
anti-H3K9ac (1B10)	Mouse	1:500	IF	61251	Active Motif, La Hulpe, Belgium
anti-BrdU/IdU (B44)	Mouse	1:200	Molecular combing IF	347580	Becton Dickinson, Franklin Lakes, NJ, USA
anti-ssDNA (16-19, IgG2a)	Mouse	1:200	Molecular combing IF	MAB3034	Millipore, Burlington, MA, USA
anti-Sox2 (EPR3131)	Rabbit	1:200	IF	ab92494	Abcam, Cambridge, UK
anti-H4K8ac	Rabbit	1:100	IF	06-760	Upstate, Lake Placid, NY, USA
anti-H3K9m3	Rabbit	1:200	IF	39161	Active Motif, La Hulpe, Belgium
anti-H4K5ac (EP1000Y)	Rabbit	1:500	IF	1808	Epitomics, Burlingame, CA, USA
anti-digoxigenin	Rabbit	1:500	FISH** probe detection	700772	Thermo Fisher Scientific, Waltham, MA, USA
anti-BrdU/CldU (BU1/75 (ICR1))	Rat	1:100/1:200	IF/Molecular combing IF	OBT0030CX	BioRad, Puchheim, Germany
anti-GFP (7.1 & 13.1)	Mouse	1:500	FISH IF	11814460001	Roche, Penzberg, Germany
anti-mouse Alexa488	Donkey	1:300	IF (fluorescent secondary)	715-545-150	The Jackson Laboratory, Bar Harbor, ME, USA
anti-mouse IgG Cy3	Donkey	1:300	IF (fluorescent secondary)	715-165-151	The Jackson Laboratory, Bar Harbor, ME, USA
anti-mouse IgG Chromeo 546	Goat	1:200	Molecular combing IF (fluorescent secondary)	15033	Active Motif, La Hulpe, Belgium
anti-mouse IgG AlexaFluor 594	Donkey	1:500	IF (fluorescent secondary)	715-585-151	The Jackson Laboratory, Bar Harbor, ME, USA

Reactivity	Host	Dilution	Application	Cat #	Company
anti-mouse IgG Alexa647	Goat	1:300	IF (fluorescent secondary)	A-32728	Molecular Probes Inc., Eugene, OR, USA
anti-mouse IgG2a AlexaFluor 647	Goat	1:200	Molecular combing IF (fluorescent secondary)	A-21241	Fisher Scientific GmbH, Hampton, NH, USA
anti-rabbit IgG Cy3	Donkey	1:300	IF (fluorescent secondary)/FISH probe detection	711-165-152	The Jackson Laboratory, Bar Harbor, ME, USA
anti-rabbit IgG Alexa647	Goat	1:300	IF (fluorescent secondary)	A-21245	Molecular Probes Inc., Eugene, OR, USA
anti-rat IgG AlexaFluor 488	Donkey	1:500/1:200	IF/Molecular combing IF (fluorescent secondary)	712-545-153	The Jackson Laboratory, Bar Harbor, ME, USA

* IF: immunofluorescence, ** fluorescence *in situ* hybridization.

Supplementary Table 5: Imaging systems characteristics.

Microscope/ Company	Lasers/lamps	Filters (ex. & em. [nm])*	Objectives/ lenses	Detection system	Incubation system	Application
Ultra-View VoX spinning disk on an inverted Nikon Ti-E microscope/ PerkinElmer Life Sciences, UK	solid state diode lasers (405 nm, 488 nm, 561 nm, 640 nm)	405/488/568/640** 405: 415–475 488: 505–549 561: 580–650 640: 664–754	oil immersion 60x Plan-Apochromat (NA 1.45)	cooled 14-bit Hamamatsu® C9100-50 EMCCD	closed live-cell microscopy chamber (ACU control, Olympus) for time-lapse microscopy	time-lapse microscopy & confocal z-stack imaging
Widefield Axiovert 200 /Zeiss, Germany	HBO100 mercury lamp	488: 473-491 & 506-534 561: 550-580 & 590-650 640: 590-650 & 663-738	oil immersion 63x Plan-Apochromat (1.4 NA)	12-bit AxioCam mRM	-	molecular combing imaging
Leica SP5 II/Wetzlar, Germany	405 nm diode 488 nm Argon, 561 nm DPSS, 633 nm HeNe gas	AOBS beam splitter	HCX PL APO 63x / 1.4-0.6 oil lambda blue & HCX PL APO 100x / 1.44 oil Corr CS	2 HyD Hybrid Detectors	-	confocal z-stack imaging
DeltaVision OMX V3/ GE, Chicago, IL, USA	405, 488 and 593 nm diode lasers	405: 401-447, 488: 500-550, 594: 603-627	100x 1.4 oil immersion objective UPlanSApo (Olympus)	Cascade II:512 EMCCD cameras (Photometrics)	-	3D SIM imaging

* ex.: excitation & em.: emission, ** dichroic specification, *** WD: working distance.

Supplementary Table 6: Genome-wide replication origin profiling information.

Dataset	Sample	Cells	Sequencing method	Webpage	Reference
GSE126477	-	mES	Small nascent DNA strand sequencing (SNS-seq)	https://www.ncbi.nlm.nih.gov/geo/query/acc.cgi?acc=GSE126477	(9)
	GSM3602315			https://www.ncbi.nlm.nih.gov/geo/query/acc.cgi?acc=GSM3602315	
	GSM3602316			https://www.ncbi.nlm.nih.gov/geo/query/acc.cgi?acc=GSM3602316	
	GSM3602317			https://www.ncbi.nlm.nih.gov/geo/query/acc.cgi?acc=GSM3602317	
GSE99740	-	mES	Small nascent DNA strand sequencing (SNS-seq)	https://www.ncbi.nlm.nih.gov/geo/query/acc.cgi?acc=GSE99740	(10)
	GSM2651111			https://www.ncbi.nlm.nih.gov/geo/query/acc.cgi?acc=GSM2651111	
	GSM2651112			https://www.ncbi.nlm.nih.gov/geo/query/acc.cgi?acc=GSM2651112	
GSE99740	-	MEF	Small nascent DNA strand sequencing (SNS-seq)	https://www.ncbi.nlm.nih.gov/geo/query/acc.cgi?acc=GSE99740	(10)
	GSM2651107			https://www.ncbi.nlm.nih.gov/geo/query/acc.cgi?acc=GSM2651107	
	GSM2651108			https://www.ncbi.nlm.nih.gov/geo/query/acc.cgi?acc=GSM2651108	
GSE116321	-	activated mouse B cells	Okazaki fragment sequencing (OK-seq)	https://www.ncbi.nlm.nih.gov/geo/query/acc.cgi?acc=GSE116321	(11)
	GSM3227970			https://www.ncbi.nlm.nih.gov/geo/query/acc.cgi?acc=GSM3227970	
	GSM3227971			https://www.ncbi.nlm.nih.gov/geo/query/acc.cgi?acc=GSM3227971	
	GSM3227972			https://www.ncbi.nlm.nih.gov/geo/query/acc.cgi?acc=GSM3227972	

Supplementary Table 7: Statistic parameters for Figure 1A, S8D and S12.

Figure	Stage	N	Mean	Median	SD	SEM	CI	*P*-value
**Live cell (1A & S8D)**	I	15	3.6	-	0.6	-	-	-
	II	23	4.6	-	0.9	-	-	-
	III	13	1.5	-	0.4	-	-	-
	IV	16	1.1	-	0.2	-	-	-
	IV$_{?}$	18	1.0	-	0.1	-	-	-
**RFi distance from nuclear border (S12)**	I	32	0.55	0.55	0.13	0.02	0.001	-
	II	32	0.35	0.35	0.08	0.01	0.001	8.2e-10
	III	29	0.3	0.3	0.1	0.02	0.001	4e-12
	IV	24	0.28	0.28	0.07	0.02	0.001	3.4e-13
	IV$_{?}$	27	0.33	0.28	0.1	0.02	0.001	1.8e-09
**RFi solidity (S12)**	I	32	0.65	0.66	0.01	0.002	0.0001	-/5.2e-13
	II	32	0.66	0.66	0.01	0.003	0.0002	0.49/7.3e-13
	III	29	0.67	0.67	0.02	0.004	0.0003	0.002/4.6e-10
	IV	24	0.69	0.69	0.02	0.005	0.0003	8.5e-08/1.9e-05
	IV$_{?}$	27	0.73	0.73	0.03	0.006	0.0004	5.2e-13/-
**RFi numbers (S12)**	I	32	232	213	92.12	16.28	1.03	0.091/8.0e-10
	II	32	278	273	123.2	21.78	1.38	-/3.7e-10
	III	29	212	202	100.85	18.73	1.18	0.024/5.0e-07
	IV	24	188	194	87.4	17.84	1.13	0.002/1.4e-05
	IV$_{?}$	27	83	77	59.57	11.46	0.73	3.7e-10/-

Figure	Stage	N	Mean	Median	SD	SEM	CI	*P*-value
**norm. DAPI intensity in RFi (S12)**	I	32	0.34	0.33	0.05	0.01	0.0005	0.013
	II	32	0.37	0.37	0.05	0.01	0.0005	-
	III	29	0.34	0.34	0.06	0.01	0.0007	0.012
	IV	24	0.32	0.32	0.06	0.01	0.0007	0.002
	$IV_{?}$	27	0.36	0.35	0.07	0.01	0.0008	0.47

Supplementary Table 8: Statistic parameters for Figure 2 and 3.

Figure	Days/stage/ celltype	N	Mean	Median	SD	SEM	CI	*P*-value
**Oct3/4 (2C)**	0	107	1.41	1	1.19	0.115	0.0072	-
	3	117	0.2	0.2	0.07	0.007	0.0004	< 2.16e-16
	7	102	0.14	0.14	0.05	0.005	0.0003	< 2.16e-16
**Sox2 (2C)**	0	37	1.01	1	0.11	0.02	0.0011	-
	3	33	0.54	0.53	0.02	0.004	0.0002	< 2.16e-16
	7	38	0.53	0.53	0.03	0.005	0.00003	< 2.16e-16
**EdU (2E)**	early	12	480301	342456	45857	3309	2078	-
	mid	16	685792	422070	68445	3392	2129	0.002417
	late	13	847384	551027	106878	11329	7124	1.74e-05
**H3K9ac (2H)**	undiff. mESC	24	0.74	0.74	0.18	0.028	0.055	-
	diff. mESC	28	0.61	0.64	0.09	0.017	0.034	0.00054
	primary	25	0.51	0.48	0.11	0.021	0.042	2.23e-07
**H3K9m3 (2H)**	undiff. mESC	20	1.93	1.99	0.36	0.081	0.159	-
	diff. mESC	25	2.33	2.12	0.74	0.149	0.292	0.1221
	primary	19	2.77	2.52	0.64	0.147	0.288	1.627e-05
**H4K8ac (2H)**	undiff. mESC	26	0.86	0.78	0.16	0.032	0.062	-
	diff. mESC	26	0.63	0.64	0.11	0.021	0.043	0.000995
	primary	17	0.5	0.48	0.08	0.018	0.036	5.864e-06
**H4K5ac (3B)**	control	28	1	-	0.25	-	-	-
	HDAC1	34	0.4	-	0.16	-	-	< 2.16e-16
**H4K8ac (3B)**	control	21	1	-	0.25	-	-	-
	HDAC1	28	0.2	-	0.04	-	-	< 2.16e-16

Figure	Days/stage/ celltype	N	Mean	Median	SD	SEM	CI	*P*-value
**II to III (3D)**	control	28	100	-	1.14	-	-	-
	HDAC1	16	51.6	-	9.3	-	-	-
**‘III’ to ‘II’ (3D)**	control	0	0	-	0	-	-	-
	HDAC1	15	48.4	-	7.4	-	-	-
**Chromocenter volume (3E)**	control	13	14.13	6.24	17.82	2.09	0.13	-
	HDAC1 II to III	10	10.31	6.86	15.07	1.53	0.1	0.14
	HDAC1 ’III’ to ‘II’	9	5.88	4.36	6.43	0.6	0.04	0.0003
**Chromocenter shape (3F)**	control	13	0.54	0.56	0.12	0.01	0.001	-
	HDAC1 II to III	10	0.6	0.61	0.07	0.01	0.0004	9.4e-10
	HDAC1 ’III’ to ‘II’	9	0.66	0.67	0.07	0.01	0.0004	6.3e-12

Supplementary Table 9: Statistic parameters for Figure S14.

Figure	Celltype	N	Mean	Median	SD	SEM	CI	*P*-value
**Number of chromocenters (S14B)**	undiff. mESC	28	11.15	10.00	5.54	1.24	0.080	-
	diff. mESC	28	22.00	20.50	7.70	1.72	0.110	1.17e-05
	primary	31	23.35	22.00	9.76	2.18	0.140	3.42e-05
**Chromocenter volume (S14C)**	undiff. mESC	28	6.88	4.80	9.38	0.63	0.04	-
	diff. mESC	28	3.50	2.05	4.13	0.20	0.01	5.25e-07
	primary	31	3.60	2.07	4.68	0.22	0.01	1.31e-06
**Chromocenter compaction (S14D)**	undiff. mESC	28	0.04	0.037	0.02	0.0012	0.00007	-
	diff. mESC	28	0.05	0.05	0.04	0.0015	0.00009	< 2.16e-16
	primary	31	0.06	0.051	0.03	0.0013	0.00008	< 2.16e-16
**Chromocenter shape factor (S14E)**	undiff. mESC	28	0.48	0.47	0.10	0.006	0.004	-
	diff. mESC	28	0.56	0.56	0.08	0.004	0.002	< 2.16e-16
	primary	31	0.58	0.58	0.07	0.003	0.002	< 2.16e-16

Supplementary Table 10: Statistic parameters for Figure 4, S16, S18 and S19.

Figure	Stage	N	Mean	Median	SD	SEM	CI	*P*-value
**MaSat/MiSat/ telomeres (4B)**	I	12	27.26/ 29.22/ 34.39	25.97/ 27.5/ 33.94	4.4/ 5.15/ 4.47	1.27/ 1.49/ 1.29	0.08/ 0.1/ .08	-
	II	11	43.13/ 38.03/ 38.44	41.37/ 39.05/ 40.39	5.16/ 5.14/ 5.37	1.56/ 1.55/ 1.62	0.1/ 0.1/ 0.1	1.5e-07/ 0.0005/ 0.06
	III	12	21.59/ 26.36/ 27.21	20.79/ 26.46/ 28.3	6.14/ 5.47/ 5.81	1.77/ 1.58/ 1.68	0.11/ 0.1/ 0.11	0.02/ 0.2/ 0.003
	IV	10	14.97/ 17.25/ 21.05	15.01/ 17.6/ 20.69	4.11/ 3.63/ 4.99	1.3/ 1.15/ 1.58	0.08/ 0.07/ 0.1	1.5e-06/ 3.6e-06/ 3.4e-06
	Y	10	9.3/ 10.87/ 11.26	8.41/ 9.49/ 9.75	3.04/ 4.35/ 3.8	0.96/ 1.38/ 1.2	0.06/ 0.09/ 0.08	5.7e-10/ 1.6e-08/ 2.8e-11
**Y chromosome (4C & S16D)**	I	13	273.67	208.8	265.82	23.97	1.51	4.8e-12
	II	13	278.99	185.16	265.11	36.08	2.27	3.6e-12
	III	8	302.93	158.63	357.69	103.26	6.62	2.2e-11
	IV	8	323.07	53.94	537.48	155.16	9.95	6.6e-9
	Y	10	2049.87	2077.22	849.74	157.79	9.98	-
**Telomeres in vs out (4D)**	in	14	4014	2951	3725	235	14.73	-
	out	14	2172	1429	2231	111	6.97	6.77e-12
**MaSat (S16A)**	I	19	234.39	175.48	225.9	14.9	0.94	-
	II	19	896.88	857.6	492.27	36.79	2.3	< 2.16e-16
	III	5	229.56	173.97	216.2	27.24	1.72	0.8766
	IV	6	82.89	43.93	106.67	11.37	0.72	1.51e-14
	Y	6	79.33	63.19	70.1	12.59	0.8	6.07e-13

Figure	Stage	N	Mean	Median	SD	SEM	CI	*P*-value
MiSat (S16B)	I	13	15.87	13.67	12.48	0.83	0.052	-
	II	16	28.13	24.22	20.79	1.51	0.072	< 2.16e-16
	III	13	7.1	4.31	7.69	0.45	0.028	< 2.16e-16
	IV	10	3.41	2.22	4.04	0.27	0.017	< 2.16e-16
	Y	5	3.21	1.26	5.28	0.54	0.034	< 2.16e-16
Telomeres (S16C)	I	14	28.4	25.63	17.33	0.72	0.045	-
	II	11	33.8	25.64	28.02	1.27	0.080	0.00023
	III	10	29.53	25.74	20.65	1.11	0.070	0.392
	IV	11	18.07	12.94	16.99	1.03	0.065	1.32e-15
	Y	10	11.07	6.7	14.15	1.03	0.064	< 2.16e-16
Y/MaSat/ telomeres (S18A)	I	8	21.4/<br>24.19/<br>27.4	22.05/<br>23.85/<br>27.95	2.43/<br>2.33/<br>2.96	0.86/<br>0.82/<br>1.05	0.06/<br>0.05/<br>0.07	-
	II	8	25.31/<br>44.54/<br>32.56	24.59/<br>44.92/<br>31.97	5.17/<br>2.43/<br>3.44	1.83/<br>0.86/<br>1.22	0.12/<br>0.06/<br>0.08	0.08/<br>9.1e-11/<br>0.006
	III	8	23.01/<br>21.22/<br>21.79	22.58/<br>22.41/<br>23.41	5.23/<br>5.43/<br>5.58	1.85/<br>1.92/<br>1.97	0.12/<br>0.12/<br>0.11	0.45/<br>0.19/<br>0.03
	IV	8	22.9/<br>13.23/<br>16.93	23.36/<br>13.29/<br>16.25	3.57/<br>2.82/<br>4.85	1.26/<br>1.0/<br>1.71	0.08/<br>0.06/<br>0.11	0.34/<br>9e-07/<br>0.0002
	Y	9	50.67/<br>7.22/<br>9.13	50.05/<br>6.88/<br>9.53	5.92/<br>1.32/<br>2.42	1.97/<br>0.44/<br>0.81	0.13/<br>0.03/<br>0.05	3.6e-08/<br>1.9e-09/<br>2.2e-09

Figure	Stage	N	Mean	Median	SD	SEM	CI	*P*-value
**Y/MaSat/ MiSat (S18B)**	I	9	18.93/<br>26.99/<br>25.97	16.46/<br>24.95/<br>25.84	10.43/<br>3.88/<br>4.73	3.48/<br>1.29/<br>1.58	0.23/<br>0.08/<br>0.1	-
	II	11	20.3/<br>48.37/<br>41.08	18.61/<br>48.39/<br>42.05	8.87/<br>7.18/<br>6.74	2.67/<br>2.16/<br>2.03	0.17/<br>0.14/<br>0.13	0.76/<br>2.7e-07/<br>1.6e-05
	III	9	20.03/<br>20.49/<br>21.45	17.17/<br>19.2/<br>22.14	8.51/<br>5.32/<br>3.75	2.84/<br>1.77/<br>1.25	0.18/<br>0.09/<br>0.12	0.8/<br>0.01/<br>0.04
	IV	10	31.32/<br>19.17<br>21.46	33.16/<br>19.29/<br>20.96	5.595/<br>4.52/<br>6.06	1.88/<br>1.43/<br>1.92	0.12/<br>0.09/<br>0.12	0.008/<br>0.0008/<br>0.09
	Y	9	51.04/<br>10.27/<br>10.68	53.52/<br>11.07/<br>10.87	9.45/<br>4.43/<br>6.23	3.15/<br>1.48/<br>2.08	0.2/<br>0.1/<br>0.13	4.2e-06/<br>2.7e-07/<br>3.2e-05
**Y/MiSat/ telomeres (S18C)**	I	9	21.26/<br>21.13/<br>26.72	20.22/<br>20.73/<br>25.67	2.97/<br>3.54/<br>3.57	0.99/<br>1.18/<br>1.19	0.06/<br>0.08/<br>0.08	-
	II	8	23.14/<br>38.49/<br>34.38	23.45/<br>38.96/<br>34.41	4.49/<br>3.28/<br>1.76	1.59/<br>1.16/<br>0.62	0.1/<br>0.08/<br>0.04	0.33/<br>2.7e-08/<br>0.0001
	III	7	22.87/<br>26.79/<br>26.45	22.41/<br>26.39/<br>27.51	2.96/<br>2.72/<br>2.8	1.12/<br>1.02/<br>1.06	0.07/<br>0.07/<br>0.07	0.3/<br>0.003/<br>0.9
	IV	9	24.01/<br>20.32/<br>19.27	23.92/<br>20.78/<br>19.34	6.17/<br>2.12/<br>1.8	2.06/<br>0.71/<br>0.6	0.13/<br>0.05/<br>0.04	0.3/<br>0.7/<br>0.0001
	Y	10	51.91/<br>14.27/<br>13.29	50.75/<br>12.64/<br>12.47	5.73/<br>3.61/<br>2.63	1.81/<br>1.14/<br>0.83	0.12/<br>0.07/<br>0.05	7e-10/<br>0.0006/<br>1.7e-07
**Y chromosome MEF W8 (S19A)**	early	50	285.24	125.95	413.16	58.43	3.68	< 2.16e-16
	mid	10	401.8	284.74	399.81	126.43	8.15	1.7e-07
	late	55	413.38	345.17	236.73	31.92	2.01	< 2.16e-16
	Y	83	1581.58	1323.29	1013.69	111.22	7.00	-

Supplementary Table 11: Statistic parameters for Figure 1C and 5.

Stage	N	Mean	SD
I	368	24.7	0.6
II	458	30.7	0.85
III	239	15.5	0.5
Y	91	6.1	0.1
non S	356	22.9	0.8

Supplementary Table 12: Statistic parameters for Figure 6.

Figure	Substage	N	Mean	Median	SD	SEM	CI	*P*-value
**# RFi (6B)**	I	24	3182	3160	557	114	7.2	-
	II	28	3567	3398	542	102	6.5	-
	III	36	3217	3248	526	88	5.5	-
	Y	7	1881	1607	831	314	314	-
**nanoRFi/pWF RFi (6D)**	C2C12	16	5.99	-	0.42	0.11	0.23	-
	mES cell	30	6.5	-	0.39	0.12	0.26	0.0034
**Clustered nanoRFi per pWF (6D)**	C2C12	16	3.82	3	2.3	0.021	0.0014	-
	mES cell	30	4.01	3	2.6	0.027	0.0017	2.52e-08
**Volume pWF RFi (6E)**	C2C12	16	103.9	82.4	88.4	0.87	0.0054	-
	mES cell	30	121.6	71.6	115.6	1.07	0.0067	< 2.16e-16
**Volume nanoRFi (6F)**	C2C12	16	12.4	9	115.1	0.0046	0.0009	-
	mES cell	30	13.3	9.6	114.2	0.0049	0.0003	0.75

Supplementary Table 13: Statistic parameters for Figure 7, Supplementary Figure S21 and S22.

Figure	N	Mean	Median	SD	SEM	CI	*P*-value
**mESC (7E)**	71111	33.62	21.02	44.88	0.17	0.011	-
**MEF (7E)**	34159	69.93	44.96	85.21	0.46	0.029	< 2.16e-16
**J1 karyotype (7H)**	143	37.18	39	3.98	0.33	0.02	-
**mESC (X chr) (S21A)**	2645	62.23	37.17	188.49	3.67	0.23	-
**MEF (X chr) (S21A)**	1998	82.36	49.2	235.93	5.28	0.33	0.0017
**mESC (autos.) (S21A)**	71111	33.62	21.02	44.88	0.17	0.011	-
**MEF (autos.) (S21A)**	34159	69.93	44.96	85.21	0.46	0.029	< 2.16e-16
**mESC (S22A)**	71257	709.18	596	327.15	1.23	0.077	-
**MEF (S22A)**	34198	621.86	569	209.75	1.13	0.071	-
**act. B cells (S22A)**	8897	21415.53	17000	13618.36	144.38	9.054	-
**mESC (S22B)**	33746	70.85	58.89	54.52	0.3	0.019	-
**MEF (S22B)**	23843	100.19	75.07	90.57	0.59	0.037	< 2.16e-16
**act. B cells (S22B)**	8878	266.21	129	583.6	6.19	0.0388	< 2.16e-16

SUPPLEMENTARY FIGURES

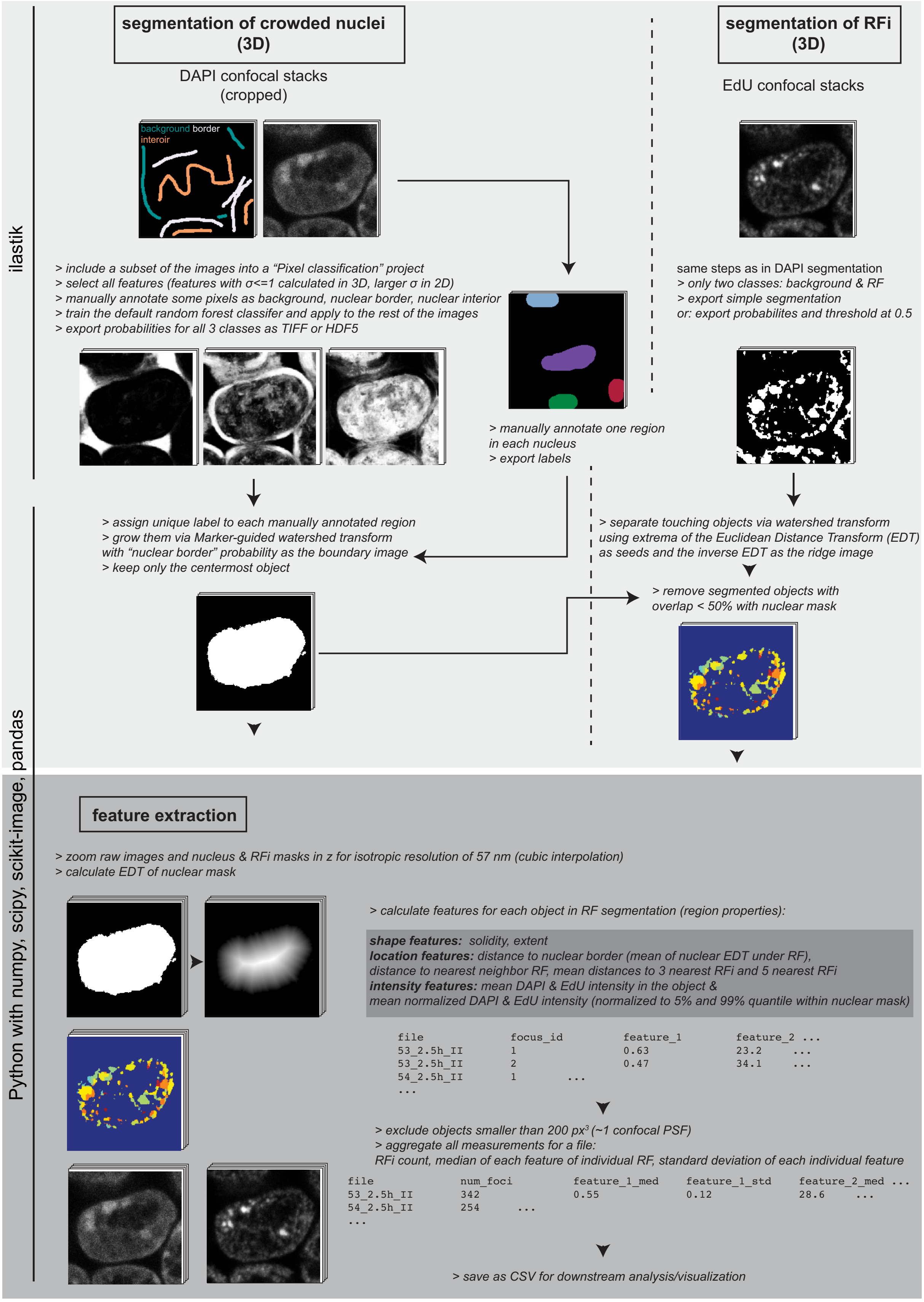

Supplementary Figure S1 – Analysis pipeline of replication foci (RFi) features. To extract quantitative features of RFi, DAPI stained nuclei and EdU labeled RFi were segmented in confocal stacks of mES cells. The central nucleus as well as RFi were segmented using supervised pixel classification (ilastik) and watershed transform (Python with scipy/scikit-image). Several features were calculated for each RF and used to create an aggregate feature vector for each image. A detailed analysis pipeline is described in the Materials and Methods section.

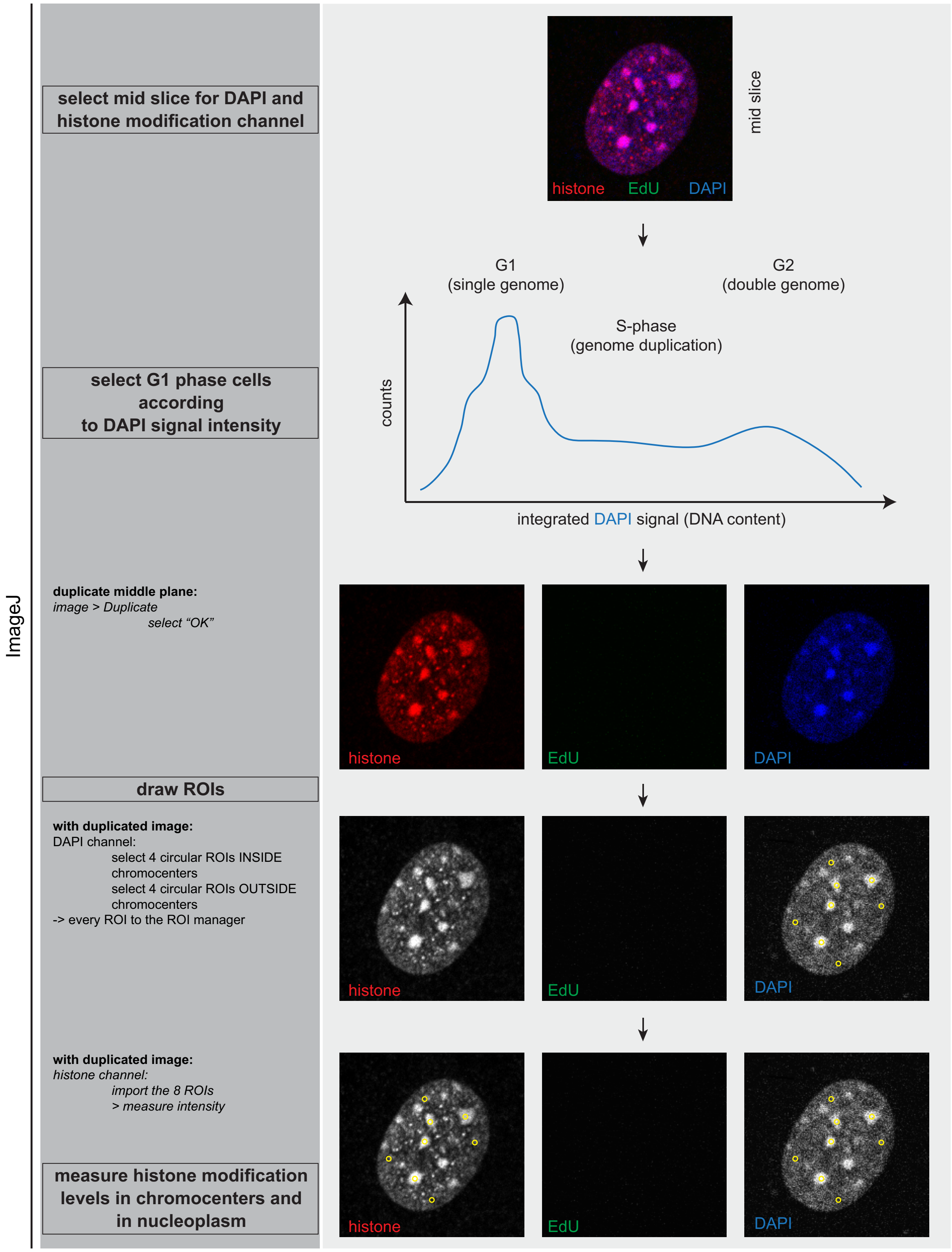
ImageJ
select mid slice for DAPI and histone modification channel
mid slice
histone
EdU
DAPI
select G1 phase cells according to DAPI signal intensity
G1
(single genome)
G2
(double genome)
S-phase
(genome duplication)
counts
integrated DAPI signal (DNA content)
duplicate middle plane:
image > Duplicate
select "OK"
histone
EdU
DAPI
draw ROIs
with duplicated image:
DAPI channel:
select 4 circular ROIs INSIDE chromocenters
select 4 circular ROIs OUTSIDE chromocenters
-> every ROI to the ROI manager
histone
EdU
DAPI
with duplicated image:
histone channel:
import the 8 ROIs
> measure intensity
measure histone modification levels in chromocenters and in nucleoplasm
histone
EdU
DAPI

Supplementary Figure S2 – Analysis pipeline of histone modification levels. To determine the histone modification levels at chromocenters, DAPI intensities of all cells were measured at middle planes and cells were grouped into G1, S and G2 phases, accordingly. Four circular regions of interest (ROI) were drawn in middle plane G1 phase cells inside DAPI intense stained chromocenters. Similarly, four circular ROIs were drawn outside of chromocenters and intensity levels within these ROIs were measured in the histone modification channel. A detailed analysis pipeline is described in the Materials and Methods section.

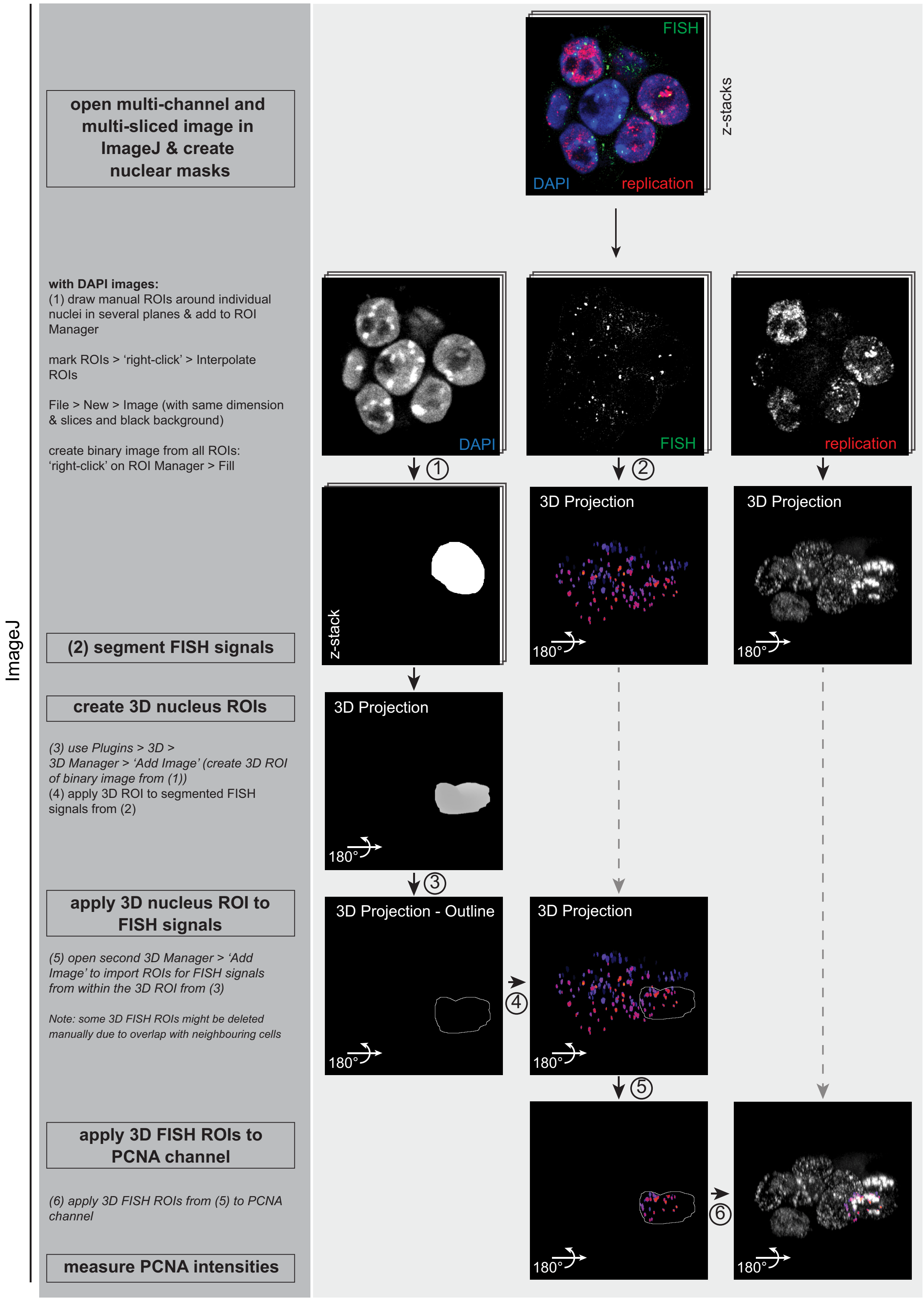
ImageJ
open multi-channel and multi-sliced image in ImageJ & create nuclear masks
with DAPI images:
(1) draw manual ROIs around individual nuclei in several planes & add to ROI Manager
mark ROIs > 'right-click' > Interpolate ROIs
File > New > Image (with same dimension & slices and black background)
create binary image from all ROIs: 'right-click' on ROI Manager > Fill
(2) segment FISH signals
create 3D nucleus ROIs
(3) use Plugins > 3D > 3D Manager > 'Add Image' (create 3D ROI of binary image from (1))
(4) apply 3D ROI to segmented FISH signals from (2)
apply 3D nucleus ROI to FISH signals
(5) open second 3D Manager > 'Add Image' to import ROIs for FISH signals from within the 3D ROI from (3)
Note: some 3D FISH ROIs might be deleted manually due to overlap with neighbouring cells
apply 3D FISH ROIs to PCNA channel
(6) apply 3D FISH ROIs from (5) to PCNA channel
measure PCNA intensities
FISH
DAPI
replication
z-stacks
DAPI
FISH
replication
1
2
3D Projection
3D Projection
z-stack
180°
180°
3D Projection
180°
3
4
3D Projection - Outline
3D Projection
180°
180°
5
6
180°
180°

Supplementary Figure S3 – Analysis pipeline of Repli-FISH analysis. To determine the replication timing of specific (sub)chromosomal elements, 3D masks of individual cell nuclei were manually generated based on the DAPI channel. FISH signals were segmented. The 3D nucleus mask was applied to the segmented FISH signals and the generated nuclear FISH ROIs were used to mask the PCNA signals and PCNA intensities within the mask were measured. A detailed analysis pipeline is described in the Materials and Methods section.

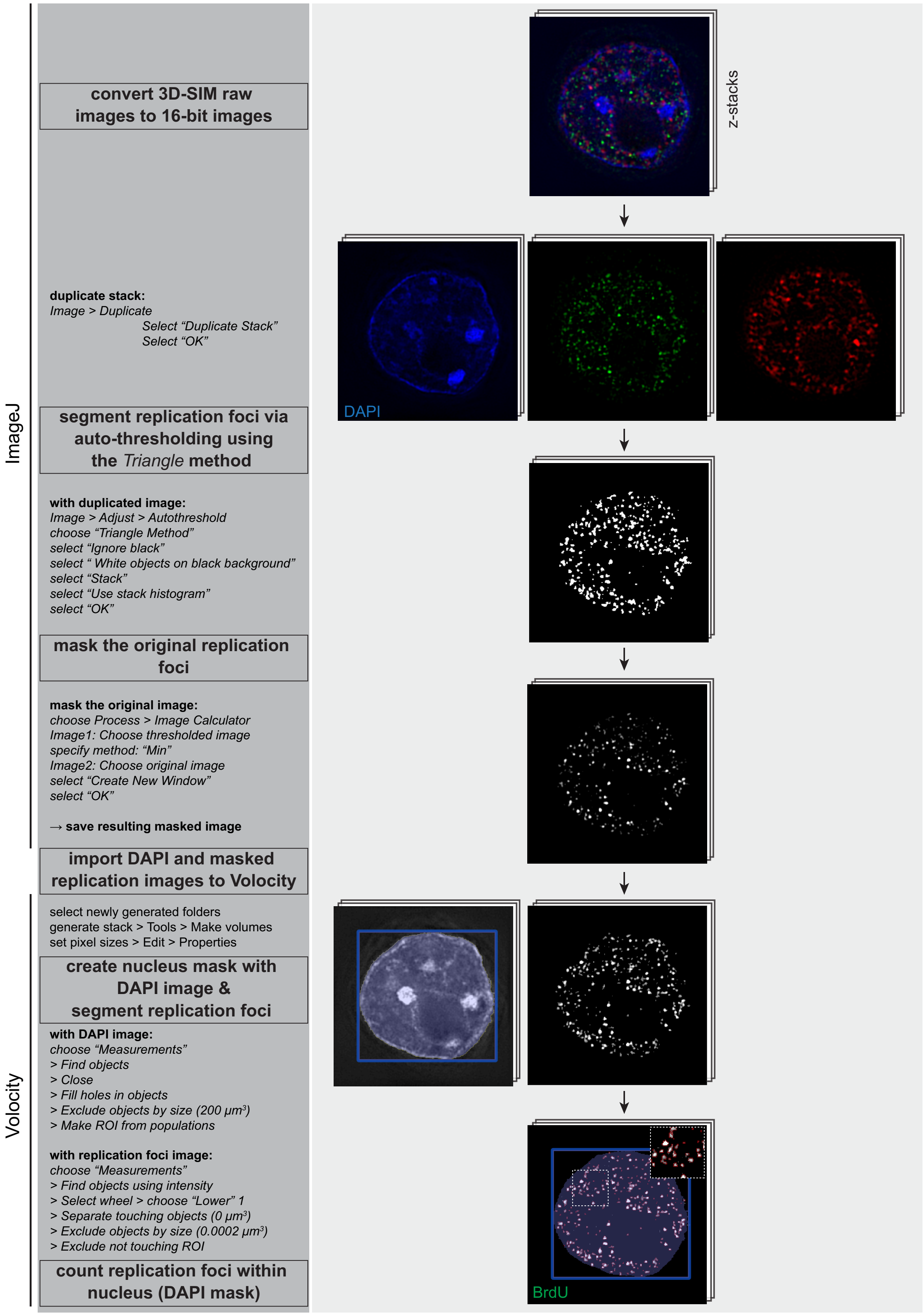
ImageJ
Volocity
convert 3D-SIM raw images to 16-bit images
z-stacks
duplicate stack:
Image > Duplicate
Select "Duplicate Stack"
Select "OK"
DAPI
segment replication foci via auto-thresholding using the Triangle method
with duplicated image:
Image > Adjust > Autothreshold
choose "Triangle Method"
select "Ignore black"
select " White objects on black background"
select "Stack"
select "Use stack histogram"
select "OK"
mask the original replication foci
mask the original image:
choose Process > Image Calculator
Image1: Choose thresholded image
specify method: "Min"
Image2: Choose original image
select "Create New Window"
select "OK"
→ save resulting masked image
import DAPI and masked replication images to Volocity
select newly generated folders
generate stack > Tools > Make volumes
set pixel sizes > Edit > Properties
create nucleus mask with DAPI image & segment replication foci
with DAPI image:
choose "Measurements"
> Find objects
> Close
> Fill holes in objects
> Exclude objects by size (200 µm³)
> Make ROI from populations
with replication foci image:
choose "Measurements"
> Find objects using intensity
> Select wheel > choose "Lower" 1
> Separate touching objects (0 µm³)
> Exclude objects by size (0.0002 µm³)
> Exclude not touching ROI
count replication foci within nucleus (DAPI mask)
BrdU

Supplementary Figure S4 – Analysis pipeline of 3D structured illumination microscopy (3D-SIM) resolved nano replication foci counting. For counting of nano replication foci (nanoRFi) in 3D-SIM images, analysis was done using ImageJ and the PerkinElmer Volocity software. Raw image files were converted to 16-bit images, individual cell nuclei were segmented based on the DAPI channel, replication signals were thresholded and the binary images were used to mask the original replication foci signals. DAPI and masked replication foci images were imported to Volocity to quantify replication foci for individual nuclei by creating nuclear masks and corresponding ROIs (regions of interest) were based on the DAPI images. Next, 3D-SIM replication foci were detected by intensity and only foci within the nuclear ROI were counted. A detailed analysis pipeline is described in the Materials and Methods section.

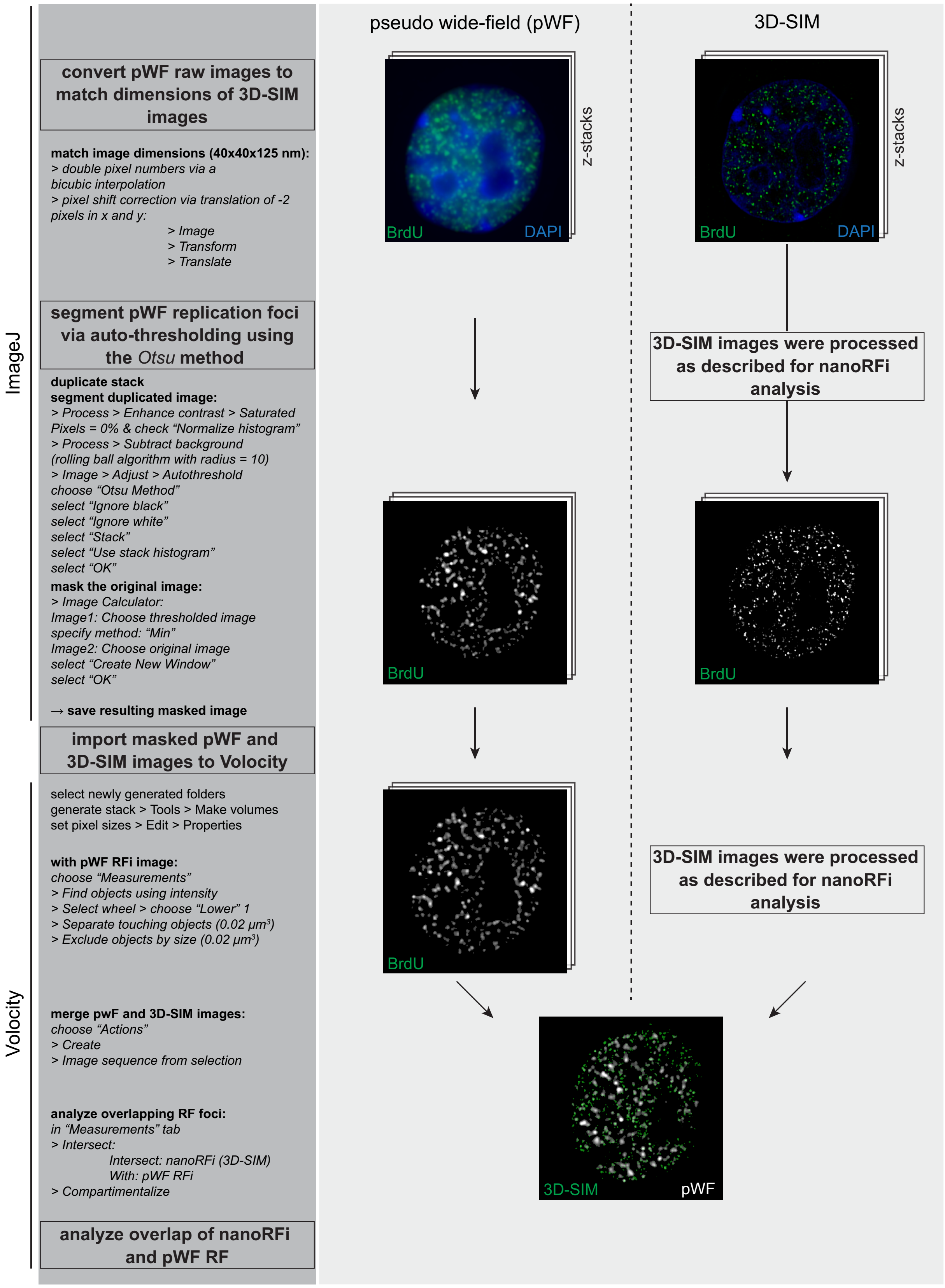
ImageJ
Volocity
pseudo wide-field (pWF)
3D-SIM
convert pWF raw images to match dimensions of 3D-SIM images
match image dimensions (40x40x125 nm):
> double pixel numbers via a bicubic interpolation
> pixel shift correction via translation of -2 pixels in x and y:
> Image
> Transform
> Translate
segment pWF replication foci via auto-thresholding using the Otsu method
duplicate stack
segment duplicated image:
> Process > Enhance contrast > Saturated Pixels = 0% & check "Normalize histogram"
> Process > Subtract background
(rolling ball algorithm with radius = 10)
> Image > Adjust > Autothreshold
choose "Otsu Method"
select "Ignore black"
select "Ignore white"
select "Stack"
select "Use stack histogram"
select "OK"
mask the original image:
> Image Calculator:
Image1: Choose thresholded image
specify method: "Min"
Image2: Choose original image
select "Create New Window"
select "OK"
→ save resulting masked image
import masked pWF and 3D-SIM images to Volocity
select newly generated folders
generate stack > Tools > Make volumes
set pixel sizes > Edit > Properties
with pWF RFi image:
choose "Measurements"
> Find objects using intensity
> Select wheel > choose "Lower" 1
> Separate touching objects (0.02 µm³)
> Exclude objects by size (0.02 µm³)
merge pwF and 3D-SIM images:
choose "Actions"
> Create
> Image sequence from selection
analyze overlapping RF foci:
in "Measurements" tab
> Intersect:
Intersect: nanoRFi (3D-SIM)
With: pWF RFi
> Compartimentalize
analyze overlap of nanoRFi and pWF RF
z-stacks
BrdU
DAPI
3D-SIM images were processed as described for nanoRFi analysis
3D-SIM
pWF

Supplementary Figure S5 – Analysis pipeline of nano replication foci per pseudo-widefield replication focus quantification. For counting nano replication foci (nanoRFi) per pseudo-widefield (pWF) replication focus, analysis was done using ImageJ and the PerkinElmer Volocity software. Dimensions of pWF replication raw images were matched to the image dimension of the 3D-SIM data (40 x 40 x 125 nm). Individual cell nuclei were segmented based on the DAPI channel, histograms of replication signals were normalized, background was subtracted, signals were thresholded and the binary images were used to mask the original replication foci signals. 3D-SIM replication signals were processed as described in Supplementary Figure S4. Segmented and masked pseudoWF and 3D-SIM image stacks were imported to Volocity and merged. Detection of pWF RFi was based on intensity as for 3D-SIM images and the number of nanoRFi contained in one pWF RF were counted.

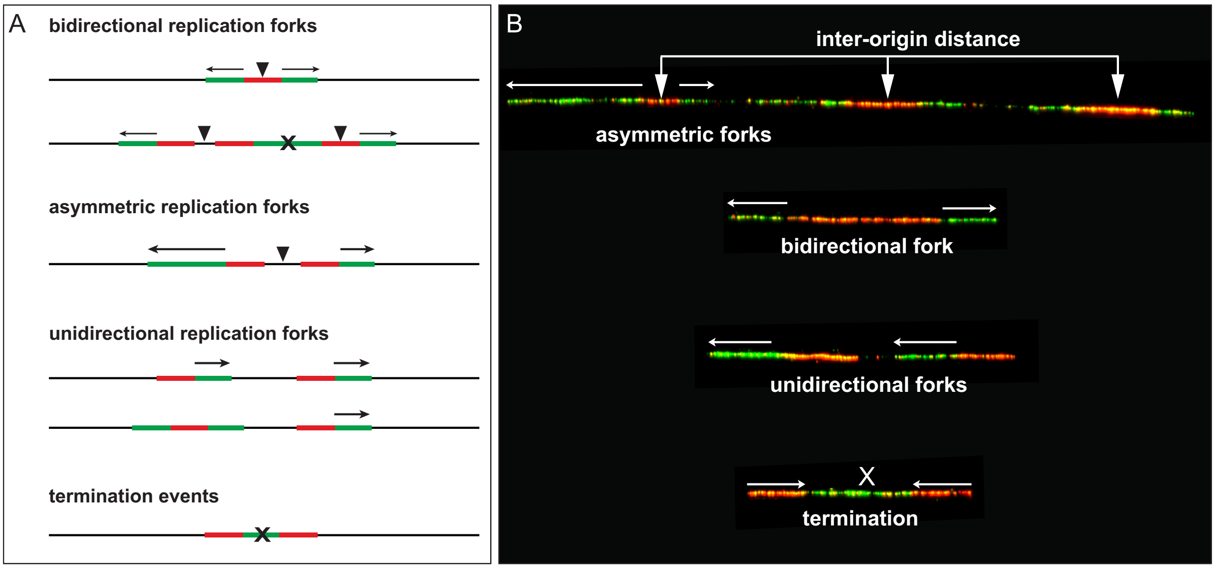

Supplementary Figure S6 – Analysis of the mouse embryonic stem cell replicon by DNA fiber assays. (A) Classification of typical DNA fiber signals obtained from molecular combing experiments. Replication fork speed is calculated from measurements of green replication signals (arrows) flanking red tracks that mark initiation events during the first pulse labeling period (origins of replication marked by black arrowheads). Merging forks within clusters of origins and termination events are discarded (marked with X). Fork asymmetry can be assessed by calculating the ratio of left/right replication forks, emanating from an origin of replication. Replication signals spreading in only one direction are considered as unidirectional replication forks. **(B)** Representative examples of replication signals from DNA combing experiments. Labeling is according to the classification described in (A). The inter-origin distance (IOD) is the distance between neighboring initiation sites within a cluster, identified based on the first nucleotide label (IdU, red).

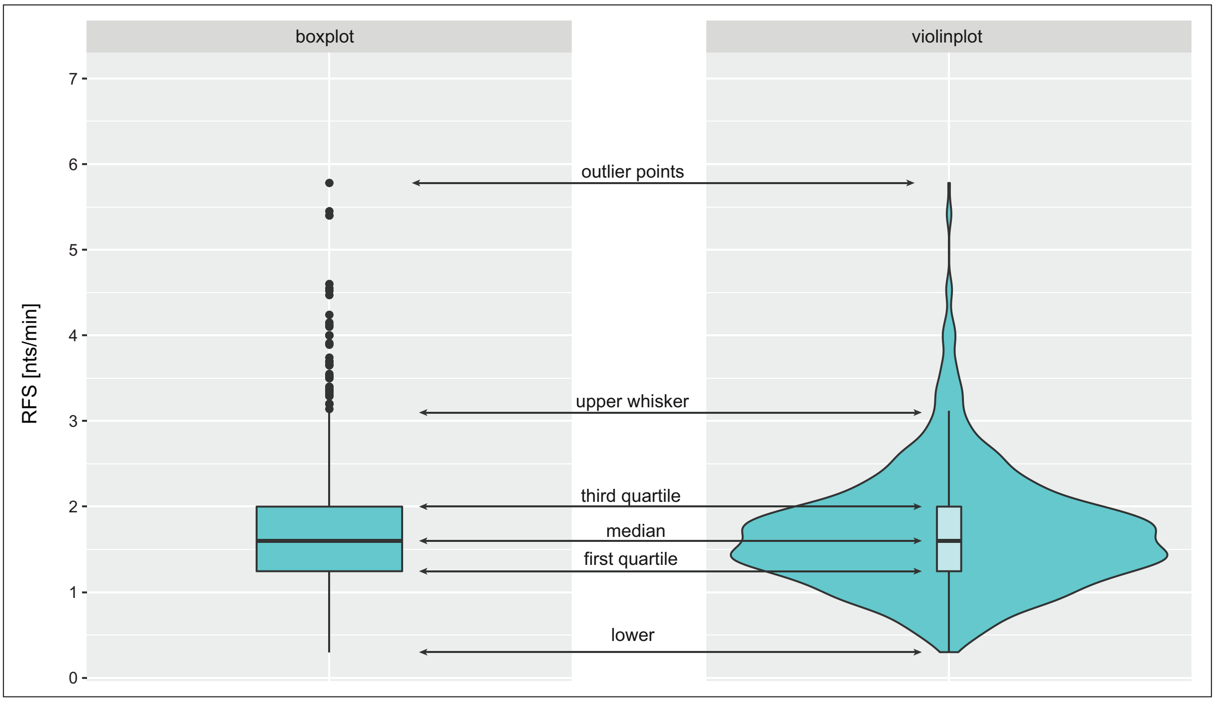

Supplementary Figure S7 – Statistics representation using violin plots. Statistical analyses were represented by violin plots, a variation of boxplots with a kernel density plot on each side. Similar to a boxplot, boxes and whiskers represent 25–75 percentiles and 1.5 times the IQD (inter-quartile distance), respectively and the center line depicts the median. Additionally, violin plots represent the probability density of the data at different values.

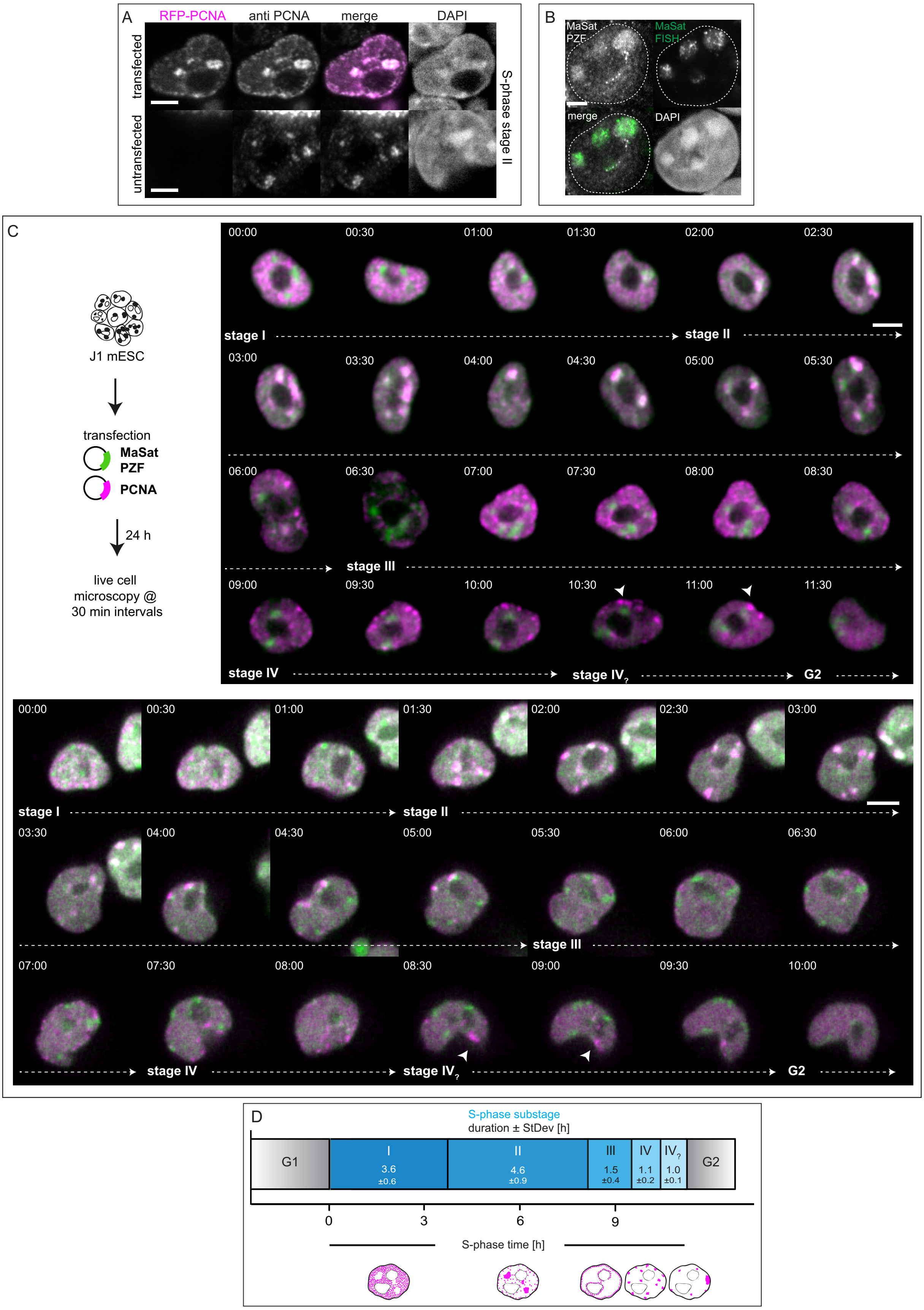
A
RFP-PCNA
anti PCNA
merge
DAPI
transfected
untransfected
S-phase stage II
B
MaSat PZF
MaSat FISH
merge
DAPI
C
J1 mESC
transfection
MaSat PZF
PCNA
24 h
live cell microscopy @ 30 min intervals
stage I
stage II
stage III
stage IV
stage IV?
G2
D
S-phase substage
duration ± StDev [h]
G1
I 3.6 ±0.6
II 4.6 ±0.9
III 1.5 ±0.4
IV 1.1 ±0.2
IV? 1.0 ±0.1
G2
0
3
6
9
S-phase time [h]

Supplementary Figure S8 – Live-cell analysis of DNA replication dynamics in mouse embryonic stem cells. (A) J1 mES cells were transfected with mRFP-PCNA and stained with a PCNA specific antibody. Representative confocal images of the anti-PCNA staining (grey) in an mRFP-PCNA (magenta) transfected and an untransfected cell are shown. **(B)** MaSat-GFP polydactyl zinc finger (PZF) transfected cells were fixed using a formaldehyde gradient with simultaneous permeabilization and major satellite repeat sequences were visualized by fluorescence *in situ* hybridization (FISH) with a MaSat specific probe (green). Representative confocal images are shown. **(C)** mES cells were co-transfected with plasmids encoding fusion constructs of mRFP-PCNA (magenta) and GFP-tagged polydactyl zinc finger protein binding to major satellite sequences (MaSat-GFP, green) to follow S-phase progression and to mark pericentromeric heterochromatin (chromocenters), respectively, and imaging was performed for 24 hours with 30 minutes intervals. Spinning disk confocal images of a representative cell progressing through S-phase (stage I to IV$_?$) and G2 phase. **(D)** Approximate durations of the individual substages (mean ± StDev) were calculated from at least 15 cells (Supplementary Table 7) from different live cell experiments. The arrowheads in the IV$_?$ stage mark a prominent accumulation of replication signals observed at the end of S-phase. Scale bar = 5 µm.

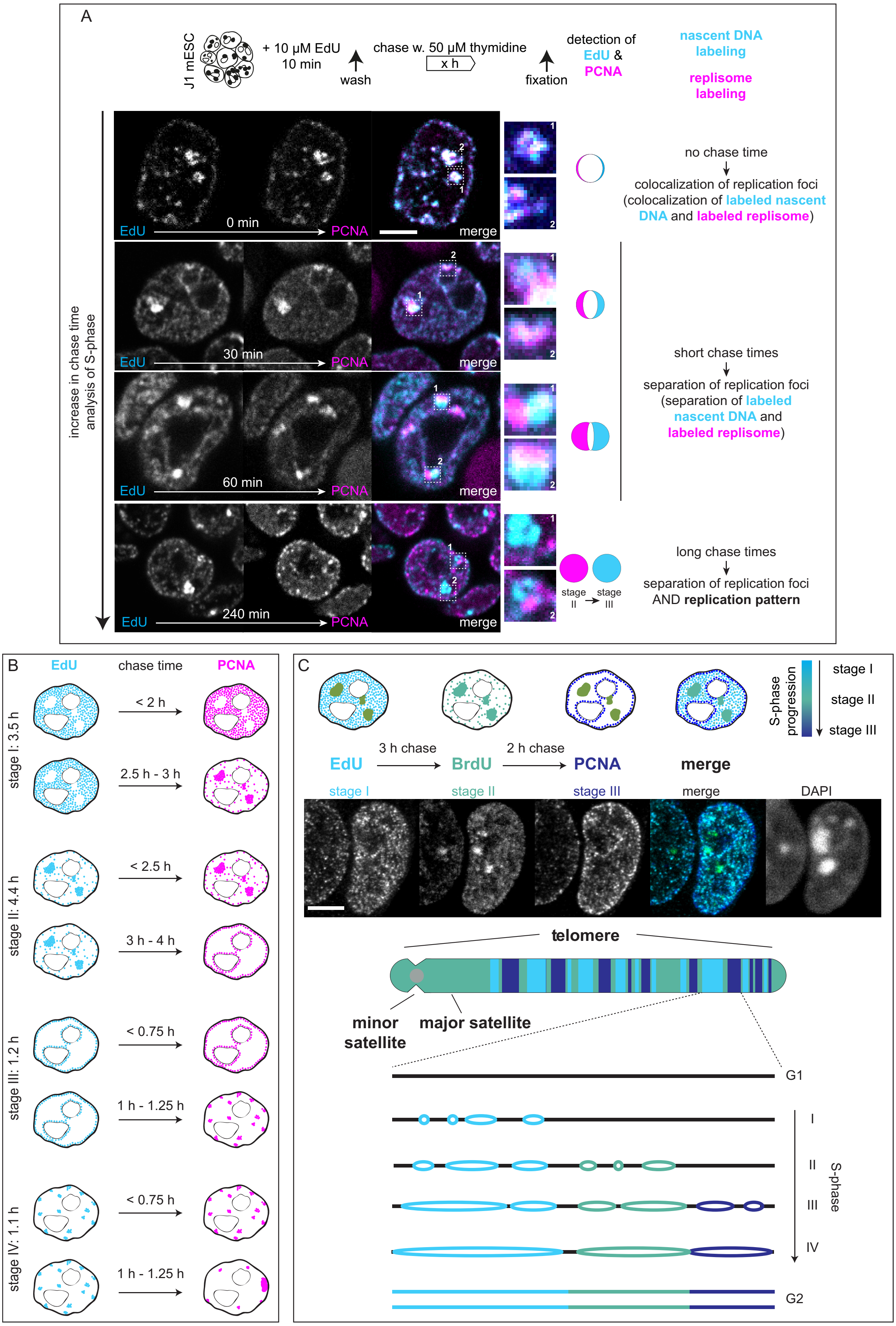
A
J1 mESC
+ 10 µM EdU
10 min
wash
chase w. 50 µM thymidine
x h
fixation
detection of
EdU &
PCNA
nascent DNA labeling
replisome labeling
increase in chase time
analysis of S-phase
EdU
0 min
PCNA
merge
30 min
60 min
240 min
no chase time
colocalization of replication foci
(colocalization of labeled nascent DNA and labeled replisome)
short chase times
separation of replication foci
(separation of labeled nascent DNA and labeled replisome)
long chase times
separation of replication foci
AND replication pattern
stage II
stage III
B
EdU
chase time
PCNA
stage I: 3.5 h
< 2 h
2.5 h - 3 h
stage II: 4.4 h
< 2.5 h
3 h - 4 h
stage III: 1.2 h
< 0.75 h
1 h - 1.25 h
stage IV: 1.1 h
< 0.75 h
1 h - 1.25 h
C
S-phase progression
stage I
stage II
stage III
3 h chase
2 h chase
EdU
BrdU
PCNA
merge
stage I
stage II
stage III
merge
DAPI
telomere
minor satellite
major satellite
G1
I
II
III
IV
S-phase
G2

Supplementary Figure S9 – Description of the pulse chase experiments. (A) Experimental setup of a pulse-chase experiment to determine the spatio-temporal progression of DNA replication in mES cells. Asynchronously growing mES cell cultures were pulse labeled with the nucleotide analog EdU, followed by various thymidine chase periods (white arrows) and fixation. Subsequently, replication foci marked by the incorporated EdU, i.e. nascent DNA during the first pulse labeling (cyan), and the replisome component PCNA, i.e. ongoing replication at the time point of fixation (magenta), were (immuno)fluorescently detected (Figure 1C). The different chase times resulted in different degrees of replication foci separation and, with increasing chase times, eventually also to transition from one S-phase pattern to the next. Representative confocal images of EdU pulsed cells followed by different chase times before fixation, are shown. **(B)** Schematic overview of the S-phase progression with different chase durations. Durations of the different S-phase stages are indicated on the left. Chase durations in the fixed cell approach were changed according to S-phase substage duration. While shorter chase times (< 2 hours/ 2.5 hours for stage I - II and II - III, respectively and < 0.75 hours for stage III - IV and IV - $IV_?$ transitions) do not visualize substage transitions in a substantial amount of cells, increasing chase times reveals changes in S-phase patterns. This approach allowed us to get a spatio-temporal resolution of DNA replication in fixed cells. **(C)** Schematic representation and representative confocal images of a pulse-chase-pulse-chase experiment, underlining the domino-like DNA replication model. Scale bars = 5 µm.

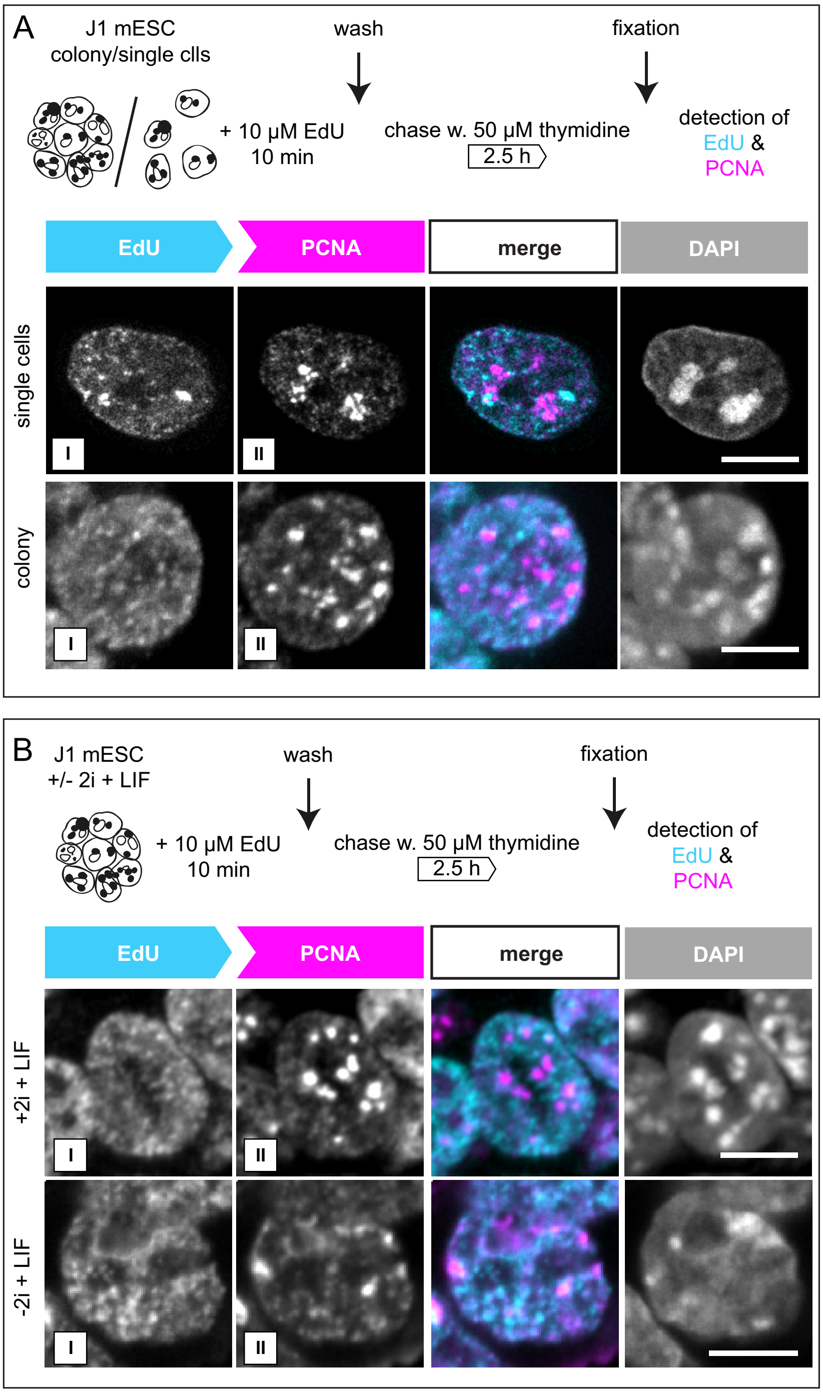

Supplementary Figure S10 – DNA replication dynamics of mouse embryonic cells in different microenvironments. (A) J1 mES cells were pulse chased as described in Figure 1, 6 hours (single cells) or 24 hours (colonies) after seeding, EdU and PCNA were (immuno)fluorescently detected and S-phase progression was analyzed. Representative confocal images of cells passing from stage I to stage II are depicted for single cells and cells grown in colonies. **(B)** J1 mouse ES cells were grown for 15 days in medium containing only LIF or 2i and LIF and pulse chased 24 hours after seeding. Confocal images of S-phase progression from stage I to stage II are depicted as above. Scale bars = 5 µm.

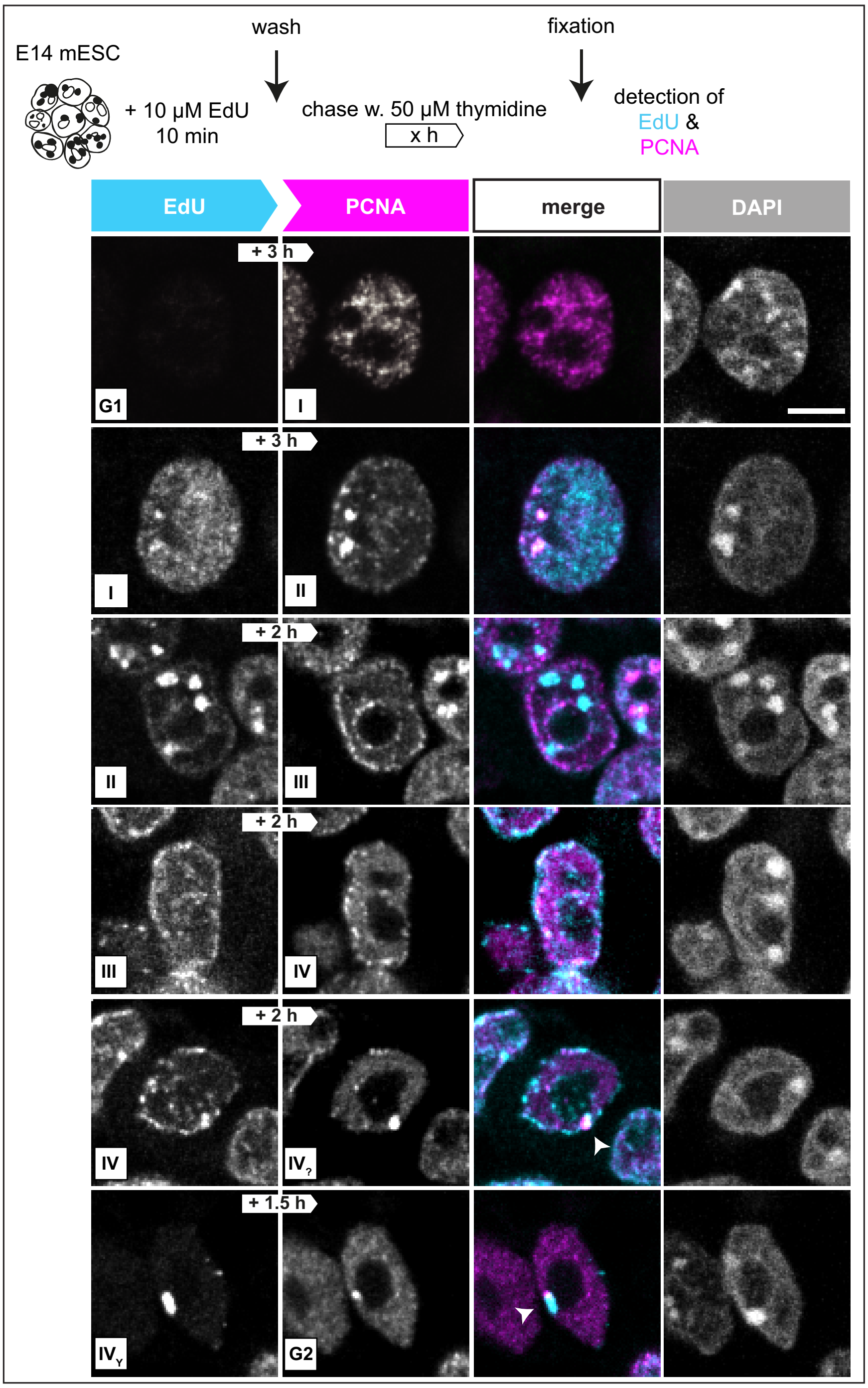

Supplementary Figure S11 – DNA replication dynamics in mouse E14 embryonic stem cells. The experimental pulse chase setup (described in Figure 1), allowed the identification and temporal order of five main replication patterns in mouse E14 ES cells. Representative spinning disk confocal images of transitions from G1 to S-phase, S-phase substage transitions and S-phase to G2 progression are shown. The arrowheads in the $IV_?$ stage mark a prominent accumulation of replication signals observed at the end of S-phase. White arrows represent chase periods. Scale bar = 5 µm.

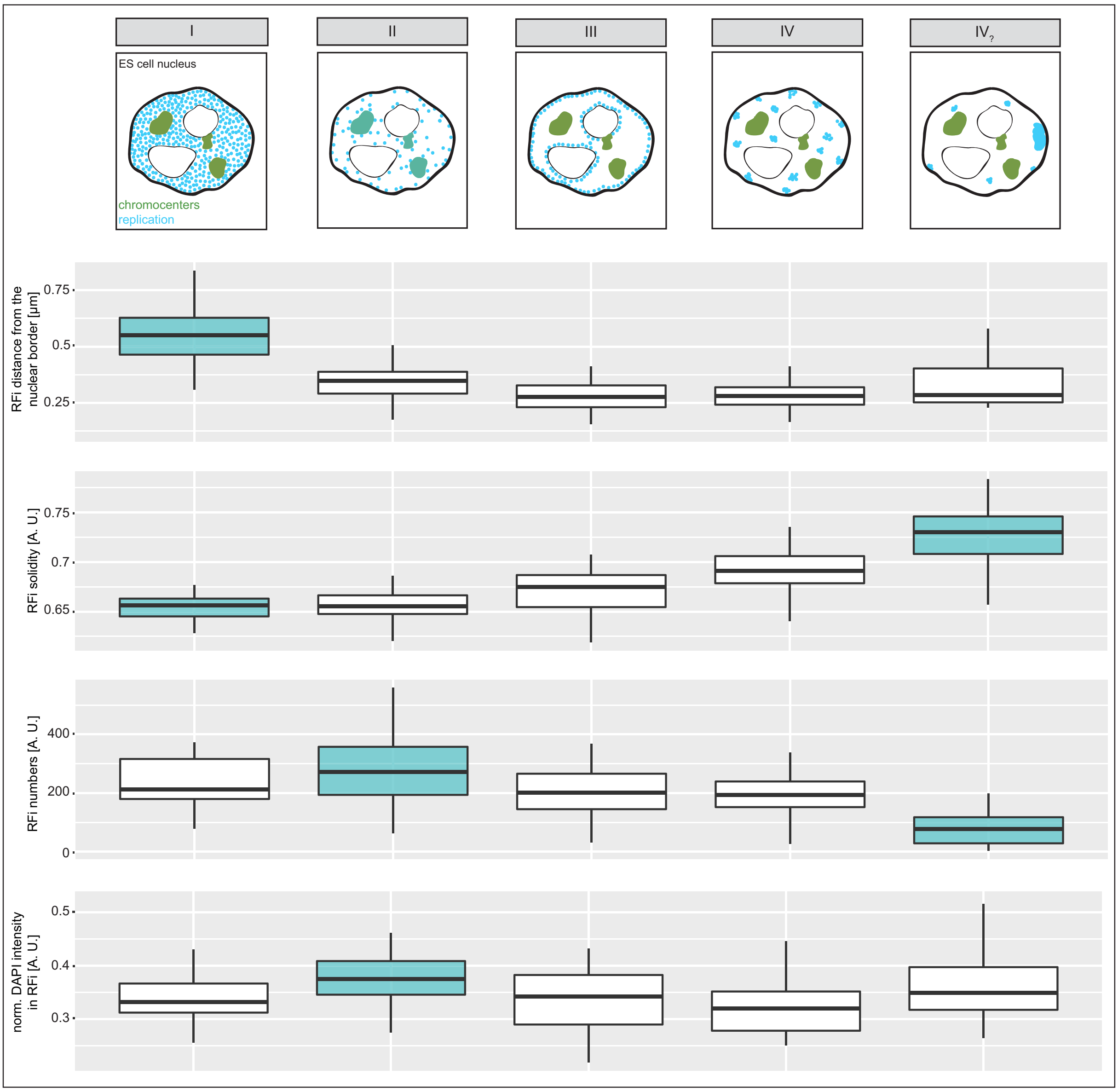

Supplementary Figure S12 – Characteristics of replication foci in the five S-phase substages of mouse embryonic stem cells. Replication foci (RFi) distance from the nuclear border, RFi solidity, RFi numbers and normalized DAPI intensities within RFi are plotted for the five different mES cell S-phase stages. Cyan marked boxplots represent features that differ significantly from one stage to the other. All boxplots are as in Figure 2 and Supplementary Figure S7. Detailed statistics are summarized in Supplementary Table 7.

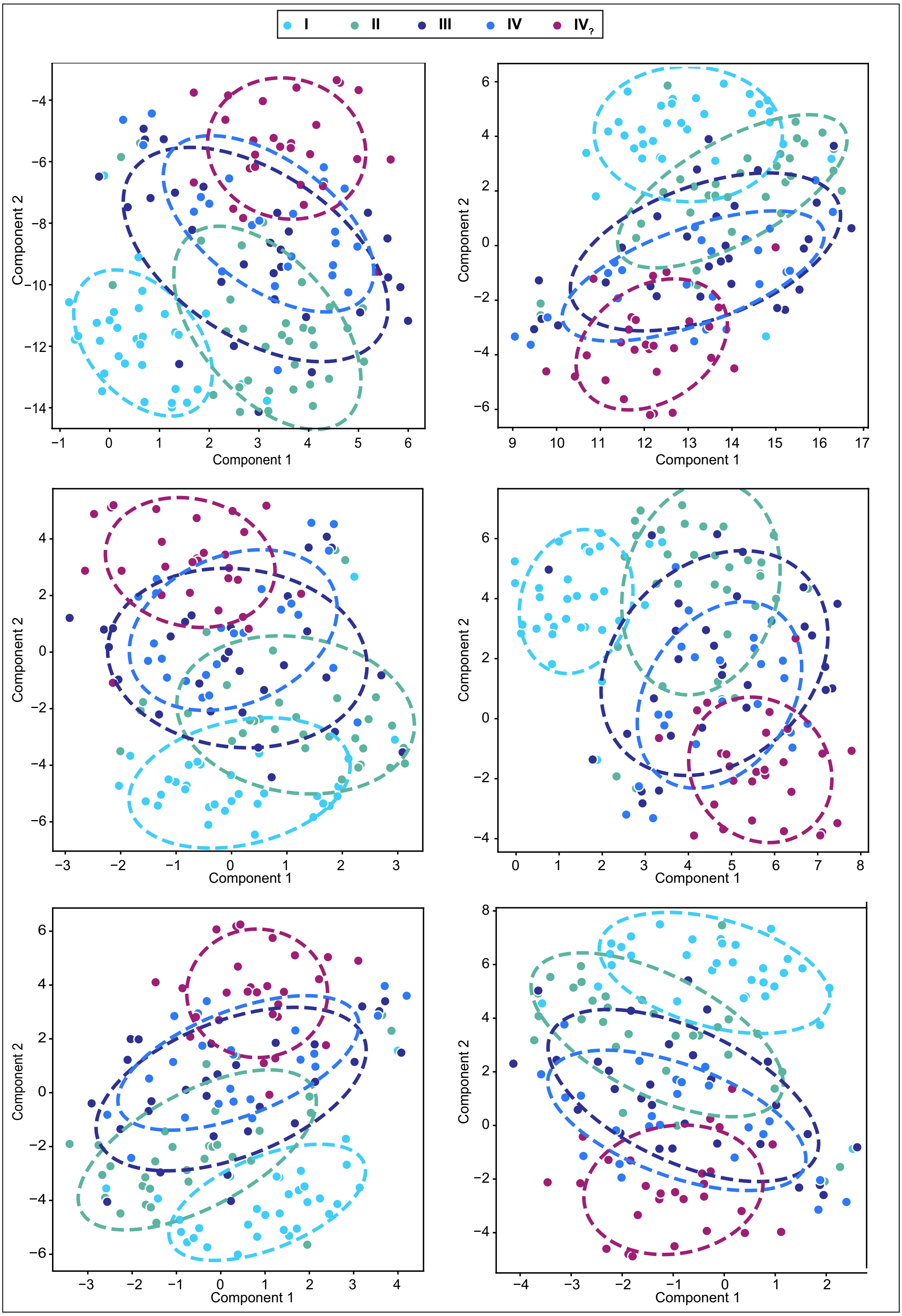
I
II
III
IV
IV?
Component 1
Component 2

Supplementary Figure S13 – t-distributed stochastic neighbor embedding (t-SNE) analysis and replication foci (RFi) characteristics distributions. (A) t-SNE embedding was used to visualize the high-dimensional RFi characteristics. Each high-dimensional object was modeled by a 2 dimensional point. Similar objects are modeled by nearby points and dissimilar objects are modeled by distant points. Six variants of t-SNE representations are shown. Multinomial logistic regression of the S-phase stage from the image features reached >70% accuracy. When also allowing for one-off errors (e.g. stage I classified as II), the accuracy was over >90% (accuracy determined in 5-fold cross-validation).

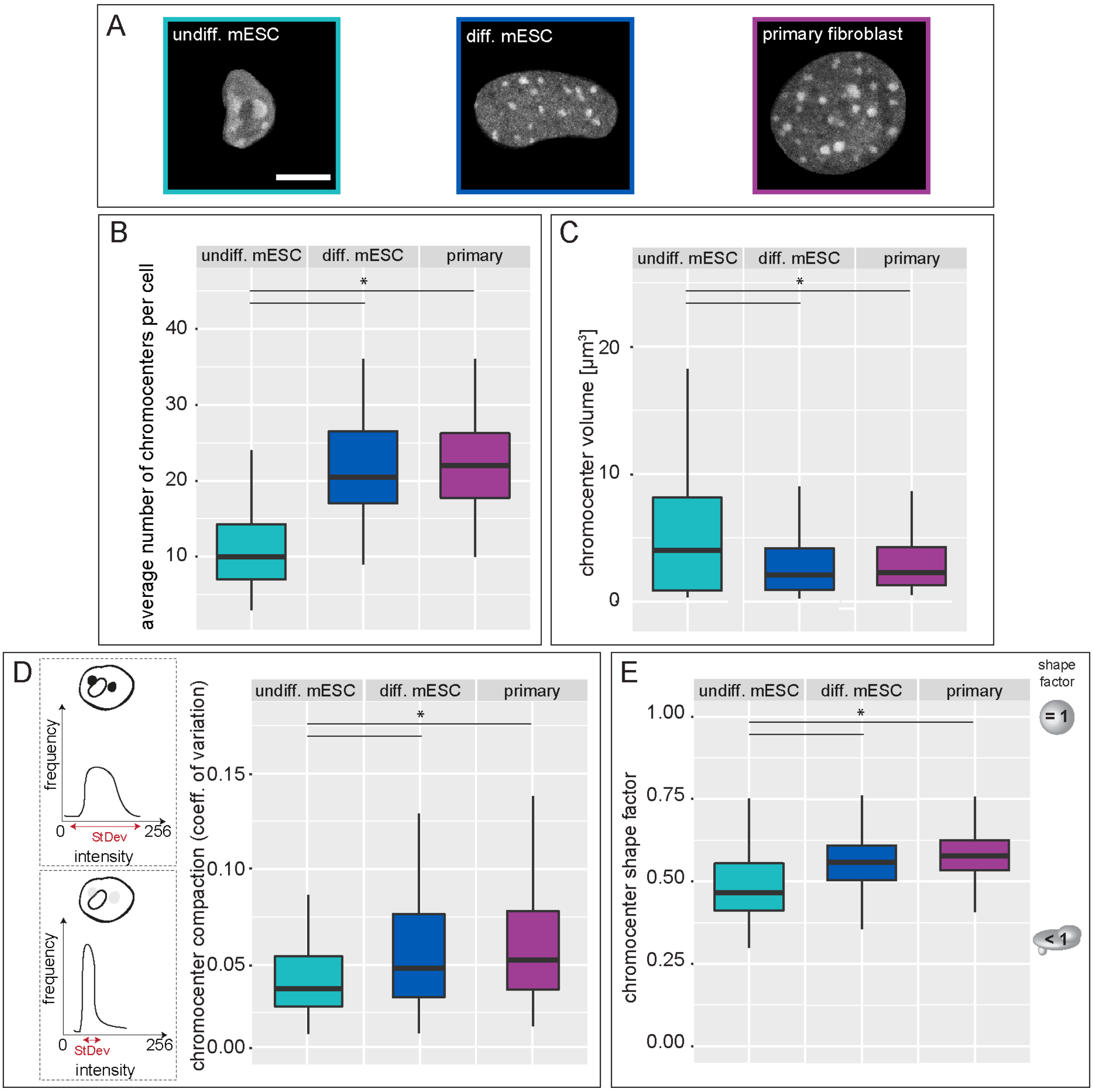

Supplementary Figure S14 – Characteristics of pericentromeric DNA clusters (chromocenters) in (un)differentiated mouse embryonic stem cells and mouse myoblast cells. (A) Representative cell nuclei from undifferentiated J1 mES cells (cyan), differentiated J1 mES cells (blue) and primary mouse ear cells (magenta). Average numbers of chromocenters per cell **(B),** volumes of chromocenters **(C)**, chromocenter compaction (coefficient of variation of DAPI (StDev/mean, **(D)**) and chromocenter shape factor **(E)** show the different organization of pericentromeric heterochromatin in mouse somatic and pluripotent mES cells. In **(D),** condensed chromatin is characterized by high DAPI intensities. Thus, cell nuclei containing condensed chromatin show a higher DAPI standard deviation than cells containing decondensed chromatin, which is characterized by low DAPI standard deviations. Normalized DAPI standard deviation values, as a proxy for DNA decondensation levels, are plotted. All boxplots are as in Figure 2 and Supplementary Figure S7. Detailed statistics are summarized in Supplementary Table 9. Scale bar = 5 µm. * *P* < 0.05.

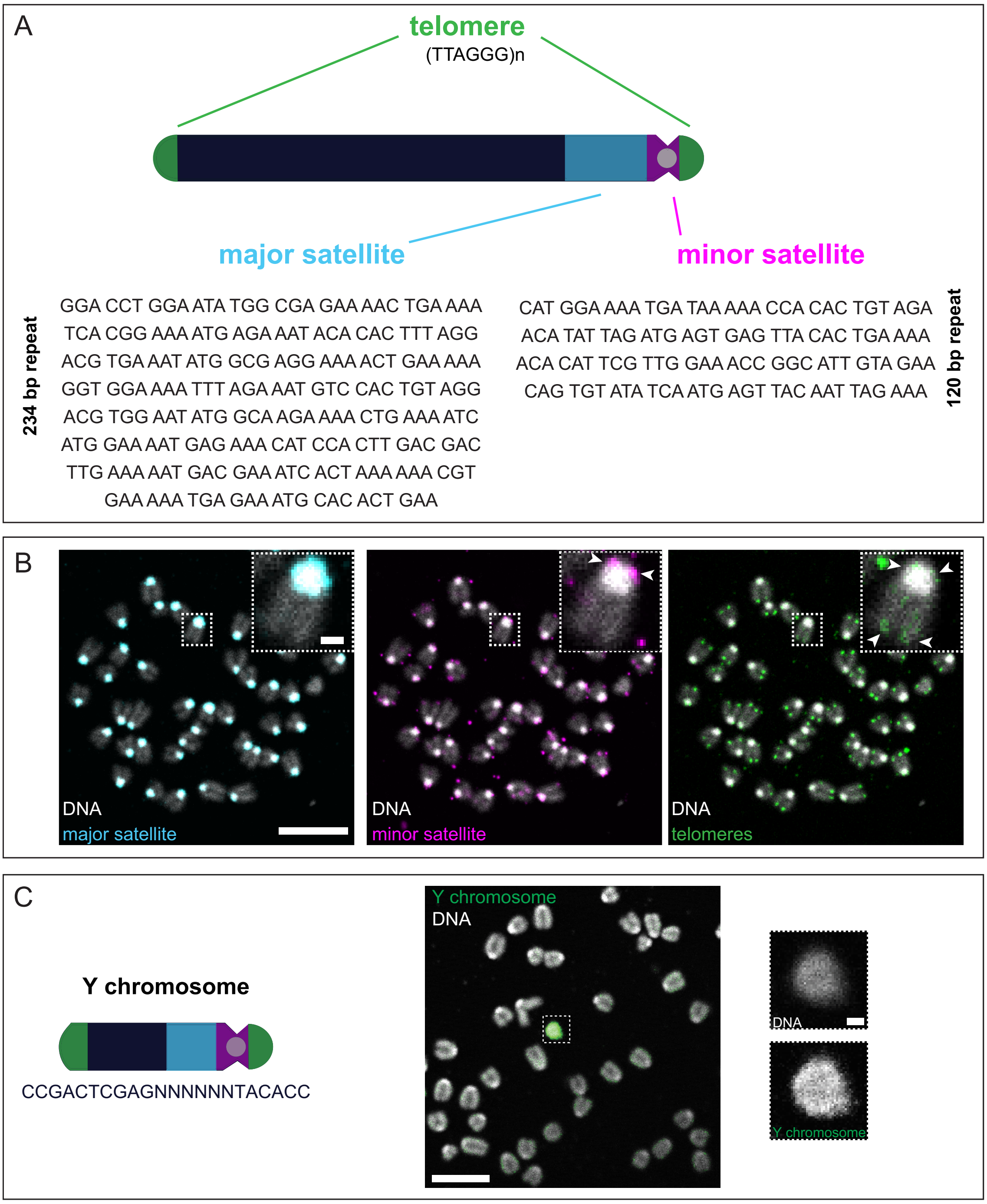

Supplementary Figure S15 – Validation of FISH probes for (sub)-chromosomal elements. (A) Schematic overview of the chromosomal location and sequence motifs of the analyzed major and minor satellites (MaSat (cyan) and MiSat (magenta)) and telomere (green) repeat elements in mouse cells. **(B)** Triple-color FISH combining probes labeled with different nucleotide analogs, specific for major satellites (cyan), minor satellites (magenta) and telomeres (green) hybridized to metaphase chromosome preparations from diploid mouse cells. Insets show a magnification of one chromosome and arrows show specific probe binding. **(C)** Schematic overview of the mouse Y chromosome and the primer sequence used for probe generation (left). Hybridization of the Y chromosome specific probe to mouse metaphase spreads. Inset shows specific probe annealing to one chromosome (right). Scale bar = 10 µm and 1 µm for main graphs and magnified regions, respectively.

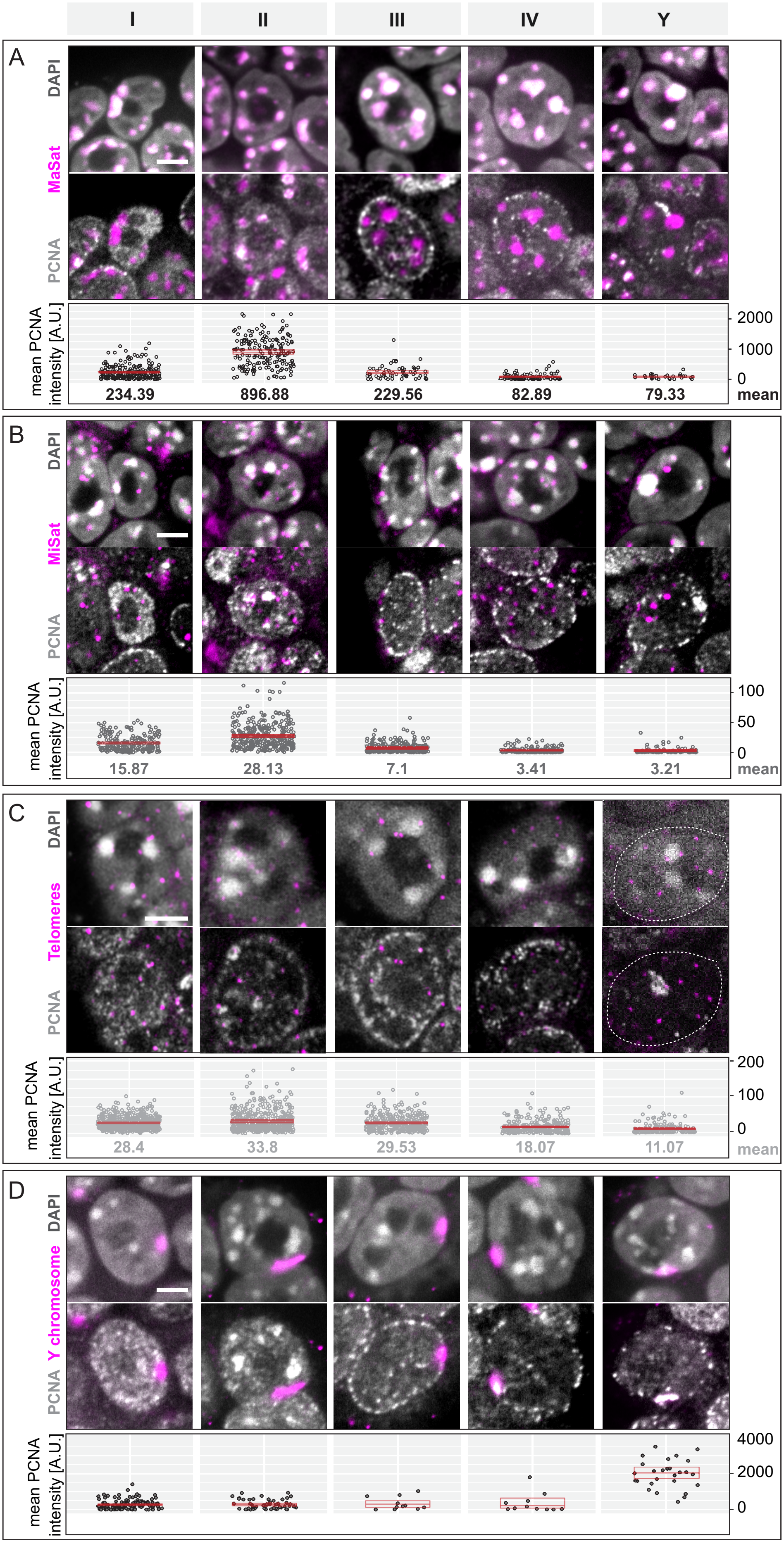

I
II
III
IV
Y
A
DAPI
MaSat
PCNA
mean PCNA intensity [A.U.]
2000
1000
0
234.39
896.88
229.56
82.89
79.33
mean
B
DAPI
MiSat
PCNA
mean PCNA intensity [A.U.]
100
50
0
15.87
28.13
7.1
3.41
3.21
mean
C
DAPI
Telomeres
PCNA
mean PCNA intensity [A.U.]
200
100
0
28.4
33.8
29.53
18.07
11.07
mean
D
DAPI
Y chromosome
PCNA
mean PCNA intensity [A.U.]
4000
2000
0

Supplementary Figure S16 – Replication timing of (sub-)chromosomal structures in mouse embryonic stem cells. Tandem repeat elements (magenta, major satellites (MaSat, **(A)**), minor satellites (MiSat, **(B)**) and telomeres **(C)**) and the Y chromosome **(D)** were co-visualized with PCNA (grey) via Repli-FISH in mES interphase cells. Cells were classified into S-phase stages I to Y according to their PCNA pattern, mean PCNA intensities within the marked elements were measured as described in Supplementary Figure S3 and plotted. Red boxes represent mean ± 95% confidence intervals and mean values are indicated below each plot. DAPI (grey) and hybridization signals (magenta) are shown as a merge. Dotted lines represent cell contours. Detailed statistics are summarized in Supplementary Table 10. Scale bar = 5 µm.

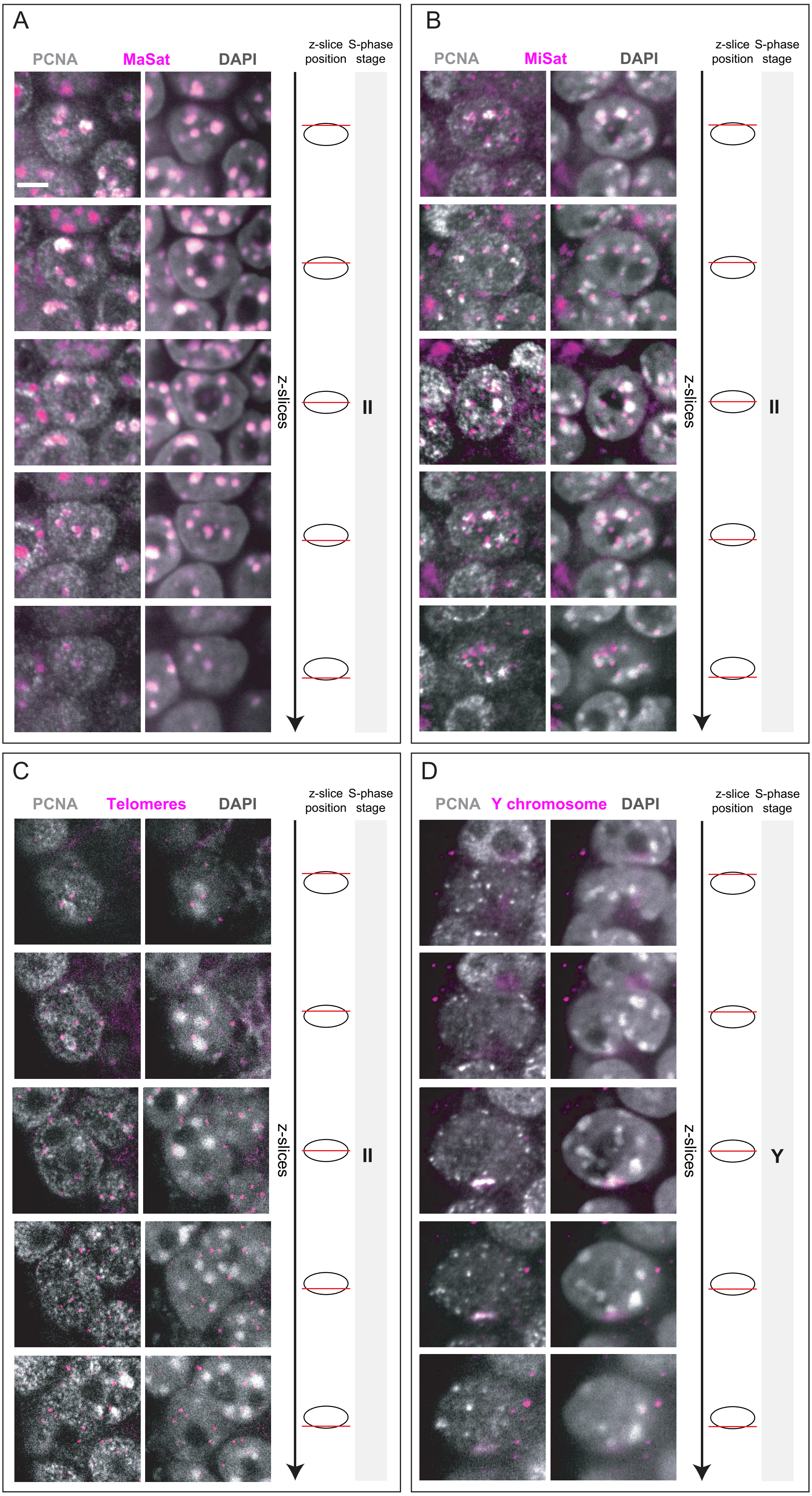
A
PCNA
MaSat
DAPI
z-slice position
S-phase stage
z-slices
II
B
PCNA
MiSat
DAPI
z-slice position
S-phase stage
z-slices
II
C
PCNA
Telomeres
DAPI
z-slice position
S-phase stage
z-slices
II
D
PCNA
Y chromosome
DAPI
z-slice position
S-phase stage
z-slices
Y

Supplementary Figure S17 – 3-dimensional nuclear distribution of (sub-)chromosomal structures in mouse embryonic stem cells. Tandem repeat elements (magenta, major satellites (MaSat, **(A)**), minor satellites (MiSat, **(B)**) and telomeres **(C)**) and the Y chromosome **(D)** were co-visualized with PCNA (grey, left column) or DAPI (grey, right column). Mid z slice from stage II cells (A-C) or stage Y (D) depicted in Supplementary Figure S16 are shown. Additionally 2 slices above and two slices below the mid slice are represented to show the distribution of the marked elements within the mES cell nucleus. Scale bar = 5 µm.

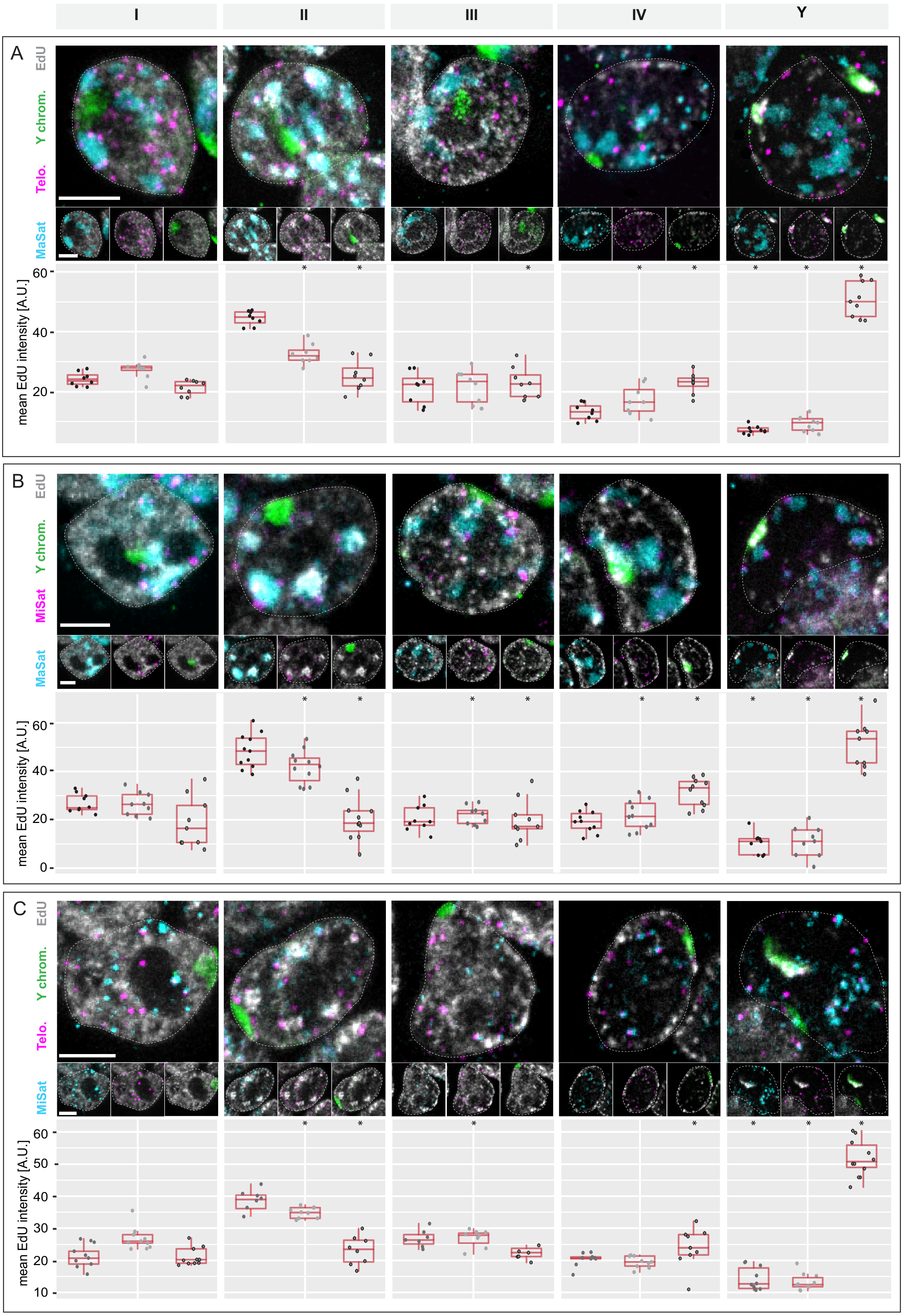
I
II
III
IV
Y
A
EdU
Y chrom.
Telo.
MaSat
mean EdU intensity [A.U.]
60
40
20
B
EdU
Y chrom.
MiSat
MaSat
mean EdU intensity [A.U.]
60
40
20
0
C
EdU
Y chrom.
Telo.
MiSat
mean EdU intensity [A.U.]
60
50
40
30
20
10

Supplementary Figure S18 – Replication timing of subchromosomal structures and the Y chromosome in mouse embryonic stem cells. EdU (DNA replication) and the Y chromosome were co-visualized with major satellite repeats and telomeres **(A)**, with major and minor satellite repeats **(B)** and with minor satellite repeats and telomeres **(C)** in mES cell interphase nuclei**.** Cells were classified into S-phase stages I to Y according to their EdU pattern, mean EdU intensities within the marked elements were measured as described in Supplementary Figure S3 and plotted. Detailed statistics are summarized in Supplementary Table 10. Scale bar = 5 µm. * $P < 0.05$ (calculated among the same element against the respective stage I value).

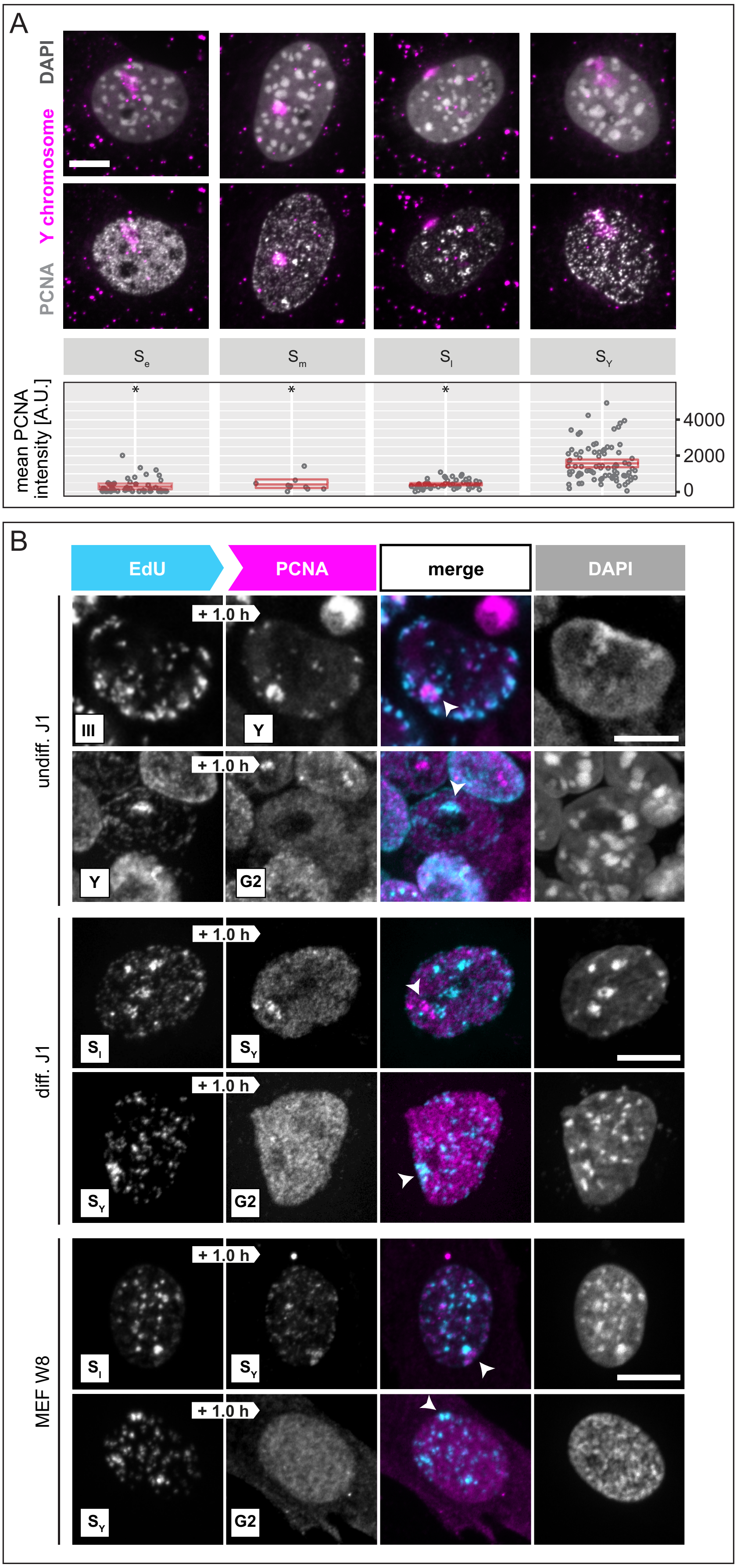
A
DAPI
Y chromosome
PCNA
Se
Sm
Sl
SY
mean PCNA intensity [A.U.]
4000
2000
0
B
EdU
PCNA
merge
DAPI
+ 1.0 h
undiff. J1
diff. J1
MEF W8
III
Y
G2
Sl
SY

Figure S19 – Y chromosome replication marks the end of S-phase independently of the cell potency state. (A) Replication timing analysis of the Y chromosome in mouse embryonic fibroblasts (MEF) via Repli-FISH. PCNA quantification within hybridization signals and representation are as in Figure 4. **(B)** Pulse chase experiment in male (un)differentiated mouse J1 ES cells and mouse embryonic fibroblasts (MEF W8). Representative spinning disk confocal images of transitions from stage III/late to stage Y and from stage Y to G2 are shown for all three cell lines. Arrowheads mark the Y chromosome. Boxplots are as in Figure 2 and Supplementary Figure S7. Scale bar = 5 µm. Detailed statistics are summarized in Supplementary Table 10. * $P < 0.05$ (calculated against S_Y).

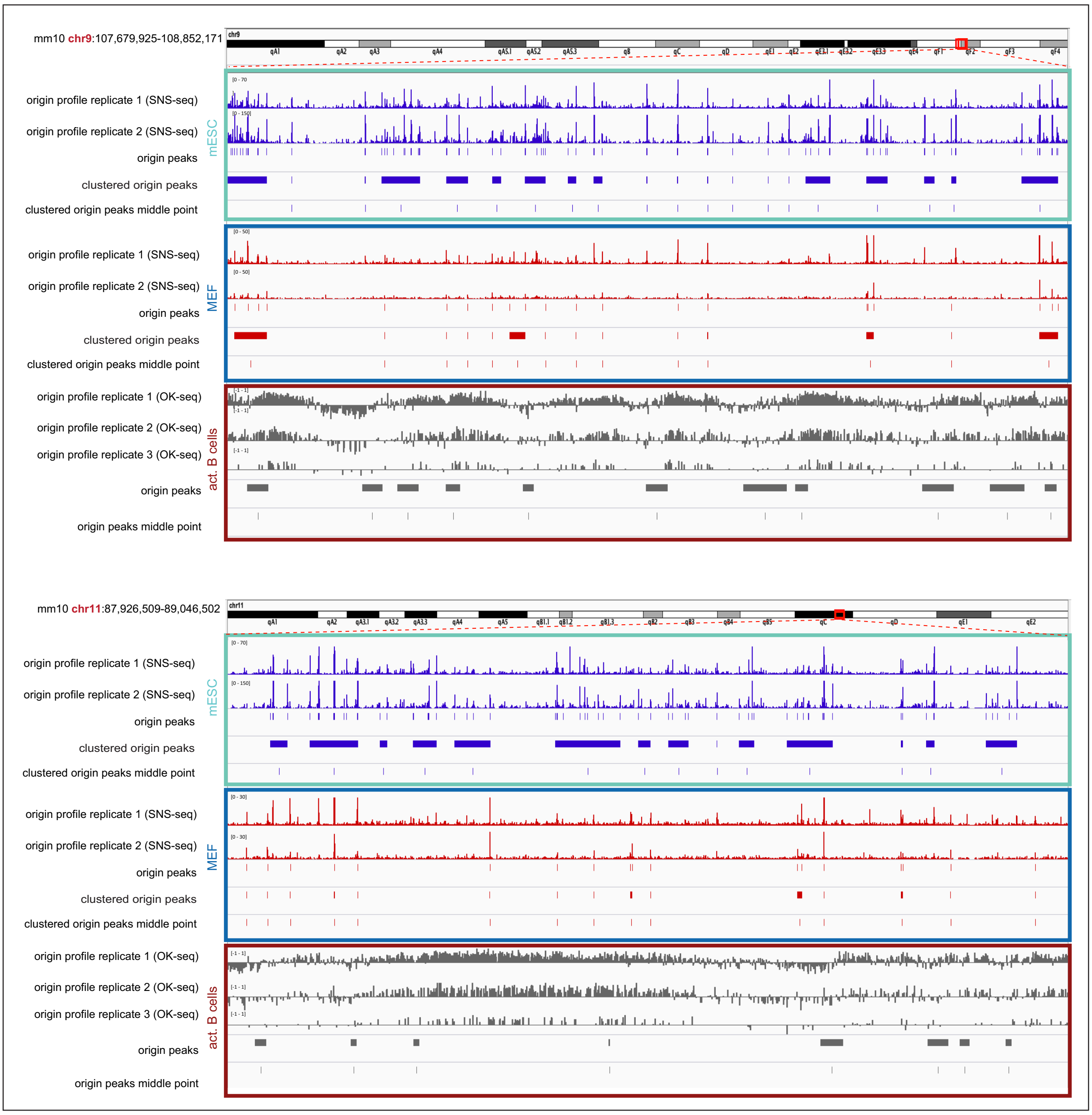

Supplementary Figure S20 – Origin profiling in mouse embryonic stem cells, mouse embryonic fibroblasts and mouse activated B cells. Graphical representation of origin profiling in mES, MEF and mouse activated B cells (act. B cells) in all replicates analyzed are shown. mES and MEF origins clustered at the distance of 22 kb (performed for equalization of origin mapping resolution in the SNS-seq and the OK-seq methods) as well as the middle point of clustered origins are indicated. The OK-seq profiles in the B cell line in 3 replicates are represented in the following rows as well as origins localization and their middle point. The replication profile scale is indicated in the upper left corner.

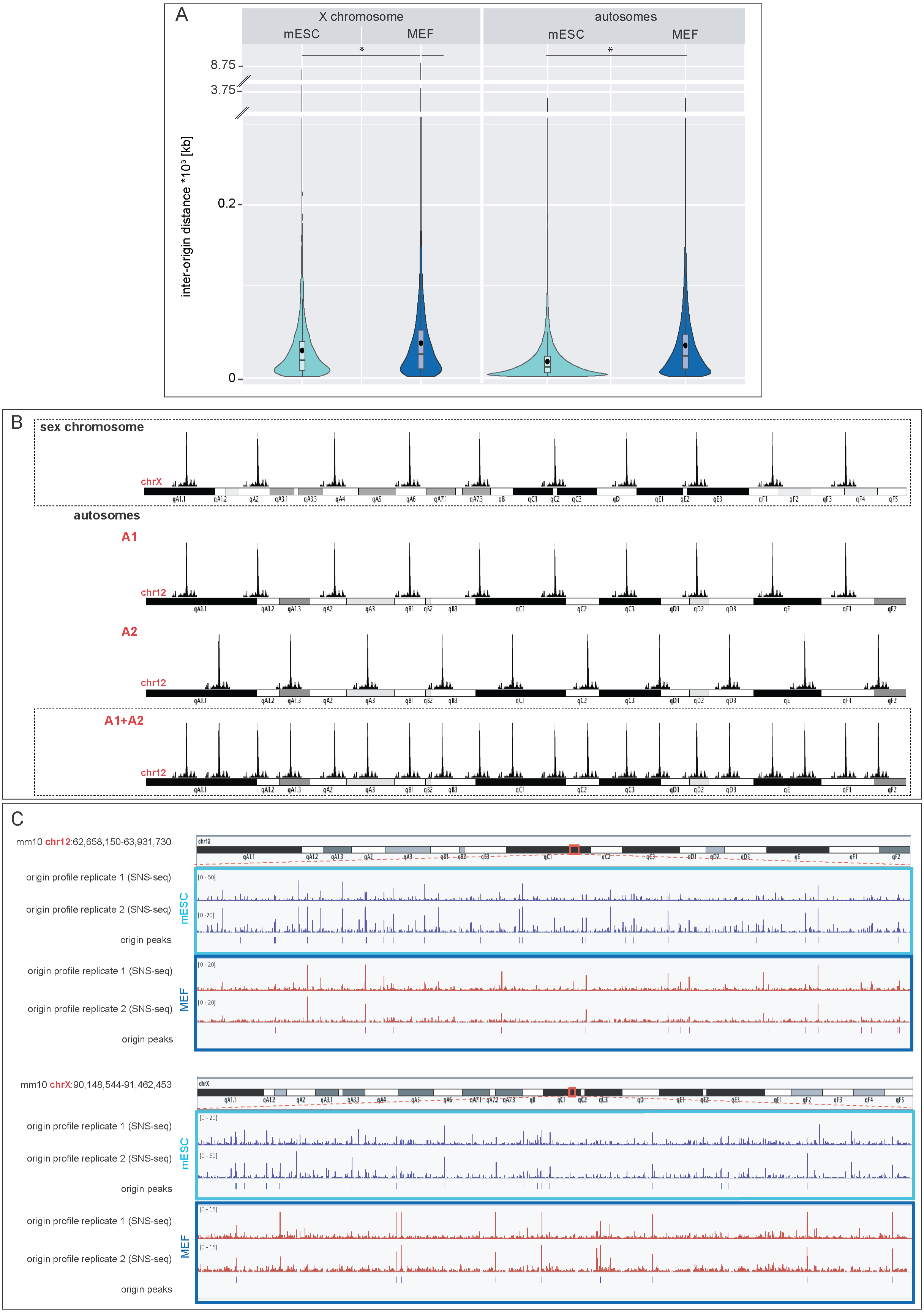
A
X chromosome
autosomes
mESC
MEF
mESC
MEF
*
*
8.75
3.75
0.2
0
inter-origin distance *10³ [kb]
B
sex chromosome
chrX
autosomes
A1
chr12
A2
chr12
A1+A2
chr12
C
mm10 chr12:62,658,150-63,931,730
origin profile replicate 1 (SNS-seq)
origin profile replicate 2 (SNS-seq)
origin peaks
mESC
origin profile replicate 1 (SNS-seq)
origin profile replicate 2 (SNS-seq)
origin peaks
MEF
mm10 chrX:90,148,544-91,462,453
origin profile replicate 1 (SNS-seq)
origin profile replicate 2 (SNS-seq)
origin peaks
mESC
origin profile replicate 1 (SNS-seq)
origin profile replicate 2 (SNS-seq)
origin peaks
MEF

Supplementary Figure S21 – Origin distribution on the chromosome X and autosomal chromosomes. (A) The comparison of IOD calculated for the mES and MEF cell lines on autosomes and the chromosome X using the SNS-seq origin mapping data. **(B)** Schematic representation of origin distribution on the chromosome X and 2 parental copies of autosomal chromosomes. **(C)** Graphical representation of origin profiling in mES and MEF cells on a somatic chromosome (chromosome 12) and an arbitrary chosen region on the chromosome X. Two independent replicates of origin profiling and the localization of origins reproducibly identified in the two replicates are shown. The replication profile scale is indicated in the upper left corner. Detailed statistics are summarized in Supplementary Table 13. * $P < 0.05$.

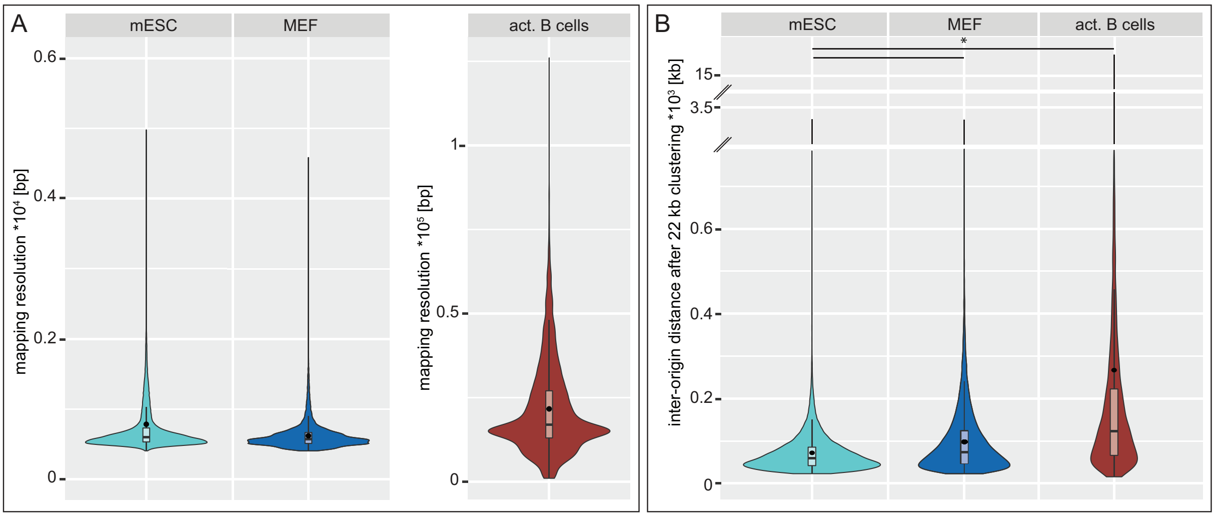

Supplementary Figure S22 – Origin mapping resolution in genome-wide techniques and origin clustering. (A) Origin mapping resolutions measured as an average origin peak width in SNS-seq and OK-seq methods are shown. **(B)** For comparison purposes the differences in methods resolution were equalized by merging the neighboring origins detected in SNS-seq in the distance of the average peak size in OK-seq analysis (22 kb). The IODs calculated for clustered origins in mES and MEF cell lines as well as origins identified in activated B cells (act. B cells) are shown. Detailed statistics are summarized in Supplementary Table 13. * $P < 0.05$.

Movies 1 & 2 - Time lapse movie of cell cycle progression in a mouse embryonic stem cell. mES cells were transfected with mRFP-PCNA and MaSat-GFP (polydactyl zinc finger protein with specific binding to major satellite repeats) and time lapse confocal microscopy was performed 24 hours post-transfection. z-stacks were acquired at 30 minutes intervals. Chromocenters (green) and DNA replication (magenta) are shown as merged images and time intervals are shown on the upper left. Depicted mES cell progresses from S-phase stage I to mitosis (movie 1) and from S-phase stage I to G2 (movie 2). Scale bar = 5 µm.

REFERENCES

1. Li, E., Bestor, T.H. and Jaenisch, R. (1992) Targeted mutation of the DNA methyltransferase gene results in embryonic lethality. *Cell*, **69**, 915-926.

2. Doetschman, T., Gregg, R.G., Maeda, N., Hooper, M.L., Melton, D.W., Thompson, S. and Smithies, O. (1987) Targetted correction of a mutant HPRT gene in mouse embryonic stem cells. *Nature*, **330**, 576-578.

3. Yaffe, D. and Saxel, O. (1977) Serial passaging and differentiation of myogenic cells isolated from dystrophic mouse muscle. *Nature*, **270**, 725-727.

4. Peters, A.H., O'Carroll, D., Scherthan, H., Mechtler, K., Sauer, S., Schofer, C., Weipoltshammer, K., Pagani, M., Lachner, M., Kohlmaier, A. *et al.* (2001) Loss of the Suv39h histone methyltransferases impairs mammalian heterochromatin and genome stability. *Cell*, **107**, 323-337.

5. Sporbert, A., Domaing, P., Leonhardt, H. and Cardoso, M.C. (2005) PCNA acts as a stationary loading platform for transiently interacting Okazaki fragment maturation proteins. *Nucleic acids research*, **33**, 3521-3528.

6. Lindhout, B.I., Fransz, P., Tessadori, F., Meckel, T., Hooykaas, P.J. and van der Zaal, B.J. (2007) Live cell imaging of repetitive DNA sequences via GFP-tagged polydactyl zinc finger proteins. *Nucleic acids research*, **35**, e107.

7. Casas-Delucchi, C.S., Becker, A., Bolius, J.J. and Cardoso, M.C. (2012) Targeted manipulation of heterochromatin rescues MeCP2 Rett mutants and re-establishes higher order chromatin organization. *Nucleic acids research*, **40**, e176.

8. Weber, P., Rausch, C., Scholl, A. and Cardoso, M.C. (2019) Repli-FISH (Fluorescence in Situ Hybridization): Application of 3D-(Immuno)-FISH for the study of DNA replication timing of genetic repeat elements. *OBM Genetics*, **3**.

9. Prorok, P., Artufel, M., Aze, A., Coulombe, P., Peiffer, I., Lacroix, L., Guedin, A., Mergny, J.L., Damaschke, J., Schepers, A. *et al.* (2019) Involvement of G-quadruplex regions in mammalian replication origin activity. *Nature communications*, **10**, 3274.

10. Almeida, R., Fernandez-Justel, J.M., Santa-Maria, C., Cadoret, J.C., Cano-Aroca, L., Lombrana, R., Herranz, G., Agresti, A. and Gomez, M. (2018) Chromatin conformation regulates the coordination between DNA replication and transcription. *Nature communications*, **9**, 1590.

11. Tubbs, A., Sridharan, S., van Wietmarschen, N., Maman, Y., Callen, E., Stanlie, A., Wu, W., Wu, X., Day, A., Wong, N. *et al.* (2018) Dual Roles of Poly(dA:dT) Tracts in Replication Initiation and Fork Collapse. *Cell*, **174**, 1127-1142 e1119.
